# Glutamate nanoregulator for metabolic immunotherapy of biofilm-associated implant infections

**DOI:** 10.1186/s12951-025-04016-3

**Published:** 2026-02-04

**Authors:** Heng Wu, Jiahao Chen, Xiao Ma, Haijian Li, Qiao Wu, Zhenyu Jiang, Tianyu Xi, Chi Zhang, Geyong Guo, Pei Han

**Affiliations:** 1 Department of Orthopedics, Shanghai Sixth People’s Hospital, Shanghai Jiao Tong University School of Medicine, Shanghai Jiao Tong University, No. 600 Yishan Road, Xuhui District, Shanghai, 200233 P. R. China; 2 Department of Orthopedics, Shanghai First People’s Hospital, Shanghai Jiao Tong University School of Medicine, Shanghai Jiao Tong University, No. 85 Wujin Road, Hongkou District, Shanghai, 200071 P. R. China

**Keywords:** Glutamate metabolism, Sulfate radical, Dendritic cells, Biofilm-associated infections, Metabolic immunotherapy

## Abstract

**Background:**

Implant-associated infections caused by *Staphylococcus aureus* biofilms remain difficult to eradicate because the extracellular matrix and metabolic heterogeneity jointly suppress antibiotic penetration and immune clearance. The biofilm environment induces excessive interleukin-10 (IL-10) production in dendritic cells, leading to inhibition of the cGAS–STING signaling pathway and T-cell activation. Therefore, strategies that simultaneously disrupt biofilm structure and reverse the immunosuppressive microenvironment are urgently needed.

**Results:**

We developed a near-infrared–responsive MnO₂@PMS nanoregulator that integrates photothermal catalysis with metabolic immunotherapy. Upon laser irradiation, MnO₂@PMS generated oxygen-independent sulfate radicals (•SO₄⁻) that efficiently degraded the biofilm matrix and suppressed bacterial glutamate metabolism, thereby reducing the synthesis of poly-γ-glutamic acid. This metabolic inhibition decreased biofilm-derived glutamate accumulation and downregulated IL-10 production in dendritic cells, leading to reactivation of the cGAS–STING pathway and restoration of antigen presentation. In vivo, MnO₂@PMS treatment promoted mature dendritic-cell and T-cell activation, reduced bacterial burden, alleviated local inflammation, and enhanced angiogenesis and tissue repair, while exhibiting favourable short-term biocompatibility in vivo.

**Conclusions:**

This study introduces a glutamate-targeted nanoplatform that couples biofilm destruction with immune reprogramming in a chronic biofilm infection model. By bridging metabolic regulation and immune activation, MnO₂@PMS complements existing nanozyme and photothermal antibiofilm strategies by illustrating a mechanistically supported approach that integrates oxygen-independent sulfate radical catalysis with modulation of glutamate–IL-10–associated immunosuppression.

**Graphical abstract:**

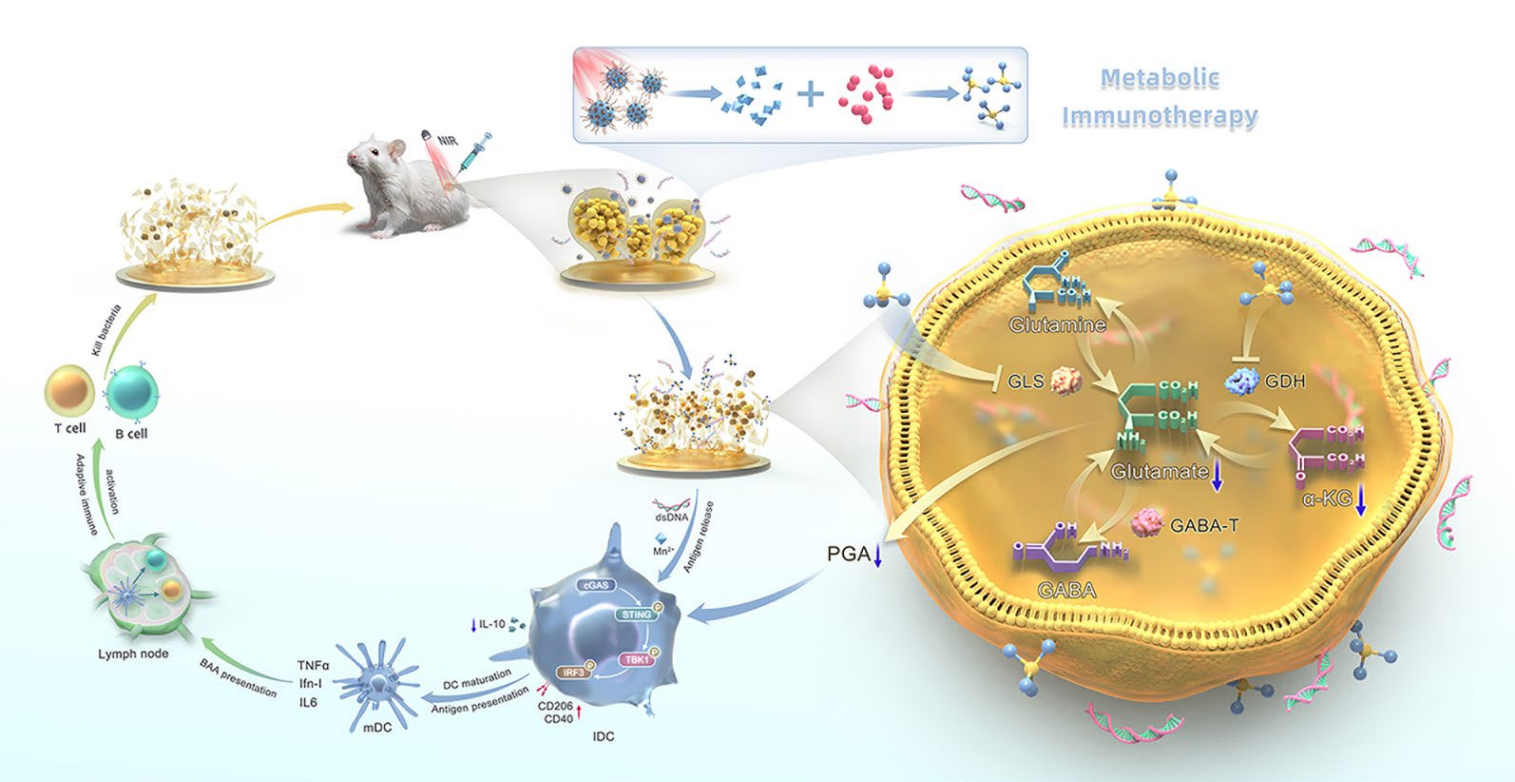

**Supplementary Information:**

The online version contains supplementary material available at 10.1186/s12951-025-04016-3.

## Background

Implant-associated infections caused by *Staphylococcus aureus* biofilms remain a formidable clinical challenge. These infections are difficult to eradicate because of both a physical extracellular polymeric substance (EPS) barrier composed of extracellular DNA (eDNA), proteins, and polysaccharides, and a metabolic barrier (low pH, oxygen depletion, and restricted molecular diffusion) that together protect bacteria from antibiotics and immune clearance [1, 2]. Moreover, mature biofilms elicit a dysregulated immune response characterized by impaired activation of innate immune cells, particularly dendritic cells, and elevated interleukin-10 (IL-10), a potent anti-inflammatory cytokine that suppresses phagocytic activity and antigen presentation [3, 4]. This IL-10–rich niche undermines cytosolic DNA-sensing pathways, particularly the cyclic GMP–AMP synthase–stimulator of interferon genes (cGAS–STING) axis, thereby impeding dendritic cells (DCs) maturation and T-cell priming [4, 5]. These immune defects explain the failure of conventional antibiotic strategies and underscore the need for therapeutic approaches that can simultaneously disrupt biofilms and reverse the associated immune suppression. In the present study, we therefore focus on mechanistically dissecting a metabolism-targeted nanoplatform in a proof-of-concept implant infection model, rather than performing an exhaustive head-to-head benchmarking against all existing clinical regimens.

Reactive oxygen species (ROS), including hydrogen peroxide (H₂O₂), superoxide anion (O₂•⁻), hydroxyl radical (•OH), and singlet oxygen (¹O₂), are well recognized for their broad antimicrobial activity via oxidative damage to biomacromolecules (DNA, proteins, and lipids). However, their efficacy is reduced in biofilm settings because of the antioxidant defense systems within biofilms, limited penetration through the extracellular polymeric matrix, and an oxygen-depleted microenvironment, impairing ROS generation. Within biofilms, hypoxia and matrix density restrict oxygen availability and molecular diffusion, rendering oxygen-dependent species such as hydroxyl radicals (•OH) largely ineffective [6, 7]. In addition, the extremely short half-life of •OH and its moderate redox potential further limit its activity within dense biofilm matrices. In contrast, sulfate radicals (•SO₄⁻), generated via peroxymonosulfate (PMS) activation, possess distinct physicochemical advantages: they are oxygen-independent, exhibit a higher standard redox potential (~ 2.6–3.1 V), and have longer lifespans, enabling deeper penetration into hypoxic and acidic biofilm niches [8]. These features make •SO₄⁻ particularly suitable for addressing both the structural and metabolic barriers of *S. aureus* biofilms. In addition manganese dioxide (MnO₂), under photothermal stimulation, catalyzes PMS decomposition to produce •SO₄⁻ radicals, while localized heating from photothermal conversion enhances radical diffusion and bactericidal activity [9, 10]. This dual mechanism allows more efficient bacterial killing under oxygen-depleted, diffusion-limited conditions, complementing conventional photodynamic therapy [6, 7, 10]. However, disrupting the biofilm’s structure alone may not achieve durable infection clearance, as the immunosuppressive niche can persist even after matrix breakdown [4, 5]. 

This persistence underscores the need to move beyond structural eradication: reprogramming the immunosuppressive microenvironment is critical for durable resolution. In biofilm-associated infections, accumulated bacterial metabolites profoundly shape immune tolerance, skewing myeloid cells toward an IL-10^high^ phenotype [4, 11]. The dense biofilm matrix not only limits antigen exposure but also promotes IL-10 accumulation, jointly suppressing DCs maturation and antigen presentation [3, 4]. Interestingly, the co-occurrence of biofilm eDNA and MnO₂-derived Mn²⁺ creates an opportunity for cGAS–STING activation, although this potential remains suppressed by IL-10 dominance [5, 12]. Among immunomodulatory metabolites, glutamate metabolism plays a dual role in *S. aureus* biofilm pathogenesis, acting as a precursor for poly-γ-glutamic acid (PGA) that strengthens the EPS matrix, while functioning as an immunoregulatory signal that induces IL-10 production in host immune cells [12]. Recent findings identify bacterial glutamate metabolism as a key immunometabolic checkpoint sustaining IL-10–mediated repression of the cGAS–STING pathway [12, 13]. Elevated IL-10 levels inhibit DCs activation, impairing antigen presentation and T-cell priming, which enables biofilms to evade immune clearance [13]. Therefore, nanotherapeutic strategies that can simultaneously disrupt glutamate metabolism and reverse immune suppression are urgently needed, a challenge requiring materials with dual catalytic and immunomodulatory functions. By collapsing this metabolic–immune checkpoint, innate immune responsiveness can be restored to enhance biofilm clearance [13, 14]. 

**Fig. 1:**
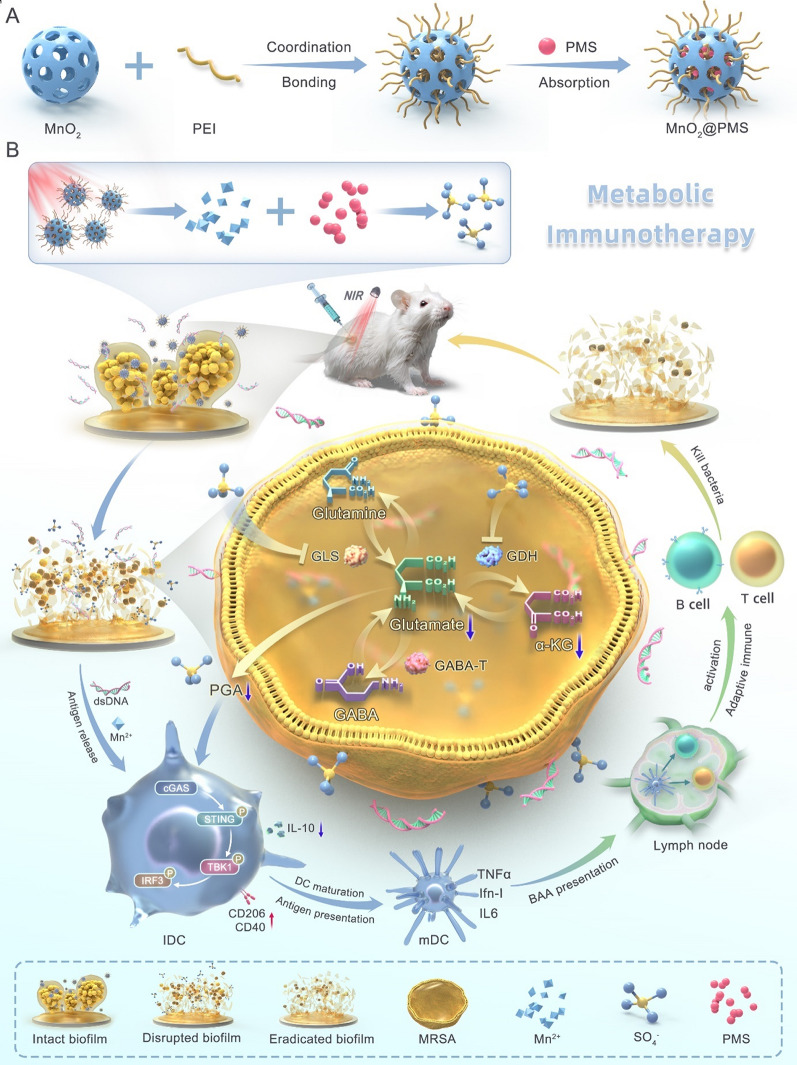
The design, construction, and working mechanism of the glutamate nanoregulator for biofilm disruption and metabolic immunotherapy of implant-associated infections. **(A)** Schematic illustration of MnO₂@PMS preparation: PEI-mediated coordination bonding to MnO₂ followed by PMS adsorption to obtain MnO₂@PMS. **(B)** Conceptual illustration of the dual-action therapeutic workflow/mechanism of MnO₂@PMS, integrating antibiofilm activity and immunometabolic regulation to enhance anti-infection immunity

To bridge this gap, we propose a metabolic immuno-nanoregulator (MnO₂@PMS) that simultaneously targets biofilm structural resilience and immunometabolic suppression through a photothermal–photosensitizer modality under near-infrared irradiation (Fig. 1). Building on prior MnO₂-based nanozyme and photothermal platforms that enhance reactive species generation and local hyperthermia in biofilm settings, this oxygen-independent oxidative strategy is designed to penetrate the hypoxic, acidic biofilm microenvironment, disrupt the EPS matrix, and limit bacterial PGA synthesis. Moreover, by intercepting bacterial glutamate metabolism, MnO₂@PMS mitigates an IL-10–associated immunosuppressive circuit and facilitates reactivation of the cGAS–STING pathway, restoration of antigen presentation, and local immune reprogramming. In this way, MnO₂@PMS illustrates a metabolic immunotherapy–oriented approach that integrates nanomaterial-enabled oxidative activity with modulation of glutamate–IL-10–related immunosuppression in *S. aureus* biofilms, complementing existing nanozyme and photothermal strategies rather than defining an entirely new therapeutic paradigm.

## Results and discussion

### Synthesis and multimodal ccharacterization of MnO₂@PMS nanocomposites

**Fig. 2:**
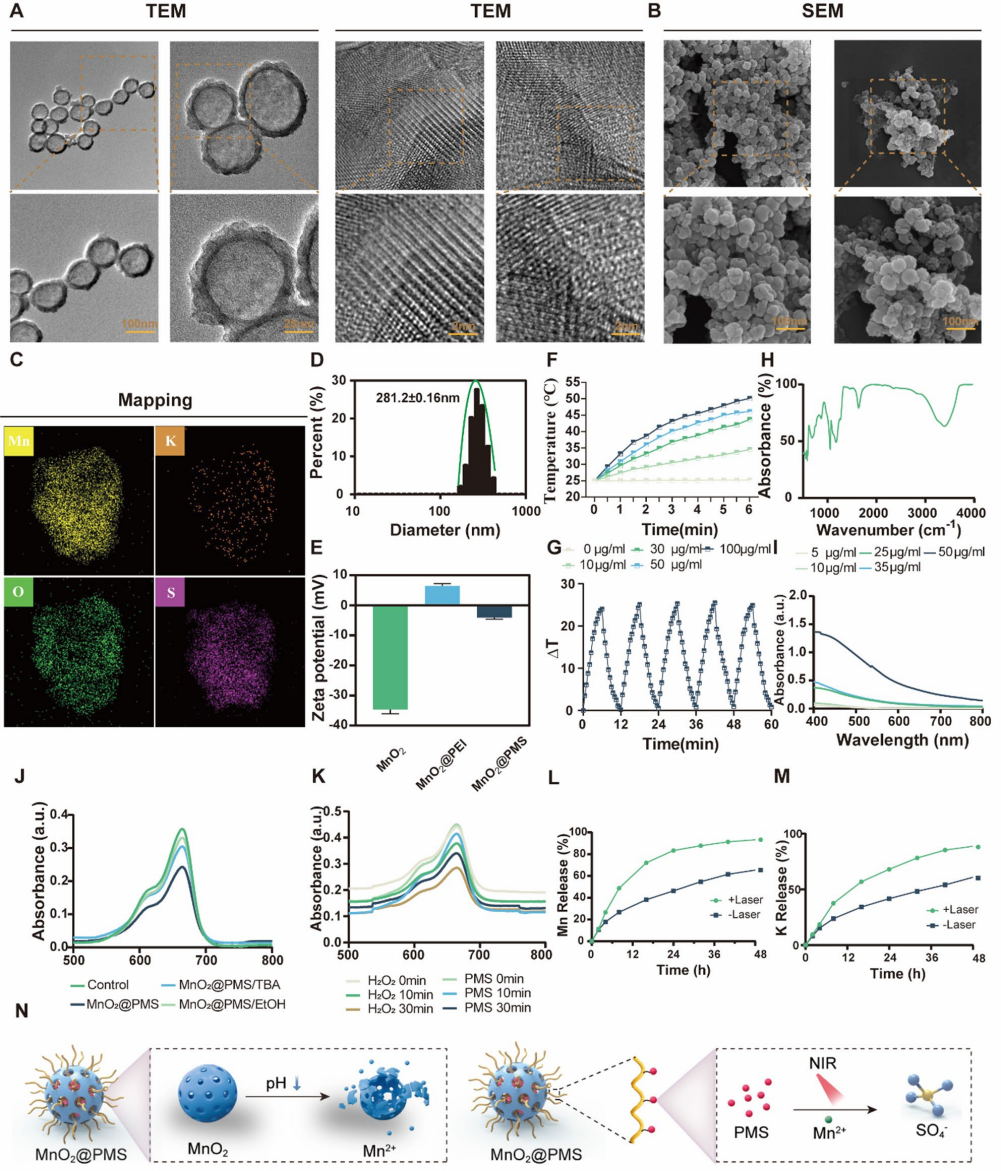
Physicochemical characterization of MnO₂@PMS nanocomposites. **(A)** Transmission electron microscopy (TEM) images of MnO₂@PMS particles showing uniform hollow spherical morphology and lattice structure. Scale bars: 100 nm, 25 nm, 2 nm. **(B)** Scanning electron microscopy (SEM) images revealing the rough and porous surface of MnO₂@PMS particles. Scale bar: 100 nm. **(C)** Energy-dispersive X-ray spectroscopy (EDX) elemental mapping demonstrating uniform distribution of Mn, K, O, and S. Scale bar: 20 μm. **D–E.** Dynamic light scattering (DLS) analysis showing particle size distribution (D) and zeta potential (E) of MnO₂, MnO₂@PEI, and MnO₂@PMS, indicating successful surface modification. **F–G.** Photothermal performance of MnO₂@PMS under 808 nm laser irradiation (1.0–1.5 W/cm²), demonstrating concentration-dependent temperature elevation (F) and reproducible heating/cooling cycles (G). **H.** Fourier-transform infrared (FTIR) spectrum indicating characteristic functional groups on the nanoparticle surface. **I.** UV–vis absorption spectra at different concentrations, highlighting broadband NIR absorbance of MnO₂@PMS. **J–K.** Time-dependent Mn²⁺ (J) and K⁺ (K) release under laser and dark conditions, reflecting photothermal-enhanced PMS decomposition and ion release. **L.** Electron paramagnetic resonance (EPR) spectra confirming generation of sulfate radicals (•SO₄⁻) using DMPO as the spin-trap reagent. **M.** Comparison of radical degradation under acidic conditions, showing the enhanced stability of •SO₄⁻ over hydroxyl radicals (•OH). **N.** Schematic illustration of MnO₂@PMS design involving pH-responsive MnO₂ decomposition, PEI-mediated PMS loading, and NIR-triggered catalytic activation leading to Mn²⁺ and •SO₄⁻ release

Uniform hollow MnO₂ nanospheres were obtained through a combination of commercial sourcing and template-assisted synthesis. The resulting particles exhibited well-defined cavities and porous shell structures (Fig. 2A–C). Transmission electron microscopy (TEM) images revealed spherical morphologies (approximately 100–150 nm in diameter) with a dark outer rim and lighter core, confirming hollow architectures. The shell thickness measured tens of nanometers and comprised ultrathin MnO₂ nanosheets or self-assembled lamellae, typical of manganese oxide–based hollow frameworks. This configuration provides a confined interior volume ideal for encapsulating guest molecules, in this case, peroxymonosulfate (PMS), which was post-loaded into the cavity via an impregnation strategy. Elemental mapping (Fig. 2C) showed strong Mn and O signals across the shell and detectable K and S signals throughout the nanospheres, consistent with the potassium peroxymonosulfate (KHSO₅) cargo. The co-localization of K and S with the MnO₂ framework confirmed successful PMS incorporation within the hollow nanostructures. This nanoconfinement prevents premature leakage while allowing access through the porous shell, enabling on-demand reactive species generation. The design aligns with recent advances in hollow Mn-based nanoplatforms, which exploit internal cavities for functional cargo encapsulation and MnO₂ shells for therapeutic functions [15]. Notably, our hollow MnO₂ nanostructure combines high surface area and efficient molecular diffusion with the intrinsic diagnostic and therapeutic properties of manganese chemistry, consistent with previously reported systems [15]. 

Surface morphology examined by scanning electron microscopy (SEM) (Fig. 2B) further corroborated the uniformity and porosity of the nanospheres. The surface appeared rough and textured, consistent with a mesoporous assembly of MnO₂ nanosheets. This texturing facilitates high PMS loading and rapid water penetration into the core. Dynamic light scattering (DLS) analysis showed an average hydrodynamic diameter of ~ 180 nm (Fig. 2E), slightly larger than TEM-derived sizes because of hydration and minor aggregation, but still sufficiently small for colloidal stability and diffusion in solution. The low polydispersity index (~ 0.2) indicated a monodisperse size distribution. Zeta potential measurements revealed moderately positive surface charges (+ 25.4 mV in phosphate-buffered saline [PBS] buffer). This positive ζ-potential was likely due to residual K⁺ adsorption or cationic surface modifications, deliberately enhancing bacterial interactions. Because bacterial cell envelopes are typically negatively charged at physiological pH, cationic nanoparticle surfaces can bind electrostatically and disrupt membranes more effectively. Positively charged nanomaterials are also known to preferentially adhere to bacterial surfaces over mammalian cells because of the stronger negative potential of bacteria [16]. 

The optical properties of MnO₂@PMS nanospheres were examined by UV–Vis–NIR spectroscopy (Fig. 2F). The nanospheres exhibited a broad absorbance profile across the visible and NIR range, with no sharp peaks, consistent with the dark brown/black color of MnO₂. This absorption tail into the NIR “biological window” is critical for photothermal applications. Upon 808 nm laser irradiation (1.0 W cm^− 2^), MnO₂@PMS dispersions rapidly heated, achieving a ΔT ≈ 30 °C within 10 min (e.g., ~ 25 °C to ~ 55 °C; Fig. 2F). The pronounced temperature rise confirmed efficient photothermal conversion under near-infrared illumination. This transduction was attributed to the MnO₂ shell (a semiconductor with sub-bandgap NIR absorption) and possibly synergistic light-to-heat conversion from surface-bound species. These findings agree with recent studies reporting MnO₂-based nanostructures as effective photothermal agents under NIR exposure [17]. Notably, the achieved temperature (> 50 °C) is sufficient to exert hyperthermic bactericidal effects and also serves as a trigger for catalytic reactions. No significant photothermal degradation occurred over repeated on/off laser cycles (Fig. 2G), demonstrating stable photothermal performance during treatment.

Fourier-transform infrared (FTIR) spectroscopy (Fig. 2H) further confirmed PMS integration. The MnO₂@PMS spectrum displayed new vibrational bands near ~ 1100 cm⁻¹ (strong) and ~ 880 cm⁻¹, absent in bare MnO₂. These correspond to S = O stretching and peroxo O–O vibrations of the peroxymonosulfate anion, respectively, confirming PMS chemical incorporation within the nanospheres. FTIR results, combined with EDS, demonstrated successful PMS loading and preservation of characteristic functional groups inside the hollow framework.

To verify the generation of reactive species, MnO₂@PMS was incubated with 3,3’,5,5’-tetramethylbenzidine (TMB) and the absorbance spectrum was recorded. A distinct peak centered at ~ 650 nm was observed, which increased significantly compared to the control, indicating oxidative activity (Fig. 2J). Radical quenching assays further demonstrated that the oxidative signal was attenuated by isopropanol and ethanol, confirming the involvement of hydroxyl and sulfate species. To further compare the kinetics of PMS- and H₂O₂-mediated oxidation, TMB absorbance was monitored at 0, 10, and 30 min (Fig. 2K). PMS exhibited a faster and higher absorbance increase than H₂O₂, reaching nearly 0.4 a.u. at 30 min versus ~ 0.3 a.u. for H₂O₂, highlighting the efficient activation of PMS by MnO₂ under NIR irradiation. These findings directly support the generation of sulfate radicals consistent with transition-metal-mediated PMS activation [14]. The MnO₂ shell likely contributed via two complementary roles:

(i) Catalytic role–Mn in multiple oxidation states (Mn(IV)/Mn(III)) can mediate one-electron transfer with HSO₅⁻, producing SO₄•⁻ in a Fenton-like process [18].
(ii) Photothermal role–localized hyperthermia from the MnO₂ shell accelerated PMS decomposition kinetics, as elevated temperature favors radical formation [19–21].

Beyond ROS generation, our platform was designed to exploit the self-decomposition of MnO₂ under NIR stimulation to release functional metal ions. Inductively coupled plasma analysis quantified manganese (Mn²⁺) and potassium (K⁺) release from nanospheres with and without NIR irradiation (Fig. 2L, M). In the absence of laser exposure, MnO₂@PMS particles remained stable in neutral aqueous media, releasing negligible Mn²⁺ or K⁺ over several hours. By contrast, upon 808 nm laser irradiation, a pronounced increase in both Mn²⁺ and K⁺ concentrations was observed. Within 10 min, [Mn²⁺] rose to ~ 25 µM, while virtually no Mn²⁺ was detected in dark controls. A corresponding increase in [K⁺] confirmed consumption and release of core-loaded KHSO₅. We attribute this to photothermal decomposition of the nanoplatform: local heating and radical chemistry partially degraded the MnO₂ shell, releasing Mn²⁺ ions, while simultaneously breaking down PMS into soluble products (K⁺, sulfate, etc.). This light-responsive disassembly was deliberately engineered to transform inert MnO₂ into bioactive Mn²⁺ at infection sites. The released Mn²⁺ synergizes with other processes in several ways. First, Mn²⁺ functions as a Fenton-like catalyst in the presence of physiological H₂O₂ (commonly produced by activated immune cells at infection sites), generating additional hydroxyl radicals (•OH) that attack bacteria [22, 23]. This chemo-dynamic effect, analogous to Mn-based chemodynamic therapy in oncology, provides a secondary ROS pathway beyond sulfate radicals. Second, Mn²⁺ perturbs bacterial metabolism and has been reported to inhibit specific bacterial enzymes at elevated concentrations [24, 25]. Finally, K⁺ release, although mainly reflecting PMS consumption, may also impose osmotic stress on bacteria in confined biofilm environments.

Crucially, all these effects, photothermal heating, sulfate radical oxidation, and metal-ion release, occur simultaneously and on demand under the single stimulus of NIR light(Fig. 2N). This trifunctional mode of action is highly advantageous against resilient infections such as biofilms. Biofilm matrices impede drug penetration and shield bacteria, but localized hyperthermia weakens the structure and enhances diffusion of ROS and ions. Meanwhile, ROS (SO₄•–, •OH) attack bacteria both extracellularly and intracellularly, while released Mn²⁺ ions sustain oxidative stress or exert direct toxicity even after laser exposure ceases. This multi-pronged attack is difficult for bacteria to resist, and importantly, the platform is activated only in infected areas by applied NIR—minimizing systemic side effects.

This design philosophy aligns with emerging synergistic nanotherapies for bacterial eradication. For example, Yang et al. recently reported hollow Cu₂MoS₄ nanospheres functioning as “smart nanozymes” that combine photothermal ROS generation with enzyme-mimic catalytic activity to self-adaptively destroy biofilms [26]. In that system, the hollow nanostructure enabled encapsulation of reactants and responsiveness to the infection microenvironment, paralleling our hollow MnO₂ shell that confines PMS and unleashes its activity under stimulus. More broadly, our results demonstrate that integrating photothermal conversion, catalytic PMS activation, and matrix degradation into a single nanoplatform yields amplified bactericidal efficacy.

The release of Mn²⁺ (a product of nanoplatform biodegradation) also highlights its benign degradability: after performing its function, MnO₂ is reduced to soluble Mn²⁺, which can be processed and excreted by normal physiological pathways [27]. Overall, this NIR-responsive MnO₂@PMS hollow nanosystem functions as a promising multimodal strategy in this model for combating biofilm-associated infections by combining localized heating, oxidative radical generation (SO₄•– and •OH), and therapeutic ion release in the absence of adjunct antibiotics. In our experimental setting, this cooperative mechanism enhanced biofilm disruption and bacterial killing, including against drug-resistant strains, and may complement existing nanozyme and photothermal approaches.

### Destruction of biofilm structural barrier via MnO₂@PMS-mediated synergistic activation

**Fig. 3:**
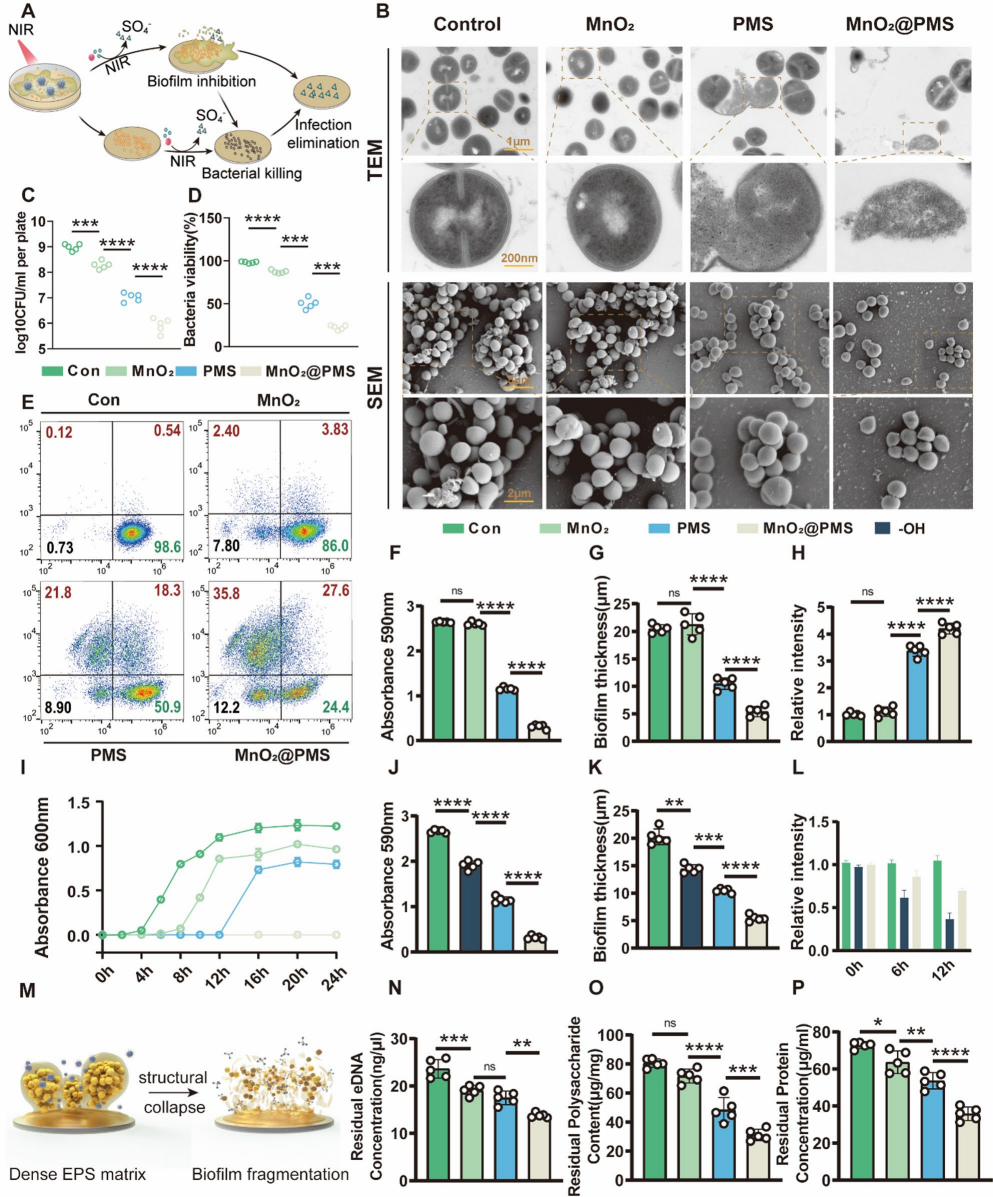
NIR-Triggered Biofilm Eradication via MnO₂@PMS-Mediated Synergistic Hyperthermia and •SO₄⁻ Generation. **(A)** Schematic illustration of the NIR-triggered antibacterial mechanism, involving bacterial adsorption, sulfate radical (•SO₄⁻)-mediated bacterial killing, biofilm disruption, and infection clearance. **(B)** Transmission electron microscopy (TEM) images of *S. aureus* and scanning electron microscopy (SEM) images of corresponding biofilms after treatment with PBS, MnO₂, PMS, or MnO₂@PMS, followed by 808 nm laser irradiation. Scale bars: 1 μm, 200 nm (TEM); 5 μm, 2 μm (SEM). **(C)** Quantification of viable bacteria by colony-forming unit (CFU) assay after treatment. **D–E.** Flow cytometry plots of SYTO9/PI-stained *S. aureus* post-treatment, showing bacterial membrane integrity and viability changes. **F–G.** Quantification of biofilm biomass by crystal violet staining (F) and biofilm thickness by confocal z-stack analysis (G) following PT–PS treatment. **H.** Intracellular reactive oxygen species (ROS) levels in biofilms after various treatments. **I.** Time-dependent bacterial growth curves following treatment, indicating inhibition kinetics over 24 h. **J–K.** Quantification of biofilm biomass and thickness under treatment with H₂O₂, PMS, or MnO₂@PMS plus 660 nm NIR irradiation. **L.** ROS-mediated degradation profiles in biofilms treated with H₂O₂ or MnO₂@PMS, reflecting differences in oxidative species dynamics. **M.** Schematic illustration of biofilms before and after treatment, showing degradation of the extracellular matrix and dispersion of structural components such as extracellular DNA (eDNA). **N–P.** Quantitative analysis of residual extracellular DNA (eDNA, N), polysaccharides (O), and proteins (P) in biofilms after different treatments, reflecting matrix disruption efficiency. Data are presented as mean ± SD (*n* = 3–5). Statistical significance was assessed by one-way ANOVA with Tukey’s post hoc test: *P < 0.05, **P < 0.01, ***P < 0.001, ****P < 0.0001

To overcome the intrinsic resistance of *Staphylococcus aureus* biofilms to conventional therapies, we evaluated the ability of MnO₂@PMS nanoparticles (NPs) to eradicate methicillin-resistant *S. aureus* (MRSA) and disrupt biofilm structures under near-infrared (NIR) irradiation. MRSA biofilms remain a formidable therapeutic obstacle because of their dense EPS, metabolic heterogeneity, and hypoxic core, which collectively impair antibiotic penetration and immune clearance. To address these barriers, we exploited the photothermal and catalytic properties of MnO₂ in combination with peroxymonosulfate (PMS), enabling a dual-action strategy that integrates heat-induced membrane destabilization with oxygen-independent oxidative stress **(**Fig. 3A**)**. To dissect the contribution of each component and activation condition, we designed a panel of mechanistically relevant control groups including PBS, MnO₂-only, PMS-only, and MnO₂@PMS with or without 808 nm near-infrared (NIR) irradiation (Figs. 3 and S6–S14).

Exposure to an 808 nm NIR laser triggered marked ultrastructural damage in both planktonic MRSA and the surrounding biofilm matrix, as revealed by TEM and SEM. Specifically, MnO₂@PMS + NIR-treated bacteria exhibited ruptured membranes, vacuolized intracellular compartments, and collapsed EPS networks, characteristic of combined thermal and oxidative lysis (Fig. 3B). By contrast, bacteria in control groups, including MnO₂+NIR, PMS + NIR, and MnO₂@PMS without irradiation, retained intact membranes and well-preserved biofilm architecture, underscoring the necessity of synergistic activation (Figure S13–S14, *Supporting Information*).

This disruption of structural integrity translated into potent bactericidal activity. Colony-forming unit (CFU) assays demonstrated a ~ 4-log reduction in viable MRSA in the MnO₂@PMS + NIR group compared with PBS, and ~ 2–3 log reductions relative to single-component controls (Fig. 3C). These findings were corroborated by supplementary experiments showing consistent trends across biological replicates (Figure S4–S5, *Supporting Information*). Furthermore, SYTO9/PI dual staining with fluorescence microscopy revealed the highest proportion of PI-positive (dead) bacteria in the MnO₂@PMS + NIR group, with prominent red fluorescence signals indicative of widespread membrane compromise (Fig. 3D–E, Figure S8–S9, *Supporting Information*). Flow cytometry analysis revealed a clear increase in PI⁺ bacterial subpopulations in the MnO₂@PMS + NIR group, reaching approximately 28% in the upper-right quadrant, compared with ~ 18% in the single-component treatment groups and < 1% in the PBS control. These results, together with the low percentage of PI⁺ cells in untreated or partially treated samples, highlight the enhanced bactericidal effect achieved only through combined photothermal–PMS activation.

Real-time OD_600_ monitoring further confirmed that MnO₂@PMS + NIR treatment almost completely suppressed MRSA proliferation over a 24-h period (Fig. 3I). Growth curves of MnO₂-only or PMS-only groups exhibited delayed but ultimately exponential recovery, while untreated controls displayed classic sigmoidal kinetics. Collectively, these data demonstrate that the MnO₂@PMS + NIR system not only induces immediate bactericidal activity but also prevents long-term regrowth, an essential consideration for mitigating biofilm recurrence.

Mechanistically, the antibacterial effects were attributed to ROS generation, particularly sulfate radicals (•SO₄⁻), catalyzed by MnO₂ under photothermal stimulation. Quantitative ROS assays revealed a pronounced oxidative burst in the MnO₂@PMS + NIR group, with signal intensities exceeding those of MnO₂ or PMS alone (Fig. 3H). Notably, substrate degradation assays indicated that the MnO₂@PMS nanoplatform maintained oxidative activity for a longer duration within biofilm matrices compared with H₂O₂-treated groups, as reflected by a slower but more sustained decline in signal intensity over 12 h (Fig. 3L). These findings align with reports that sulfate radicals possess superior oxidative potential and longer diffusion ranges than hydroxyl radicals (•OH), particularly in viscous or biologically complex environments [28]. Because MnO₂ mediates PMS cleavage independent of oxygen, this approach is especially advantageous for targeting hypoxic biofilm cores [29]. 

To assess biofilm structural disruption, we performed matrix profiling. Crystal violet staining revealed a significant reduction in total biomass in the MnO₂@PMS + NIR group (Fig. 3F), with over 60% reduction relative to PBS and 40–50% decreases compared with single-agent controls. Confocal laser scanning microscopy (CLSM) of SYTO9/TOTO-3–stained biofilms showed extensive collapse, including fragmented eDNA networks and reduced nucleic acid density in the MnO₂@PMS + NIR group (Figure S10, *Supporting Information*). These results were corroborated by quantitative biofilm thickness analysis (Fig. 3G) and z-stack reconstructions (Figure S11–S12, *Supporting Information*), together indicating ~ 50% reduction in biofilm height.

We further quantified three key extracellular matrix components, extracellular DNA (eDNA), polysaccharides, and total protein to define biochemical degradation of EPS. MnO₂@PMS + NIR treatment significantly reduced all three (Fig. 3N–P), with eDNA showing the greatest susceptibility, likely reflecting its higher oxidative sensitivity. These reductions were absent in MnO₂-only or PMS-only groups, underscoring the importance of ROS intensity and localization. Notably, similar EPS degradation trends have been observed with α-amylase-assisted MnO₂ platforms and Janus-type hollow photothermal particles [30, 31]. 

Together, these findings establish MnO₂@PMS + NIR as a robust strategy for dismantling biofilm architecture through dual photothermal and chemical mechanisms. This nanoplatform achieves concurrent bacterial inactivation and EPS disassembly, targeting both cellular and structural determinants of biofilm persistence. These outcomes lay the foundation for subsequent exploration of downstream immune modulation and tissue repair.

### Glutamate metabolic disruption as a structural and immunoregulatory node in biofilm breakdown

**Fig. 4:**
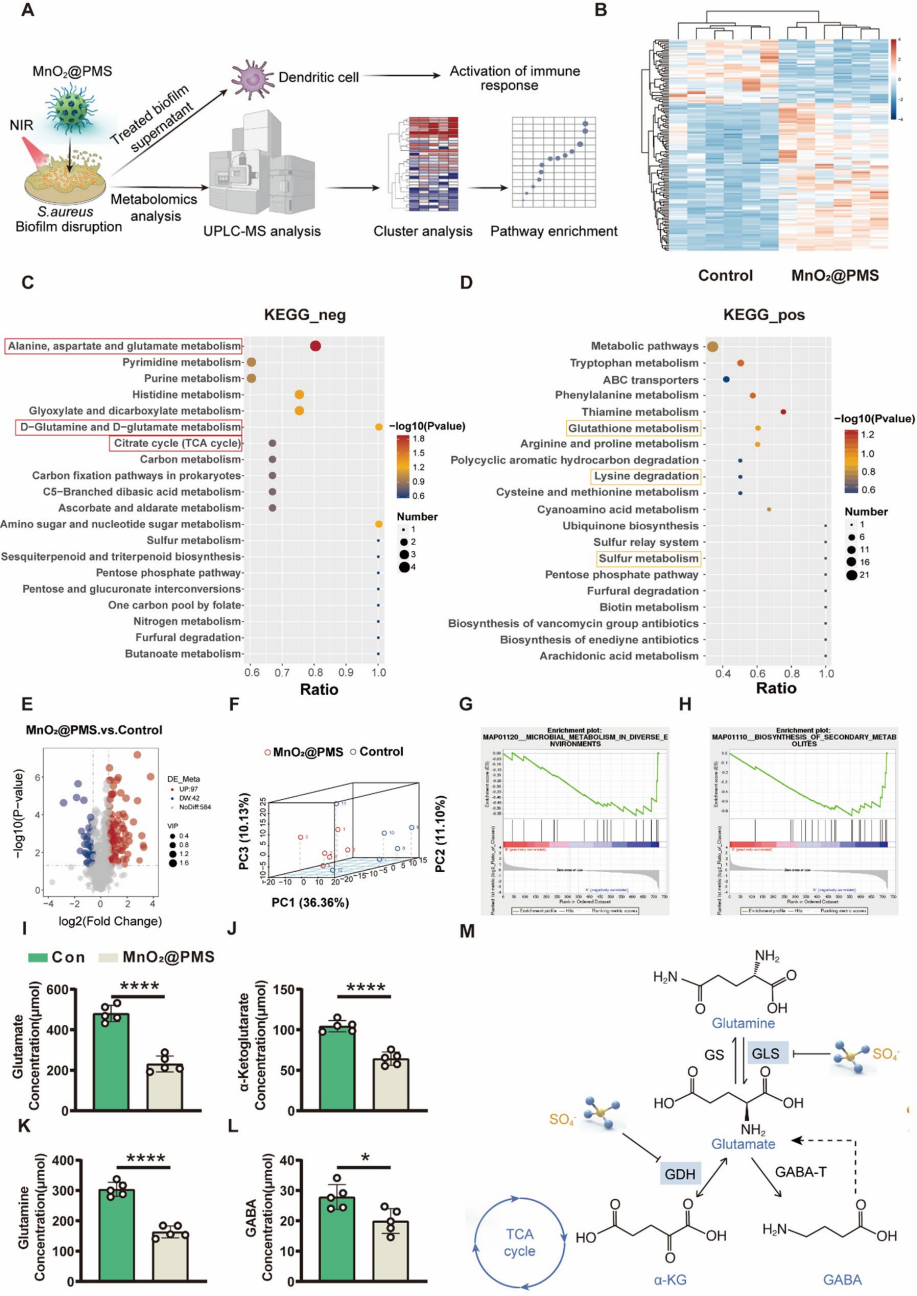
Metabolomic and biochemical profiling of *S. aureus* biofilms treated with MnO₂@PMS. **(A)** Schematic workflow of untargeted metabolomics on biofilm supernatants post MnO₂@PMS-mediated photothermal–photosensitizer (PT–PS) disruption. The pipeline includes NIR-triggered treatment, metabolite extraction, UPLC-MS analysis, clustering, and KEGG pathway enrichment to explore metabolic shifts potentially influencing dendritic cells activation. **(B)** Heatmap illustrating differential metabolite expression between MnO₂@PMS-treated and control groups. **(C)** KEGG-based enrichment of downregulated pathways in the treated group (top 20), highlighting suppression of glutamate-associated networks. **(D)** KEGG-based enrichment of upregulated pathways in the treated group (top 20), indicating activation of compensatory metabolic routes. **(E)** Volcano plot displaying significantly altered metabolites (red for upregulated, blue for downregulated). **(F)** Principal component analysis (3D PCA) showing distinct metabolic profiles and group separation. **G–H.** Gene set enrichment analysis (GSEA) of microbial metabolic pathways, emphasizing the global metabolic reprogramming in response to MnO₂@PMS. **I**–**L**. Biochemical validation of key glutamate metabolism–related intermediates in biofilm supernatants using quantitative enzymatic assay kits: I. Glutamate concentration in biofilm supernatants; J. α-ketoglutarate levels as a tricarboxylic acid (TCA) cycle indicator; K. Glutamine levels reflecting glutamate–glutamine cycling; L. γ-aminobutyric acid (GABA) as a downstream product of glutamate catabolism. **M.** Proposed schematic of sulfate radical (•SO₄⁻)-mediated inhibition of glutamate metabolism. MnO₂@PMS-generated •SO₄⁻ radicals oxidatively interfere with glutaminase (GLS) and glutamate dehydrogenase (GDH), key enzymes involved in glutamate biosynthesis and catabolism. Data are presented as mean ± SD (*n* = 3–5). Statistical analysis was performed using one-way ANOVA (I–L); *P < 0.05, **P < 0.01, ***P < 0.001, ****P < 0.0001

Building on the observed collapse of the biofilm matrix, we investigated how MnO₂@PMS reshapes the metabolic landscape of S. aureus biofilms. (Fig. 4A). Hierarchical clustering revealed marked divergence in metabolite expression between MnO₂@PMS-treated and control groups, indicating broad metabolic reprogramming (Fig. 4B). Kyoto Encyclopedia of Genes and Genomes (KEGG) pathway enrichment of downregulated metabolites demonstrated significant suppression of central energy-generating and biosynthetic pathways, with strong inhibition in glutamate metabolism (*p* < 0.001), the citrate (TCA) cycle (*p* < 0.01), and carbon metabolism (*p* < 0.05) (Fig. 4C). These changes signify a collapse in core metabolic capacity following ROS stress, compromising bacterial viability and the ability to sustain an organized biofilm structure. In contrast, upregulated pathways primarily involved sulfur metabolism (*p* < 0.05), glutathione biosynthesis (*p* < 0.01), and lysine degradation (*p* < 0.05) (Fig. 4D).

Volcano plots illustrated significant differential expression of key metabolites (*p* < 0.05) (Fig. 4E), and gene set enrichment analysis (GSEA) revealed coordinated downregulation of pathways linked to microbial adaptability, including environmental metabolism (*p* < 0.05) and secondary metabolite biosynthesis (*p* < 0.05) (Fig. 4G–H). These suppressed pathways are central to antioxidative defense and interspecies communication in biofilms, suggesting that MnO₂@PMS compromises not only structural stability but also biochemical resilience. Comparable phenomena have been reported in fungal systems, where gliotoxin enhances biofilm survival and immune evasion by modulating host responses [24]. 

Among the disrupted pathways, glutamate metabolism emerged as a central target of MnO₂@PMS-mediated metabolic reprogramming. As a pivotal amino acid, glutamate serves as a precursor for poly-γ-glutamic acid (PGA), an extracellular polymer that reinforces biofilm adhesion, promotes immune evasion, and enhances resistance to phagocytosis. Previous studies identified PGA as a major virulence factor in *Staphylococcus epidermidis* biofilms, essential for persistence within hostile host environments [32]. Beyond its structural role, glutamate metabolism may also contribute to immunomodulation. For instance, in neonatal rat spinal cord astrocytes, glutamate significantly potentiated LPS-stimulated IL-10 release and mRNA expression, suggesting its role in immune regulation [33]. These observations position glutamate metabolism as a potential metabolic–immune interface. Accordingly, suppression of glutamate metabolism in biofilms may not only degrade extracellular matrix integrity but could also attenuate immunosuppressive signaling, such as IL-10–mediated pathways, although this remains to be directly validated in bacterial systems.

Targeted metabolomic profiling of biofilm supernatants revealed that MnO₂@PMS treatment significantly perturbed the glutamate-centred metabolic axis. To corroborate these LC–MS/MS findings, we performed biochemical quantification of glutamate and related intermediates in the same biofilm supernatants using commercial enzymatic assay kits. These assays demonstrated marked decreases in the absolute concentrations of several key metabolites, including glutamate (↓ 41.3%, *p* < 0.0001), α-ketoglutarate (↓ 38.7%, *p* < 0.0001), glutamine (↓ 34.5%, *p* < 0.0001), and GABA (↓ 17.6%, *p* = 0.015) compared with PBS-treated biofilms (Fig. 4I–L). These reductions were consistent with the pathway enrichment results (Fig. 4C), which highlighted alanine, aspartate, and glutamate metabolism and the TCA cycle as primary affected routes. Mechanistically, these findings suggest that sulfate radicals generated by MnO₂@PMS may oxidatively target key enzymes such as glutaminase (GLS) and glutamate dehydrogenase (GDH), thereby restricting flux through biosynthetic and redox pathways. This inhibitory mechanism is illustrated schematically in Fig. 4M.

This concept of metabolically reprogramming biofilms to weaken immune evasion is consistent with a broader trend in anti-infective strategies that draw on immunometabolic interventions, one that parallels immunometabolic interventions in cancer and chronic inflammatory disease. For example, *S. aureus* biofilms release metabolites that suppress pro-inflammatory cytokine production and promote regulatory phenotypes in myeloid cells [5]. Similarly, in tumor microenvironments, altered amino acid metabolism and nutrient competition reshape immune responses, driving disease progression [34]. These parallels highlight the therapeutic potential of metabolic rewiring in chronic infections.

Metabolic suppression also translates into altered host responses. For instance, lactate accumulation in *S. aureus* biofilms suppresses histone deacetylase 11 (HDAC11) activity in host immune cells, thereby modifying chromatin accessibility and gene expression to promote immune tolerance [5]. In this context, by disrupting glutamate metabolism, MnO₂@PMS may likewise modulate host–pathogen interactions through metabolite depletion and reduced immunosuppressive signaling.

Collectively, these findings point to glutamate metabolism as one important structural and immunoregulatory node that contributes to biofilm resilience and DC tolerance, likely acting together with other metabolic and redox pathways. MnO₂@PMS-mediated suppression of this pathway compromises matrix biosynthesis while simultaneously weakening biofilm-driven immune escape. This dual-targeting mechanism is consistent with a mechanistically grounded strategy for biofilm-focused metabolic immunotherapy in this model. Guided by these insights and the recognized immunomodulatory roles of glutamate, we next investigated the impact on dendritic cells and subsequent adaptive immune responses.

### Glutamate-mediated immunosuppression within biofilms and its reversal by MnO₂@PMS

**Fig. 5:**
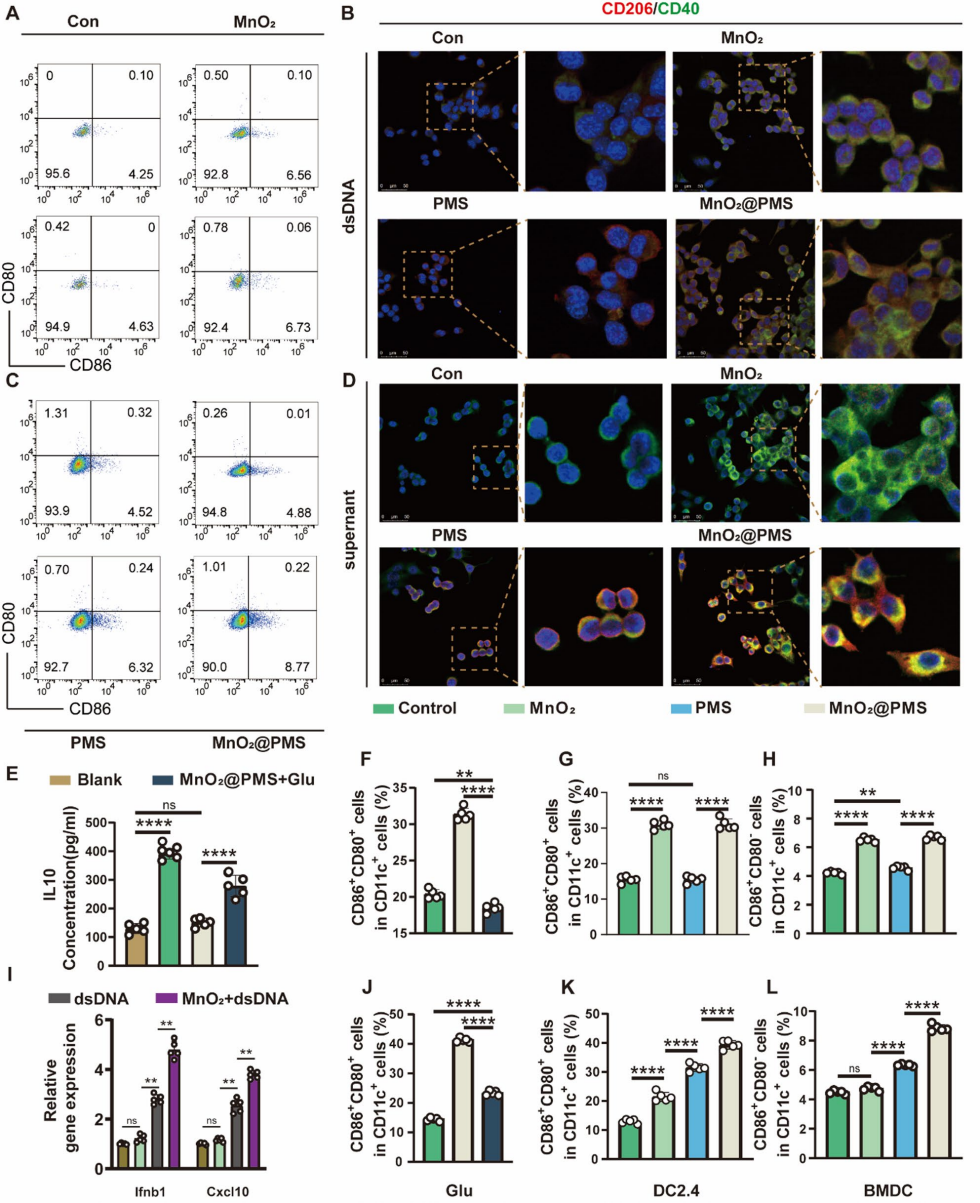
Evaluation of dendritic cells activation and immunomodulation upon MnO₂@PMS treatment. **A**,** C.** Flow cytometry analysis of CD80⁺CD86⁺ primary BMDCs stimulated with dsDNA (**A**) or biofilm-derived supernatant (**C**) under different treatment conditions (Control, MnO₂, PMS, MnO₂@PMS). **B**,** D.** Representative immunofluorescence images of DC2.4 cells under the same stimulations (B: dsDNA; D: biofilm supernatant), stained for nuclei (blue), CD206 (green), and CD40 (red). MnO₂@PMS enhances dendritic cells activation and morphological transformation. **E.** ELISA quantification of IL-10 levels in DC2.4 supernatants. MnO₂@PMS significantly suppresses IL-10 secretion induced by biofilm supernatant, while glutamate (Glu) rescue partially restores it. **F–H.** Proportion of CD80⁺CD86⁺ and CD80⁻CD86⁺ subsets in response to dsDNA stimulation in Glu-treated DC2.4 cells (**F**), untreated DC2.4 cells (**G**), and primary BMDCs (**H**). RT–qPCR analysis of Ifnb1 and Cxcl10 in DC2.4 cells treated with MnO₂@PMS alone, dsDNA alone, or MnO₂@PMS plus dsDNA, showing MnO₂@PMS-dependent amplification of dsDNA-induced gene expression. **J–L.** Proportions of CD80⁺CD86⁺ and CD80⁻CD86⁺ DCs after biofilm supernatant stimulation, validating immune activation rescue by MnO₂@PMS in Glu-treated DC2.4 cells (**J**), untreated DC2.4 cells (**K**), and primary BMDCs (**L**). Data are presented as mean ± SD. Statistical significance was evaluated by one-way ANOVA (**F–H, J–L, M**); *P < 0.05, **P < 0.01, ***P < 0.001, ****P < 0.0001

Guided by our metabolomic findings implicating glutamate metabolism as a metabolic–immune checkpoint in *Staphylococcus aureus* biofilms, we next examined its downstream immunological consequences in DCs. To probe the specificity of the nanoplatform components, DC2.4 cells and bone marrow–derived dendritic cells (BMDCs) were stimulated with conditioned media derived from biofilms treated with PBS, MnO₂-only, PMS-only, or MnO₂@PMS under identical NIR irradiation conditions (Figs. 5 and S19–S21). Although direct causality between glutamate and IL-10 production in DCs remains underexplored, prior studies indicate that glutamate can regulate cytokine secretion and promote tolerogenic phenotypes in multiple immune settings [35, 36]. Importantly, glutamate plays a dual role in the biofilm niche, serving both as a structural precursor (e.g., poly-γ-glutamic acid) and as a potential immunomodulatory metabolite, positioning it as a critical metabolic node at the interface of bacterial persistence and host immune evasion. We therefore investigated whether glutamate contributes to the immunosuppressive microenvironment of biofilms and whether these effects can be reversed by MnO₂@PMS through modulation of DCs activation and polarization.

Bacterial double-stranded DNA (dsDNA) is a potent pathogen-associated molecular pattern capable of activating DCs through cytosolic DNA-sensing pathways such as cGAS–STING. In chronic biofilm infections, immunological inertia has been attributed either to insufficient dsDNA release or impaired host recognition. However, our earlier results confirmed that dsDNA is abundant within the biofilm matrix, suggesting that its immune-stimulatory potential is functionally silenced by the surrounding microenvironment. To dissect the influence of biofilm-derived suppressive factors on DNA-sensing pathways, we stimulated dendritic cells with purified dsDNA and compared their responses to those elicited by biofilm supernatants, which naturally contain dsDNA along with other metabolites.This approach allowed us to assess how immunosuppressive metabolites influence canonical activation pathways.

Under classical immune-stimulatory conditions using bacterial dsDNA, MnO₂@PMS markedly enhanced DCs activation. Flow cytometry revealed elevated CD80⁺CD86⁺ double-positive populations in both DC2.4 and bone marrow-derived DCs (Fig. 5A and F–H; Figure S20, *Supporting Information***)**. Immunofluorescence imaging further demonstrated increased CD40 expression and morphological features consistent with mature DCs in the MnO₂@PMS group (Fig. 5B). Supporting analyses (Figure S15 and Figure S18) likewise confirmed that MnO₂-based nanocomposites promote robust DCs maturation in response to dsDNA, independent of soluble factors derived from biofilms. These results validate the immune-activating capacity of MnO₂@PMS under pathogen-mimicking conditions. To further delineate the requirement for dsDNA in this response, we quantified the expression of downstream cGAS–STING target genes (Ifnb1 and Cxcl10) in DCs treated with MnO₂@PMS in the presence or absence of exogenous bacterial dsDNA. MnO₂@PMS alone induced only minimal changes in Ifnb1 and Cxcl10 transcripts, whereas dsDNA alone elicited a moderate increase, and the combination of MnO₂@PMS with dsDNA produced the highest induction (Fig. 5I). These data indicate that Mn²⁺ released from MnO₂@PMS acts as a cofactor that amplifies dsDNA-triggered cGAS–STING activation rather than serving as an independent STING agonist.

When DCs were exposed to *S. aureus* biofilm supernatants, rich in secreted glutamate and other immunosuppressive metabolites, DCs maturation was markedly suppressed across all groups. Remarkably, MnO₂@PMS retained its ability to restore the CD80⁺CD86⁺ phenotype even within this suppressive context (Fig. 5C–D and J–L; Figure S19, Figure S21, *Supporting Information*). This restorative effect was abrogated by exogenous glutamate supplementation, further confirming glutamate’s central role in mediating biofilm-induced immune tolerance. Quantitative enzyme-linked immunosorbent assay (ELISA) corroborated these results, showing that MnO₂@PMS significantly reduced IL-10 levels in culture supernatants, which were otherwise elevated by biofilm exposure and reinstated by glutamate rescue (Fig. 5E). Together, these findings support the concept that glutamate functions as an immunosuppressive metabolite capable of regulating IL-10 production and antigen presentation in DCs, as suggested by studies in other immune contexts [37]. Gene expression profiling further substantiated this mechanistic link. MnO₂@PMS treatment upregulated CD40 and H-2D, genes critical for antigen presentation and T-cell activation, while also increasing CD206 and Timd4, typically associated with tolerogenic DCs phenotypes (Figure S25, Supporting Information). This mixed transcriptional profile may represent a transitional state rather than a fully polarized phenotype. Under these biofilm-conditioned settings, supernatants from PBS-, MnO₂-, or PMS-treated biofilms induced only partial restoration of CD80⁺CD86⁺ DCs and modest changes in cytokine secretion, whereas MnO₂@PMS-conditioned media produced the most pronounced recovery of DC maturation and pro-inflammatory cytokine production (Figs. 5 and S19–S21).

Mechanistically, glutamate-derived immunosuppression may be mediated through enzymatic targets vulnerable to radical oxidation. Sulfate radicals generated by MnO₂@PMS are capable of inducing oxidative modifications in key enzymes of glutamate metabolism, such as glutaminase (GLS) and glutamate dehydrogenase (GDH). Such modifications can impair enzymatic activity, lowering intracellular glutamate production and restricting its immunomodulatory spillover into the extracellular space. Supporting this mechanism, prior studies have shown that radical-induced carbonylation and nitration can inactivate GDH, attenuating glutamate flux and downstream GABA synthesis [38]. In agreement, our metabolomic and biochemical validations (Fig. 4I–L and M) demonstrated significant reductions in glutamate and its associated metabolites following MnO₂@PMS exposure. In chronic infection, biofilm-derived glutamate not only sustains matrix cohesion through poly-γ-glutamic acid (PGA) production but also functions as a local immune suppressor, dampening inflammatory responses and promoting IL-10–rich tolerogenic niches. These findings parallel tumor immunometabolism, where nutrient depletion and metabolite accumulation drive immune anergy and skew cytokine profiles [34]. 

Our results align with recent studies showing that *S. aureus* biofilms secrete lactate and other small-molecule metabolites capable of suppressing HDAC11 in host cells, thereby altering chromatin accessibility and transcriptional programming in DCs and macrophages [5]. Within this framework, MnO₂@PMS-mediated glutamate depletion performs a dual role, weakening biofilm structural integrity while simultaneously disarming its immunomodulatory arsenal.

Collectively, these findings delineate a two-tiered mechanism of immune modulation in biofilm-infected niches: (1) metabolic suppression via glutamate accumulation leading to IL-10 upregulation, and (2) altered DCs gene expression profiles reflecting an imbalance between immunostimulatory and tolerogenic programs. MnO₂@PMS interrupts this immunosuppressive axis by depleting extracellular glutamate and enhancing the expression of genes governing antigen presentation and T-cell activation. This immunological restoration complements its biofilm-disruptive and metabolic-rewiring effects, establishing MnO₂@PMS as a promising therapeutic approach against biofilm-associated immune evasion.

### Multi-omics reveal MnO₂@PMS reverses biofilm-induced dendritic cells tolerization via cGAS–STING pathway reactivation

**Fig. 6:**
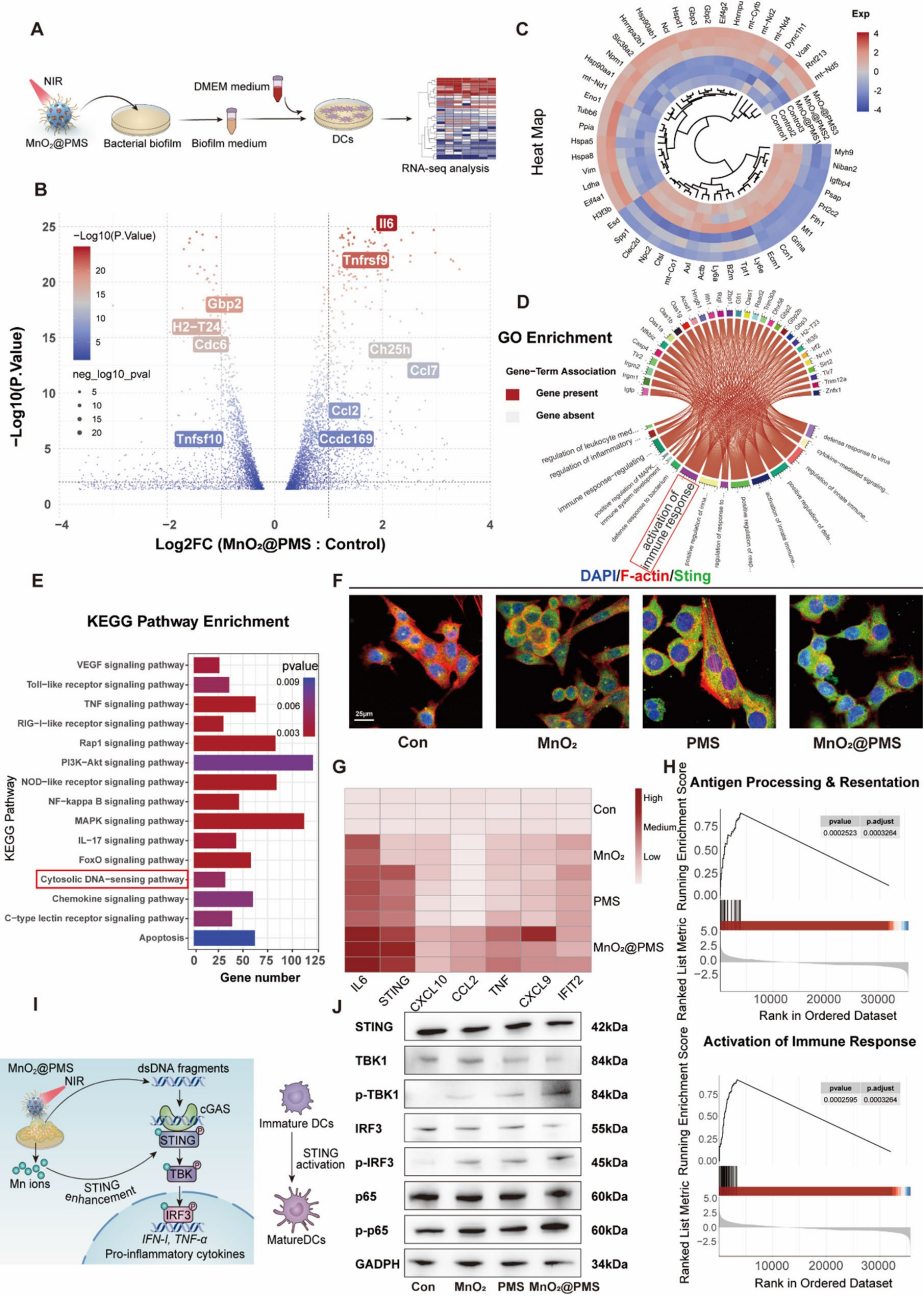
Transcriptomic and molecular validation of cGAS–STING pathway activation in dendritic cells following MnO₂@PMS treatment. **(A)** Schematic illustration of the experimental workflow: biofilm-derived supernatants post MnO₂@PMS + NIR treatment were applied to DCs for transcriptomic and molecular analysis. **(B)** Volcano plot depicting differentially expressed genes (DEGs) between MnO₂@PMS-treated and control DCs. Genes with |log₂FC| > 1 and *p* < 0.05 were considered significant. **(C)** Circular hierarchical heatmap showing representative immune-related DEGs, illustrating transcriptional reprogramming upon MnO₂@PMS stimulation. **(D)** GO enrichment chord diagram highlighting immune-related biological processes enriched among DEGs, including cytokine response, leukocyte activation, and antigen presentation. **(E)** KEGG pathway analysis identifying significantly enriched pathways involved in innate immunity and inflammation. Notably, the cytosolic DNA-sensing pathway is activated (highlighted in red). **(F)** Immunofluorescence staining of STING (green), F-actin (red), and nuclei (blue) in DCs across groups. MnO₂@PMS induces marked STING aggregation and cytoskeletal rearrangement. Scale bar = 25 μm. **(G)** qPCR analysis of key STING-related immune genes (e.g., *Ifnb*, *Ccl2*, *Cxcl10*, *Irf7*), showing upregulation in MnO₂@PMS-treated DCs. **(H)** GSEA enrichment plots showing significant activation of antigen processing and immune response pathways in MnO₂@PMS-treated DCs. **(I)** Proposed mechanism: MnO₂@PMS triggers cGAS–STING pathway activation via synergistic effects of sulfate radicals, Mn²⁺ ion release, and biofilm-derived dsDNA, culminating in pro-inflammatory cytokine secretion and DCs maturation. **(J)** Western blot analysis confirming upregulation and phosphorylation of key proteins in the STING pathway (STING, TBK1, IRF3, p65) in MnO₂@PMS-treated DCs, indicating robust innate immune activation

To delineate the molecular mechanisms underlying DCs reactivation by MnO₂@PMS in biofilm-immunosuppressive conditions, we performed integrated transcriptomic combined with complementary experimental validations. As shown in Fig. 6A, DCs were stimulated with S. aureus biofilm supernatant metabolite-rich milieu containing glutamate and treated with MnO₂@PMS under near-infrared (NIR) irradiation. RNA-seq profiling revealed a pronounced transcriptional shift, with upregulation of pro-inflammatory and immune activation genes, including Il6, Tnfrsf9, and Gbp2, accompanied by downregulation of inhibitory or tolerogenic markers such as Tnfsf10 and Ccl2 (Fig. 6B–C). Gene Ontology enrichment analysis (Fig. 6D) highlighted activation of programs linked to antigen presentation, leukocyte trafficking, and activation of immune response, consistent with reversal of the tolerogenic phenotype.

Pathway-level interrogation by KEGG enrichment (Fig. 6E) and GSEA (Fig. 6H) demonstrated activation of innate immune modules, including Toll-like receptor signaling, RIG-I-like receptor signaling, and most prominently, the cytosolic DNA-sensing pathway. Given our prior demonstration of intact dsDNA within the biofilm matrix, the restoration of DNA sensing primarily through the cGAS–STING axis provides a mechanistic basis for MnO₂@PMS-mediated immune reactivation. The platform’s dual functions, sulfate radical generation and Mn²⁺ release, likely enhance cGAS–STING signaling by inducing oxidative chromatin remodeling and providing essential cofactors (Fig. 6I).

Immunofluorescence confirmed increased STING expression in DCs after treatment (Fig. 6F), while qPCR analysis showed transcriptional upregulation of STING-associated genes (*STING*,* CXCL10*,* IFIT2*) consistent with pathway activation (Fig. 6G). Functionally, ELISA assays of DCs supernatants demonstrated restoration of pro-inflammatory cytokine production, including IL-6, TNF-α, and CXCL10 (Figure S26, *Supporting Information*). Western blot analysis further validated MnO₂@PMS-mediated restoration of TBK1 and IRF3 phosphorylation, core transducers of cGAS–STING signaling, together with activation of NF-κB (Fig. 6J). These findings establish a complete signaling cascade from DNA sensing to transcriptional reprogramming and cytokine release. In parallel, GSEA indicated enhanced expression of genes related to antigen presentation, including MHC-I/II loading and peptide processing (Fig. 6H), confirming that MnO₂@PMS not only restores innate immunity but also primes DCs for adaptive immune orchestration.

Importantly, the transcriptional rewiring of DCs observed here was not solely dependent on canonical pathogen-sensing pathways. Increasing evidence highlights the pivotal role of cellular metabolism in dictating DCs fate and function. In our study, transcriptomic alterations coincided with a metabolic profile favoring immune activation. Consistent with this, prior studies have shown that DCs metabolic reprogramming is closely tied to immunogenic potential, with glycolysis and mitochondrial ROS generation supporting inflammatory cytokine production, whereas glutamine catabolism and fatty acid oxidation promote tolerogenic states [39]. 

Our metabolomic analysis demonstrated that MnO₂@PMS markedly reduced extracellular glutamate levels in the biofilm-conditioned system. Although the precise mechanistic link between glutamate metabolism and DCs function remains to be fully defined, our data indicate that glutamate availability modulates DCs activation. Specifically, MnO₂@PMS treatment was associated with a broad suppression of tolerance-associated features and a concurrent increase in immunogenic molecules such as CD40 and H-2D. Importantly, supplementation with exogenous glutamate partially restored IL-10 expression and attenuated MnO₂@PMS-induced DCs activation, suggesting that antagonizing glutamate metabolism contributes to the observed immunostimulatory effects.

Mechanistically, reactive species generated by MnO₂@PMS, particularly sulfate radicals (•SO₄⁻), may oxidatively inactivate glutamate-synthesizing enzymes, including glutamate dehydrogenase (GDH) and glutaminase (GLS), thereby disrupting the metabolic–immune axis sustaining immunosuppression. Redox proteomic studies have shown that ROS can covalently modify and functionally inhibit GDH and GLS under oxidative stress [38]. Such post-translational modifications, including carbonylation and nitration, compromise enzymatic activity, deplete intracellular glutamate, and disrupt downstream flux. Notably, enzymatic inhibition also limits the biosynthesis of poly-γ-glutamic acid (PGA), a matrix polymer essential for biofilm stability, thereby destabilizing biofilm architecture and reducing its immune evasion capacity.

The immunological implications of this metabolic disruption echo conceptual frameworks in tumor immunology, where nutrient competition and metabolite accumulation foster immune tolerance. In cancer, reprogramming DC metabolism through nutrient limitation or oxidative stress can reinvigorate anti-tumor responses [34]. Analogously, our data are consistent with a model in chronic infection in which MnO₂@PMS functions as a metabolic modulator that alleviates biofilm-induced glutamate accumulation, reduces IL-10 production, and permits reactivation of cGAS–STING signalling, thereby contributing to the restoration of immune competence in concert with other MnO₂@PMS-induced pathways.

Moreover, our findings are consistent with prior reports showing that bacterial metabolites such as lactate can epigenetically silence pro-inflammatory pathways in DCs. For instance, *S. aureus* biofilms produce lactate that inhibits HDAC11 activity, thereby reprogramming DCs toward a suppressive phenotype [40]. This highlights the therapeutic relevance of targeting microbial metabolites in infection-associated immunosuppression. By simultaneously dismantling biofilm structure, suppressing glutamate-driven signalling, and restoring cGAS–STING activation, MnO₂@PMS engages three interrelated processes—structural disruption, metabolic antagonism, and immune restoration—that together mitigate biofilm-mediated tolerance in this model.

In summary, MnO₂@PMS reprograms DCs under biofilm-immunosuppressive stress through a coordinated cascade: (1) it scavenges immunosuppressive metabolites such as glutamate; (2) it reactivates the cGAS–STING pathway via dsDNA sensitization and Mn²⁺ supplementation; and (3) it induces transcriptional and epigenetic remodeling that restores antigen presentation and cytokine production. Collectively, these findings advance the concept of MIT as a robust strategy to counteract chronic infection-associated immune dysfunction. Having delineated this in vitro cascade, we next sought to evaluate whether these immune-restorative effects could be reproduced in vivo using implant-associated infection models. Given the complexity of the biofilm–immune interface, we interpret these data as supporting a model in which glutamate–IL-10 signalling represents one major contributing pathway rather than the sole determinant of DC reactivation.

### MnO₂@PMS exhibits potent in vivo biofilm disruption and infection control with favorable short-term biocompatibility in a murine model

**Fig. 7:**
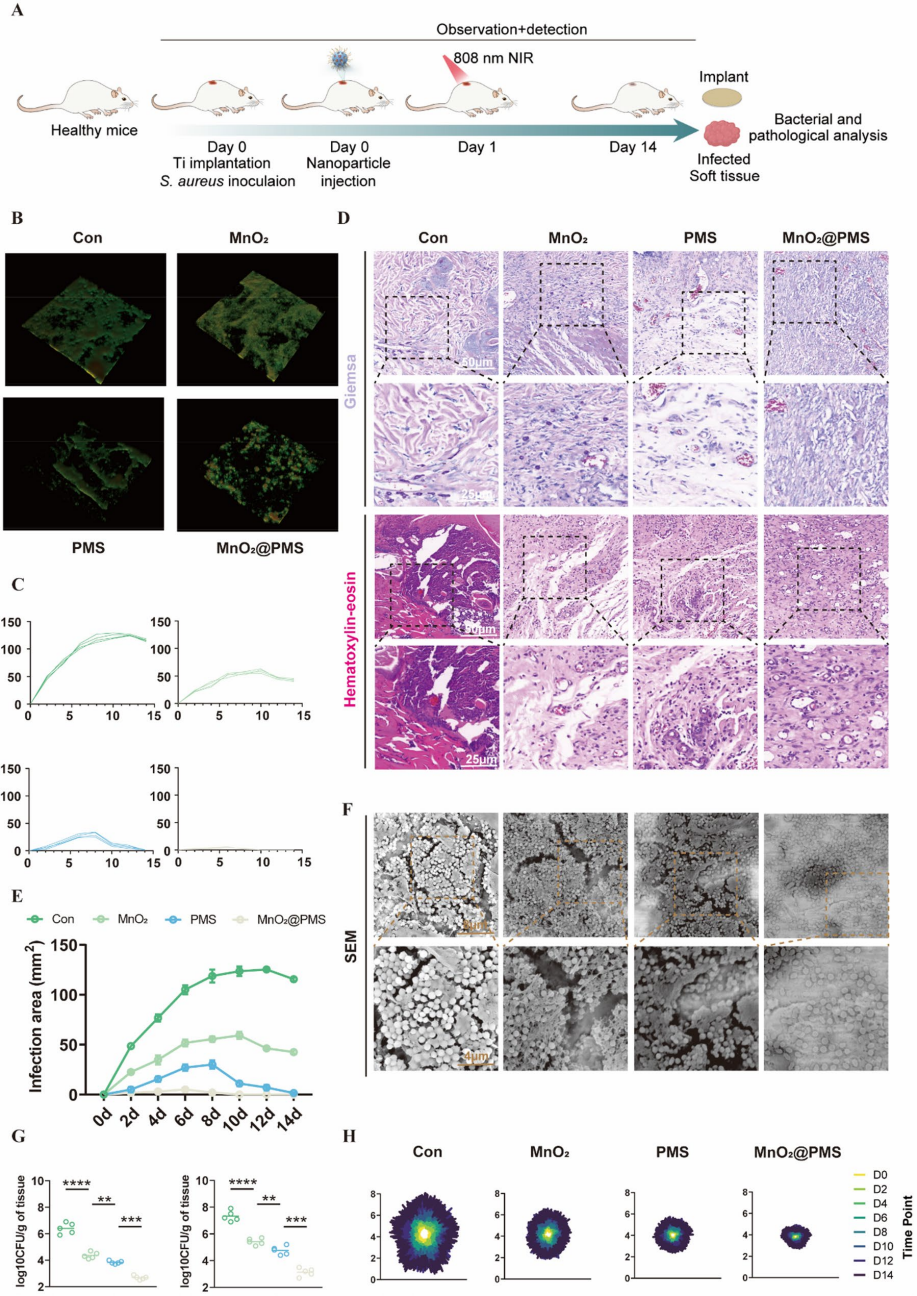
In vivo evaluation of MnO₂@PMS in a murine model of biofilm-associated implant infection. **(A)** Schematic illustration of the experimental workflow. Mice were implanted with Ti rods, followed by *S. aureus* inoculation and immediate injection of therapeutic nanoparticles. 808 nm NIR laser was applied on day 1, and infection resolution was monitored until day 14. **(B)** 3D confocal laser scanning microscopy (CLSM) reconstructions of biofilm viability on implant surfaces stained with SYTO9/PI. MnO₂@PMS markedly increased dead bacterial signals (red) compared to other groups. **(C)** Individual and averaged infection area growth curves in each treatment group across 14 days. **(D)** Representative Giemsa and hematoxylin–eosin (H&E) staining of peri-implant soft tissue sections on day 14. MnO₂@PMS significantly reduces local inflammation and bacterial infiltration. Scale bars: 50 μm (upper), 25 μm (lower). **(E)** Quantification of infection area over time, showing that MnO₂@PMS treatment leads to sustained lesion area reduction (*n* = 5). **(F)** Scanning electron microscopy (SEM) images of explanted Ti rods at day 14 reveal dense biofilm formation in the control group, while MnO₂@PMS treatment results in pronounced biofilm disruption. Scale bars: 8 μm (upper), 4 μm (lower). **(G)** Colony-forming unit (CFU) quantification on explanted implants (top) and peri-implant soft tissues (bottom) at day 14. MnO₂@PMS significantly reduces bacterial burden (*n* = 5). **(H)** Spatiotemporal heatmap tracking of infection area progression in each group from day 0 to day 14. Overlay maps demonstrate the cumulative area and reduction trajectory. Statistical comparisons were performed using one-way ANOVA with Tukey’s post hoc test. Data are expressed as mean ± SD. *P < 0.05, **P < 0.01, ***P < 0.001, ****P < 0.0001

Encouraged by the strong in vitro antibacterial and immunoregulatory effects of MnO₂@PMS, we next evaluated its translational potential in a murine model of implant-associated biofilm infection (Fig. 7A). Mice were randomly assigned to receive local PBS, MnO₂-only, PMS-only, or MnO₂@PMS treatment (*n* = 5 per group), with only the MnO₂@PMS group subjected to 808 nm NIR irradiation, thus providing in vivo counterparts of the component-missing control groups (Figs. 7 and S26–S30). On day 0, sterile titanium implants were introduced subcutaneously, followed by subcutaneous inoculation with a defined concentration of S. aureus suspension, and subsequent nanoparticle administration with 808 nm NIR irradiation. Infection progression and therapeutic outcomes were monitored longitudinally through microbiological and pathological analyses.

SEM imaging of retrieved implants on day 14 revealed dense bacterial colonization and extensive biofilm coverage in controls, whereas MnO₂@PMS treatment resulted in marked biofilm clearance and loss of extracellular matrix (Fig. 7F). CLSM corroborated these findings, showing widespread bacterial death (red) and decreased viable biomass (green) in MnO₂@PMS-treated mice (Fig. 7B), consistent with reduced biofilm thickness and enhanced bactericidal activity (Figure S29, *Supporting Information*).

Histopathological analysis of peri-implant tissues demonstrated severe inflammatory infiltration and tissue destruction in control and single-component groups, while MnO₂@PMS treatment preserved soft tissue architecture and substantially reduced inflammatory cell accumulation (Fig. 7D). Time-course monitoring of infection sites revealed that MnO₂@PMS significantly restricted lesion expansion from day 2 onward, achieving near-complete resolution by day 14 (Fig. 7C, E; Figure S29, *Supporting Information*). Quantitative CFU enumeration on implants and surrounding tissues confirmed significantly lower bacterial burdens in the MnO₂@PMS group than in MnO₂, PMS, or PBS controls (Fig. 7G).

Importantly, H&E staining of major organs showed no histological abnormalities across treatment groups (Figure S26, *Supporting Information*), supporting a favourable short-term systemic safety profile in this model. Macroscopic evaluation further demonstrated reduced swelling and erythema by day 6 in the MnO₂@PMS group, with near-complete tissue recovery by day 14 (Figure S27, Supporting Information). These in vivo experiments were designed as exploratory, and the sample size was not based on a formal power calculation; accordingly, the effect sizes observed here should be interpreted with this limitation in mind.

Together, these findings establish that MnO₂@PMS not only eradicates mature biofilms in vivo but also mitigates infection-associated tissue injury while maintaining a favourable short-term safety profile in this 14-day model. Having demonstrated efficacy in infection control and tissue preservation, we next examined its influence on the adaptive immune landscape within infected hosts.

### MnO₂@PMS reverses in vivo immunosuppression and promotes adaptive immune activation and tissue regeneration

**Fig. 8:**
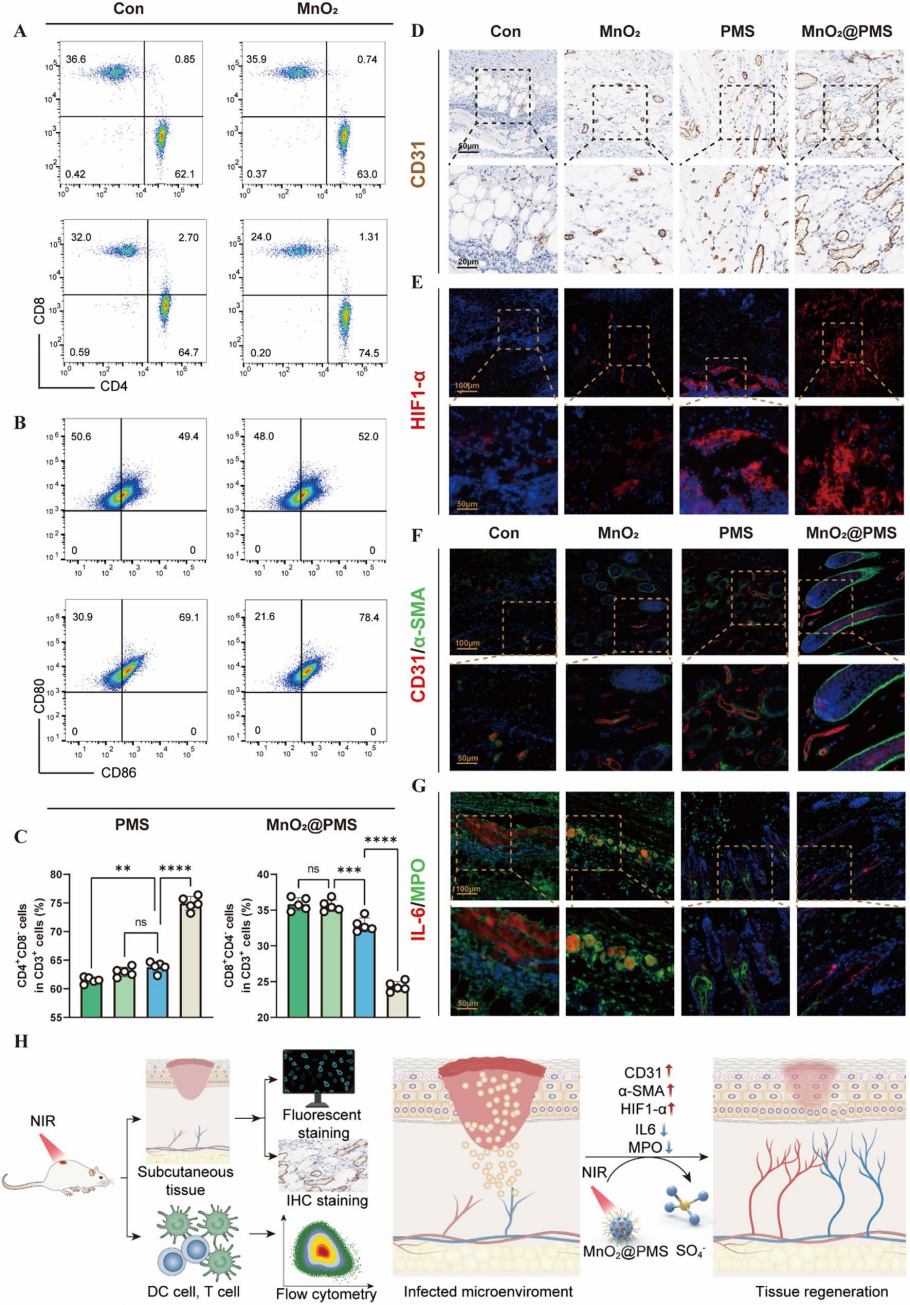
MnO₂@PMS modulates the immune microenvironment and promotes tissue repair in vivo. **(A)** Representative flow cytometry plots of CD4⁺ and CD8⁺ T-cell subpopulations isolated from draining lymph nodes on day 14 post-treatment with PBS (control), MnO₂, PMS, or MnO₂@PMS, showing enhanced T-cell activation and infiltration in the MnO₂@PMS group. **(B)** Representative flow cytometry plots of CD80⁺ and CD86⁺ dendritic cells (DCs) derived from lymph nodes, indicating increased DCs maturation following MnO₂@PMS treatment. **(C)** Quantification of CD4⁺ and CD8⁺ T-cell proportions among CD3⁺ cells in each group (*n* = 5), showing statistically significant elevation in effector T-cell populations following MnO₂@PMS treatment. **(D)** Immunohistochemical staining of CD31 in peri-implant tissues to assess neovascularization. MnO₂@PMS enhanced CD31⁺ microvascular structures compared to controls. Scale bars: 50 μm (overview), 25 μm (magnified view). **(E)** Immunofluorescence staining of hypoxia-inducible factor 1-alpha (HIF-1α, red) in peri-implant tissues with DAPI (blue) counterstaining. Increased HIF-1α expression suggests active tissue remodeling and metabolic adaptation rather than a passive hypoxic response. **(F)** Dual immunofluorescence staining for CD31 (red) and α-smooth muscle actin (α-SMA, green) to visualize vascular endothelial and perivascular cell distribution. MnO₂@PMS-treated tissues displayed more organized vasculature and increased perivascular cell presence. **(G)** Dual immunofluorescence staining of IL-6 (red) and myeloperoxidase (MPO, green), demonstrating modulation of inflammatory cytokine production and reduced neutrophil infiltration in the MnO₂@PMS group. **(H)** Schematic diagram summarizing the in vivo experimental workflow and analysis endpoints. Following MnO₂@PMS treatment and NIR activation, downstream assessments—including lymph node immune profiling, peri-implant tissue staining, and cytokine quantification—demonstrate that MnO₂@PMS modulates the local immune microenvironment, enhances T-cell and DCs activation, promotes vascular remodeling, and facilitates tissue repair through sulfate radical (•SO₄⁻)–mediated mechanisms. Data are presented as means ± SD. Statistical significance was assessed using one-way ANOVA (**C**). *P < 0.05, **P < 0.01, ***P < 0.001, ***P < 0.0001; ns, not significant

Building on our in vitro findings, we next assessed whether MnO₂@PMS could similarly remodel the immunosuppressive microenvironment in vivo. Flow cytometric analysis of draining lymph nodes revealed marked reactivation of adaptive immunity in MnO₂@PMS-treated mice. Specifically, these animals exhibited significantly higher frequencies of CD80⁺CD86⁺ mature dendritic cells in lymph nodes, alongside increased CD4⁺ and CD8⁺ T-cell populations in draining lymph nodes compared with controls (Fig. 8A–C). These results indicate recovery of antigen-presenting cell (APC) function and subsequent T-cell priming in treated mice. Such restoration of DCs co-stimulatory activity and T-cell responses is critical, as IL-10–driven immunosuppression in chronic infections typically downregulates DCs cytokine production and co-stimulatory molecule expression, thereby blunting T-cell activation [13]. Consistent with this, a robust T-cell response is pivotal for resolving persistent *S. aureus* infections [41]. The gating strategy for lymph node analyses is provided in Figure S31 and ensures rigorous identification of T-cell subsets. Consistent with the antibacterial readouts, MnO₂- and PMS-treated mice showed only partial recovery of effector T-cell responses and dendritic-cell maturation, whereas MnO₂@PMS treatment induced the most robust expansion of CD4⁺ and CD8⁺ T cells and a marked shift toward an immunostimulatory DC phenotype (Figs. 8 and S31–S36).”

In parallel, MnO₂@PMS-treated tissues displayed distinct microenvironmental shifts that favored repair and regeneration. Immunofluorescence staining showed robust neovascular remodeling at infection sites, characterized by abundant CD31⁺ endothelial cells and α-SMA⁺ perivascular cells forming new vessels (Fig. 8D–F). HIF-1α expression was also elevated in treated tissues, consistent with adaptive responses to hypoxia that promote angiogenesis and tissue recovery [42]. As a master regulator of angiogenic growth factors, HIF-1α stabilization under low oxygen conditions stimulates capillary formation and enhances perfusion [42]. At the same time, pro-inflammatory injury markers were attenuated. MnO₂@PMS-treated tissues exhibited significantly reduced IL-6 and myeloperoxidase (MPO) levels (Fig. 8G; Figures S36–S37). IL-6 is a key pro-inflammatory cytokine, and MPO is a neutrophil-derived granule enzyme; both contribute to neutrophil infiltration and collateral tissue damage in infected or inflamed sites [43, 44]. Excessive neutrophil activation can drive MPO release and cytokine storms that exacerbate host injury [43], with high IL-6 and MPO levels correlating with greater disease severity [44]. Thus, suppression of these markers in MnO₂@PMS-treated mice suggests attenuation of neutrophil-dominated inflammation and a shift toward a regenerative, tissue-preserving microenvironment.

Mechanistically, the immunoregulatory and reparative effects of MnO₂@PMS are best explained by a combination of host metabolic–immune reprogramming and cGAS–STING pathway activation rather than by a single linear signalling event. Manganese is known to potentiate the cGAS–STING cytosolic DNA-sensing pathway in innate immunity [45], and MnO₂@PMS likely contributes to this stimulation. Consistent with prior reports that Mn²⁺ enhances DNA-triggered cGAS–STING activation, our DC experiments showed that MnO₂@PMS alone did not significantly upregulate Ifnb1 or Cxcl10, but markedly amplified their induction in the presence of exogenous bacterial dsDNA (Fig. 5I), supporting a cofactor role for Mn²⁺ rather than a stand-alone STING agonist in our system. At the same time, our findings are consistent with MnO₂@PMS attenuating an immunosuppressive metabolic circuit established by the biofilm—particularly involving glutamate–IL-10–associated signalling—which we regard as one important contributor, rather than the sole driver, of immune reactivation. We did not, however, directly block glutamate or IL-10 signalling in this study. Thus, although our data are consistent with this axis contributing substantially to immune reactivation, it should be regarded as one important component rather than the sole pathway involved. In *vitro*, MnO₂@PMS significantly reduced biofilm-derived glutamate accumulation and IL-10 production in immune cells, thereby breaking a key metabolic–immunologic axis of tolerance. Pathogenic biofilms often exploit metabolic byproducts to trigger IL-10 release from myeloid subsets, thereby blunting immune activation [46]. For instance, *S. aureus* biofilm-derived lactate has been shown to induce IL-10 expression in granulocytic (G)-MDSCs, generating a tolerogenic milieu that impairs bacterial clearance [46]. Our data support a parallel role for glutamate, which promoted IL-10 production and sustained a tolerogenic APC phenotype.

The reversal of this glutamate–IL-10 axis by MnO₂@PMS likely contributes importantly to alleviating DC tolerization and restoring effective innate and adaptive immune responses, in concert with other MnO₂@PMS-induced signals. IL-10 is well established as a brake on DCs, suppressing their inflammatory cytokine output and downregulating antigen-presenting co-stimulatory molecules, which limits T-cell priming. By relieving this metabolic checkpoint, MnO₂@PMS effectively re-licensed immune cells to sense cytosolic DNA and produce inflammatory cytokines, thereby indirectly amplifying DNA-sensing pathways such as cGAS–STING. The end result is a reactivation of both innate and adaptive arms of immunity, promoting pathogen clearance alongside a pro-regenerative immune profile.

Supporting this model, recent studies demonstrate that blocking immunosuppressive metabolic pathways in biofilm infections, for example, by inhibiting G-MDSC glycolysis or HIF-1α, skews immune cells toward a pro-inflammatory phenotype and enhances bacterial clearance [46]. Such metabolic–immunologic coupling remains an underexplored therapeutic target in biofilm management [47], where most approaches focus either on bactericidal strategies or immune receptor stimulation without addressing immunometabolic dysfunction. Our findings highlight that correcting the host’s immunometabolic state can synergize with direct antimicrobial effects, supporting metabolic–immune modulation as one promising therapeutic avenue that complements existing nanozyme and photothermal strategies.

Collectively, these in vivo results demonstrate that MnO₂@PMS coordinates bacterial clearance with immune reactivation through modulation of metabolic–immune crosstalk, culminating in tissue repair and infection resolution. By simultaneously debulking biofilms and reversing local immune paralysis, MnO₂@PMS helps convert chronically suppressed infection sites into microenvironments more capable of mounting defensive and reparative responses in this infection model. These results warrant further evaluation of MnO₂@PMS in clinically relevant deep-tissue infection models, such as orthopedic implant infections, to establish its translational potential against recalcitrant biofilm-associated infections. Importantly, no systemic or local antibiotics were administered in any group, so these data reflect the intrinsic efficacy of the nanoplatform in this implant infection model and do not constitute a direct head-to-head comparison with standard-of-care antibiotic regimens. Within this context, MnO₂@PMS should be viewed as adding an immunometabolic dimension to these established nanozyme and photothermal strategies by explicitly targeting the glutamate–IL-10–STING axis in a chronic S. aureus biofilm setting.

Despite these encouraging findings, several limitations should be acknowledged before clinical translation. First, although serum biochemistry and histological examination of major organs did not reveal overt systemic toxicity within the 14-day observation window in our model, the long-term biodistribution, accumulation and clearance of Mn species were not investigated, and potential delayed or chronic effects remain undefined; dedicated pharmacokinetic, biodistribution and chronic-exposure studies in larger animal models will be required to delineate a safe therapeutic window. Second, possible off-target actions of MnO₂@PMS on immune and stromal compartments in non-infected tissues were not systematically assessed and will need to be evaluated by broader toxicology and immunoprofiling. Third, the present hollow MnO₂@PMS synthesis was optimised at laboratory scale, and issues such as batch-to-batch reproducibility, upscaling under GMP-compatible conditions and stringent quality control have yet to be addressed. Finally, translation of sulfate-radical–generating, metal-based nanoplatforms will inevitably face regulatory scrutiny regarding oxidative stress and metal ion release, underscoring the need for rigorous safety evaluation and careful integration with conventional antibiotic regimens in future studies.

## Conclusion

In this work, we introduce a near-infrared (NIR)-responsive MnO₂@PMS nanoregulator that addresses the dual barriers of *Staphylococcus aureus* biofilm infection: structural resilience and immune suppression. Under photothermal/photosensitizer co-activation, MnO₂@PMS catalyzes the production of oxygen-independent sulfate radicals (•SO₄⁻), enabling efficient matrix degradation and bacterial eradication within dense, hypoxic biofilms. Beyond direct antimicrobial effects, MnO₂@PMS reshapes the immunosuppressive microenvironment by intercepting bacterial glutamate metabolism, thereby reducing PGA synthesis and weakening a metabolic–immune checkpoint that contributes to IL-10 overproduction and tolerogenic dendritic-cell polarization. This immunometabolic modulation re-engages cytosolic DNA sensing via the cGAS–STING pathway, restores dendritic cells maturation and antigen presentation, and revives downstream CD4⁺ and CD8⁺ T-cell activation. In vivo, MnO₂@PMS therapy not only achieved rapid and durable bacterial clearance but also did not show obvious biosafety issues within the 14-day observation period. Collectively, these findings illustrate a dual-action strategy that couples biofilm disruption with immune reactivation and identify MnO₂@PMS as a promising candidate for further preclinical evaluation in recalcitrant biofilm-associated infections. However, we did not include a dedicated antibiotic-treated arm in this study, and the present data therefore do not allow definitive conclusions regarding clinical superiority over standard antibiotic regimens; future work will be needed to benchmark MnO₂@PMS alone or in combination with conventional therapies in clinically relevant models.

## Supplementary Information

Supplementary Material 1

Supplementary Material 2

## Data Availability

The datasets used and/or analysed during the current study are available from the corresponding author on reasonable request.

## Abbreviations

MnO₂@PMS: A near-infrared-responsive nanoregulator composed of MnO₂ and peroxymonosulfate, enabling oxygen-independent sulfate radical generation for antibiofilm catalysis
PMS (Peroxymonosulfate): A strong oxidant precursor that decomposes under MnO₂ catalysis to yield reactive sulfate radicals (SO₄•⁻)
SO₄•⁻ (Sulfate radical): A high-redox, oxygen-independent reactive species capable of penetrating biofilm matrices and inducing oxidative bactericidal activity
MIT (Metabolic immunotherapy): A therapeutic concept integrating metabolic reprogramming and immune activation to overcome infection-induced immune suppression
cGAS–STING pathway: A cytosolic DNA-sensing axis (cyclic GMP–AMP synthase and stimulator of interferon genes) that activates type I interferon signaling and innate immunity
IL-10 (Interleukin-10): An anti-inflammatory cytokine that suppresses dendritic-cell activation and promotes immune tolerance in chronic infections
DCs (Dendritic cells): Professional antigen-presenting cells that orchestrate innate and adaptive immune responses through antigen presentation and cytokine secretion
PGA (Poly-γ-glutamic acid): A glutamate-derived extracellular polymer secreted by Staphylococcus aureus, contributing to biofilm stability and immune evasion
ROS (Reactive oxygen species): Chemically reactive oxygen-containing molecules, including sulfate and hydroxyl radicals, responsible for oxidative bacterial killing
NIR (Near-infrared): Electromagnetic radiation (700–1100 nm) used to trigger photothermal or photocatalytic activity of nanomaterials within biological tissues
MRSA (Methicillin-resistant Staphylococcus aureus): A multidrug-resistant bacterial strain forming persistent biofilms on medical implants
