# Supplementary material for: Glutamate nanoregulator for metabolic immunotherapy of biofilm-associated implant infections
Source: J Nanobiotechnology. 2026 Feb 4;24:123. doi: 10.1186/s12951-025-04016-3 (PMC12879480; doi:10.1186/s12951-025-04016-3)
Supplement: Supplementary file 1 — Supplementary Material 1 [file 12951_2025_4016_MOESM1_ESM.docx]

Supplementary File 2. Uncropped original Western blot images corresponding to Figure 6-J in the main manuscript.

STING：







p-STING：







p-STING-GAPDH：







TBK1：







p-TBK1：







p-TBK1-GAPDH：







IRF3：







p-IRF3：







p-IRF3-GAPDH：







p65：







p-p65：







p-p65-GADPH：
