# Supplementary material for: Glutamate nanoregulator for metabolic immunotherapy of biofilm-associated implant infections
Source: J Nanobiotechnology. 2026 Feb 4;24:123. doi: 10.1186/s12951-025-04016-3 (PMC12879480; doi:10.1186/s12951-025-04016-3)
Supplement: Supplementary file 2 — Supplementary Material 2 [file 12951_2025_4016_MOESM2_ESM.docx]

Supporting Information

Glutamate Nanoregulator for Metabolic Immunotherapy of Biofilm-Associated Implant Infections

Heng Wu, Jiahao Chen, Xiao Ma, Haijian Li, Qiao Wu, Zhenyu Jiang, Tianyu Xi, Chi Zhang*, Geyong Guo*, and Pei Han*

**Experimental Section**

**Reagents and Kits.**

The CCK-8 assay kit was obtained from E-zyme Biotech (China). Crystal violet, ROS detection kit, and TRITC/FITC–phalloidin (for cytoskeletal staining) were purchased from Beyotime Biotechnology (Shanghai, China). Methylene blue (MB) was obtained from Aladdin Reagent Co., Ltd. (Shanghai, China). The Live/Dead Bacterial Viability Kit (BacLight™) was purchased from ThermoFisher Scientific (Waltham, USA). The TSA fluorescence double staining kit was obtained from ABclonal (Wuhan, China), and the Annexin V-FITC/PI apoptosis detection kit was from YEASEN Biotechnology (Shanghai, China). ELISA kits for cytokine quantification were purchased from Dakewe Biotech (Shenzhen, China). Primers for quantitative PCR (qPCR) were synthesized by Biosune Biotechnology Co., Ltd. (Shanghai, China). All antibodies used for flow cytometry were procured from BioLegend (San Diego, USA).

**Materials.**

Hollow manganese dioxide nanoparticles (MnO₂ NPs), polyethylenimine (PEI), monopersulfate (PMS, Oxone®; KHSO₅·0.5KHSO₄·0.5K₂SO₄), and methylene blue (MB) were purchased from Aladdin Chemical Co., Shanghai, China. Ethanol, tert-butyl alcohol (TBA), phosphate, and hydrogen peroxide (H₂O₂) were obtained from Shanghai McLean Biochemical Co., Ltd.

**Synthesis of MnO₂@PMS Nanoparticles.**

To prepare MnO₂@PMS-PEI nanoparticles, 20 mL of PEI solution (1 mg/mL) was added dropwise to 20 mL of MnO₂ nanoparticle suspension (1 mg/mL) under continuous stirring. The mixture was stirred for 6 hours at room temperature, followed by five cycles of centrifugation and washing with deionized (DI) water to obtain MnO₂-PEI complexes. Subsequently, MnO₂@PMS nanoparticles were synthesized by mixing PMS powder with the MnO₂-PEI solution at a mass ratio of 1:1 (w/w). The reaction was allowed to proceed for 60 minutes at room temperature. The final MnO₂@PMS product was collected by centrifugation at 11,000 rpm for 5 minutes and washed four times with DI water to remove unbound reagents.

**Characterization of MnO₂@PMS Nanoparticles.**

The morphology and elemental distribution of MnO₂@PMS nanoparticles were analyzed using scanning electron microscopy (SEM) and transmission electron microscopy (TEM) (JEM-2100F, JEOL, Japan). Zeta potential and hydrodynamic diameter were measured using a Zetasizer Nano ZS90 (Malvern Instruments, UK). The release concentration of manganese ions (Mn²⁺) was determined by inductively coupled plasma atomic emission spectroscopy (ICP-AES, iCAP 7000 Series, Thermo Scientific, USA).
UV–visible absorption spectra were recorded using a Cary 300 UV–Vis spectrophotometer (Agilent Technologies, USA). For ROS-related analysis, phosphate buffer (pH 6.5) was prepared by adjusting deionized water. PMS (1 mmol) and hydrogen peroxide (1 mmol) were separately added to 1 mL of methylene blue (MB) solution under identical conditions. UV–Vis spectra were collected over the range of 500–800 nm to monitor changes in absorbance. The release kinetics of HSO₄⁻ and OH⁻ were evaluated at time points of 0, 10, and 30 minutes.

**Cell lines**

Murine dendritic cell line DC2.4 was kindly provided by the Orthopaedics Research Laboratory of Shanghai Sixth People’s Hospital and originally obtained from the American Type Culture Collection (ATCC, CRL-11921). The cells were derived from female C57BL/6 mice and routinely maintained in RPMI-1640 medium supplemented with 10% fetal bovine serum (FBS; Gibco) and 1% penicillin–streptomycin (Gibco) at 37 °C in a humidified incubator with 5% CO₂. Experiments were performed using cells between passages 3–10.

Bone marrow-derived dendritic cells (BMDCs) were isolated from the femurs and tibias of 6–8-week-old female C57BL/6 mice (Shanghai SLAC Laboratory Animal Co., Ltd., China) under sterile conditions. Bone marrow cells were flushed with RPMI-1640, filtered through a 70 µm cell strainer, and cultured in RPMI-1640 containing 10% FBS, 1% penicillin–streptomycin, and 20 ng/mL granulocyte–macrophage colony-stimulating factor (GM-CSF; PeproTech). Cells were differentiated for 6–7 days before use.

**Cytotoxicity assay.**

To assess the cytocompatibility of MnO₂@PMS nanoparticles, murine dendritic cells (DC2.4) were seeded into 96-well plates at a density of 1 × 10⁴ cells per well and incubated for 12 hours at 37 °C in a humidified atmosphere containing 5% CO₂. After initial adhesion, the culture medium was replaced with fresh complete DMEM containing MnO₂@PMS nanoparticles at various concentrations (0, 1, 3, 5, 10, 30, 50, 75, and 100 μg/mL). Cells were then incubated for an additional 24 hours under the same conditions.

Following treatment, cells were gently washed twice with phosphate-buffered saline (PBS) to remove any residual nanoparticles. Subsequently, 100 μL of CCK-8 working solution (prepared according to the manufacturer's instructions; E-zyme Biotech, China) was added to each well. Plates were incubated in the dark at 37 °C for 1.5 hours, and absorbance at 450 nm was measured using a microplate reader (Thermo Scientific, USA). Cell viability was calculated by normalizing the absorbance of each treatment group to that of the untreated control (0 μg/mL). All experiments were performed in quintuplicate (n = 5), and results are presented as mean ± standard deviation (SD).

**Antibacterial Assay *in vitro*.**

To evaluate the antibacterial efficacy of MnO₂@PMS nanoparticles against planktonic *Staphylococcus aureus* (MRSA, ATCC 43300), a series of *in vitro* assays were conducted.

**Bacterial culture and treatment.**

Methicillin-resistant *Staphylococcus aureus* (MRSA, ATCC 43300) was cultured in tryptic soy broth (TSB; Hopebio, China) at 37 °C with shaking at 200 rpm overnight. The bacterial suspension was then diluted in fresh TSB to approximately 1 × 10⁷ colony-forming units (CFU)/mL for subsequent experiments.

For the PT–PS synergistic antibacterial assay, 1 mL of the MRSA suspension was incubated in 1.5 mL centrifuge tubes with one of the following treatments: PBS (control), MnO₂ (50 μg/mL), PMS (25 μg/mL, Oxone®; corresponding to the estimated PMS content in the MnO₂@PMS composite), or MnO₂@PMS (50 μg/mL). After 30 minutes of incubation at room temperature, samples were irradiated with an 808 nm near-infrared (NIR) laser at a power density of 1.0 W/cm² for 5 minutes.

**Transmission and scanning electron microscopy (TEM/SEM).**

For ultrastructural analysis, bacterial samples after treatment were collected by centrifugation at 5000 rpm for 5 minutes, washed twice with phosphate-buffered saline (PBS), and fixed in 2.5% glutaraldehyde at 4 °C overnight. The fixed samples were then dehydrated through a graded ethanol series (30%, 50%, 70%, 80%, 90%, 95%, and 100%), followed by vacuum drying. Morphological and ultrastructural features were examined using transmission electron microscopy (TEM; JEM-2100, JEOL, Japan) and field-emission scanning electron microscopy (FE-SEM; Regulus 8230, Hitachi, Japan).

**Colony-forming unit (CFU) assay.**

After treatment, bacterial suspensions were serially diluted (10⁻¹ to 10⁻⁵) in sterile phosphate-buffered saline (PBS). A 100 μL aliquot from each dilution was spread evenly onto tryptic soy agar (TSA) plates. The plates were incubated at 37 °C for 24 hours, after which the number of viable colonies was counted. Bacterial viability was expressed as colony-forming units per milliliter (CFU/mL).

**SYTO9/PI staining and flow cytometry.**

Bacterial viability was further assessed using the BacLight™ Bacterial Viability Kit (ThermoFisher Scientific, USA) according to the manufacturer's protocol. Briefly, 1 mL of treated bacterial suspension was stained with 3 μL SYTO9 and 3 μL propidium iodide (PI), incubated in the dark for 15 min, and analyzed using a flow cytometer (CytoFLEX LX, Beckman Coulter, USA). SYTO9⁺/PI⁻ signals represented live bacteria, whereas SYTO9⁺/PI⁺ or SYTO9⁻/PI⁺ indicated membrane-compromised, dead cells.

All experiments were independently performed at least three times, and data were expressed as mean ± standard deviation (SD). Statistical analyses were conducted using one-way ANOVA with Tukey’s post hoc test.

***In vitro* Biofilm Disruption Assay.**

To evaluate the biofilm-disrupting capability of MnO₂@PMS nanoparticles under near-infrared (NIR) irradiation, a multimodal analysis was conducted, including scanning electron microscopy (SEM), crystal violet staining, and live/dead bacterial staining followed by confocal laser scanning microscopy (CLSM).

**SEM Imaging of Biofilm Morphology.**

Sterile glass coverslips (used as cell climbing substrates) were placed in 24-well plates. Each well was inoculated with 1 mL of *Staphylococcus aureus* suspension (1 × 10⁷ CFU/mL) prepared in tryptic soy broth (TSB) supplemented with 1% glucose. After incubation at 37 °C for 24 hours to allow biofilm formation, the coverslips were gently rinsed with phosphate-buffered saline (PBS) to remove non-adherent bacteria.

The established biofilms were then treated with PBS (control), MnO₂ (50 μg/mL), PMS (25 μg/mL), or MnO₂@PMS (50 μg/mL), with or without 808 nm NIR laser irradiation (1.0 W/cm², 5 min). After treatment, the samples were fixed in 2.5% glutaraldehyde at 4 °C overnight, dehydrated through a graded ethanol series (30%–100%), subjected to critical point drying, and gold sputter coated. The biofilm surface morphology was examined using a field-emission scanning electron microscope (FE-SEM; Regulus 8230, Hitachi, Japan).

**Crystal Violet Quantification of Biofilm Biomass.**

*Staphylococcus aureus* biofilms were established in 96-well plates under the same conditions as described above. After treatment with different materials, with or without 808 nm near-infrared (NIR) laser irradiation (1.0 W/cm², 5 min), wells were gently rinsed with phosphate-buffered saline (PBS), air-dried, and stained with 0.1% (w/v) crystal violet solution (Beyotime, China) for 20 minutes at room temperature. Excess dye was removed by thorough rinsing with PBS, and the retained stain was solubilized in 33% acetic acid. Absorbance at 590 nm was measured using a microplate reader (Thermo Scientific, USA) to quantify residual biofilm biomass.

**Live/Dead Biofilm Imaging via Confocal Laser Scanning Microscopy (CLSM).**

For live/dead staining, mature biofilms grown on glass coverslips were treated as described above and stained using the BacLight™ Bacterial Viability Kit (SYTO9/PI; ThermoFisher Scientific, USA). Coverslips were incubated with the dye mixture in the dark for 15 minutes at room temperature, rinsed gently with PBS, and mounted for observation. Fluorescence imaging was performed using a confocal laser scanning microscope (CLSM; Leica TCS SP8, Germany). Live bacteria with intact membranes were stained green by SYTO9, whereas dead bacteria with compromised membranes were stained red by propidium iodide (PI).

All experiments were independently performed in triplicate unless otherwise stated.

**Reactive Oxygen Species (ROS) Detection Assays.**

The generation of reactive oxygen species (ROS) by MnO₂@PMS nanocomposites was evaluated under both acidic and biofilm-mimicking conditions.

**ROS Detection in Solution (pH 6.5).**

To simulate the acidic microenvironment of biofilms, the generation of reactive oxygen species (ROS) by MnO₂@PMS nanocomposites was evaluated in a pH 6.5 HEPES buffer system. MnO₂@PMS dispersions (50 μg/mL) were exposed to 808 nm near-infrared (NIR) laser irradiation (1.0 W/cm²) for varying durations (0, 10, and 30 minutes). Methylene blue (MB; Aladdin, China) was used as a general redox indicator, and its absorbance at 665 nm was measured using a UV–Vis spectrophotometer (UV-2600, Shimadzu, Japan) to assess MB degradation as an indirect measure of total ROS production.

To identify the dominant radical species, ethanol (EtOH, 0.1 M) and tert-butanol (TBA, 0.1 M) were added as radical scavengers. EtOH effectively quenches both hydroxyl radicals (•OH) and sulfate radicals (•SO₄⁻), whereas TBA selectively scavenges •OH. Quencher-containing mixtures were subjected to the same irradiation conditions, and MB degradation kinetics were analyzed. A greater inhibition of MB degradation in the presence of EtOH compared to TBA was interpreted as indicative of •SO₄⁻ being the predominant radical species generated. All reaction solutions were freshly prepared and maintained at 37 °C throughout the experiment.

**ROS Detection in Biofilm Conditions.**

Mature MRSA biofilms were cultured in 24-well plates and treated with MnO₂@PMS nanoparticles (50 μg/mL) under 808 nm near-infrared (NIR) laser irradiation (1 W/cm²) for 5 min. After subsequent incubation at 37 °C for 6 and 12 h, total ROS generation was assessed using a ROS fluorescent detection kit (Beyotime, Shanghai, China) according to the manufacturer’s protocol. Briefly, biofilms were incubated with the DCFH-DA probe, and fluorescence intensity was measured at excitation/emission wavelengths of 488/525 nm using a microplate reader (Synergy H1, BioTek, USA). Radical-specific scavengers were not applied due to potential interference from the extracellular polymeric substances in the biofilm matrix.

All assays were conducted in triplicate unless otherwise noted.

**Biofilm Metabolite Profiling and Biochemical Validation.**

To explore metabolomic alterations induced by nanomaterial treatment in the biofilm microenvironment, bacterial biofilms were established in 6-well plates and treated with PBS and MnO₂@PMS under 808 nm NIR irradiation (1 W/cm², 5 min). After incubation, biofilm-associated material was collected and processed for metabolomics in accordance with the sample preparation guidelines provided by Novogene Co., Ltd. (Beijing, China). Samples were rapidly frozen in liquid nitrogen and sent for untargeted metabolomic analysis using liquid chromatography–mass spectrometry (LC-MS/MS). Pathway enrichment analysis highlighted glutamate metabolism as a key differential pathway.

To validate these findings, selected metabolites including glutamate, glutamine, γ-aminobutyric acid (GABA), and α-ketoglutarate were quantitatively assessed using commercial biochemical kits from Jiancheng Bioengineering Institute (Nanjing, China), UnionBio (Shanghai, China), and Beyotime (Shanghai, China), respectively. Assays were conducted following the manufacturers’ protocols, and absorbance was measured using a microplate reader (Thermo Scientific, USA). Quantitative results were obtained by comparison to standard curves.

**Flow Cytometric Analysis of Dendritic Cell Surface Markers.**

To assess dendritic cell (DC) maturation and phenotypic differentiation, flow cytometry was performed using murine DC2.4 cells and bone marrow–derived dendritic cells (BMDCs). BMDCs were isolated from the femurs and tibias of 6–8-week-old BALB/c mice (SPF grade), flushed with RPMI-1640 medium, filtered through a 70 μm strainer, and cultured for 6 days in RPMI-1640 supplemented with 10% fetal bovine serum (FBS), 20 ng/mL GM-CSF, and 10 ng/mL IL-4 (PeproTech, USA).

DC2.4 cells and BMDCs were seeded at a density of 1 × 10⁶ cells per well in 6-well plates. Mature Staphylococcus aureus biofilms were first treated with PBS, MnO₂, PMS, or MnO₂@PMS under 808 nm NIR irradiation (1 W/cm², 5 min), followed by incubation at 37 °C for 12 hours. The resulting biofilm supernatants were collected, filtered through a 0.22 μm membrane to remove residual bacteria, and diluted 1:4 with fresh culture medium. These conditioned media were then used to culture DC2.4 and BMDCs for 24 h. For glutamate rescue groups, 200 μM exogenous glutamate (Sigma-Aldrich, USA) was added simultaneously.

After incubation, cells were harvested and blocked with anti-CD16/32 (clone 93, BioLegend, USA) to prevent nonspecific binding, followed by staining with fluorophore-conjugated antibodies targeting CD11c, CD80, CD86, CD206, and CCR7 (BioLegend, USA). Flow cytometry was performed on a CytoFLEX system (Beckman Coulter, USA), and data were analyzed using FlowJo software (Tree Star, USA).

**Immunofluorescence Analysis of STING and Surface Markers in DC2.4 Cells.**

To assess intracellular STING localization, cytoskeletal remodeling, and the co-expression of CD206 and CD40 in DC2.4 cells, immunofluorescence staining was performed using conventional antibody staining and tyramide signal amplification (TSA)–based double staining approaches.

**STING and cytoskeletal immunofluorescence**.
DC2.4 cells were seeded onto sterilized glass coverslips in 24-well plates and cultured in conditioned medium derived from mature *Staphylococcus aureus* biofilms treated with PBS, MnO₂, PMS, or MnO₂@PMS under 808 nm near-infrared (NIR) irradiation (1.0 W/cm², 5 min), followed by 12 h incubation. Biofilm supernatants were collected, filtered (0.22 μm), and diluted 1:4 in complete culture medium. After 24 h of stimulation, cells were fixed with 4% paraformaldehyde for 20 min at room temperature and washed three times with PBS.

For STING staining, cells were permeabilized with 0.1% Triton X-100 in PBS for 10 min and blocked with 5% bovine serum albumin (BSA) for 1 h. Samples were incubated overnight at 4 °C with rabbit anti-STING primary antibody (1:200, CST, USA), followed by Alexa Fluor 594–conjugated goat anti-rabbit IgG secondary antibody (1:500, Abcam, UK) for 1 h at room temperature in the dark. Cell nuclei were counterstained with DAPI (1 μg/mL, Beyotime, China) for 10 min.

For visualization of cytoskeletal structure, cells were stained with TRITC- or FITC-conjugated phalloidin (1:100 in PBS, Beyotime, China) for 30 min at room temperature. All staining steps included three PBS washes. Fluorescence images were acquired using a confocal laser scanning microscope (Leica TCS SP8, Germany) to evaluate intracellular STING distribution and cytoskeletal organization.

**TSA double immunofluorescence staining for CD206 and CD40.**
To investigate the co-expression of surface markers CD206 and CD40, TSA-based double staining was performed using the TSA Fluorescence Double Staining Kit (ABclonal, RK05902, Wuhan, China), according to the manufacturer's protocol. DC2.4 cells were seeded onto sterile coverslips and cultured overnight. Cells were then incubated for 24 h in conditioned media as described above.

After fixation with 4% paraformaldehyde, cells were permeabilized with 0.1% Triton X-100 and incubated with 3% H₂O₂ for 10 min to block endogenous peroxidase activity. Non-specific binding was blocked using 5% BSA for 30 min.

For CD40 detection, cells were incubated overnight at 4 °C with rabbit polyclonal anti-CD40 antibody (Cat# A13285, ABclonal, IHC-validated). After washing, an HRP-conjugated secondary antibody was applied, followed by TSA-488 reagent for signal amplification (green fluorescence). CD206 detection was then performed sequentially using rabbit monoclonal anti-CD206 antibody (Cat# A21014, ABclonal), an HRP-conjugated secondary antibody, and TSA-594 reagent (red fluorescence). Cell nuclei were counterstained with DAPI (RM02978, ABclonal, China), and samples were mounted with anti-fade medium.

Images were captured with a confocal microscope (Leica TCS SP8). Green fluorescence indicated CD40⁺ cells, red fluorescence indicated CD206⁺ cells, and blue fluorescence represented DAPI-stained nuclei.

**Cytokine Quantification by ELISA.**

Culture supernatants from DC2.4 or BMDCs subjected to various treatments were collected after 24 h of incubation. Cytokine levels of IL-6, IL-10, and TNF-α were measured using commercial ELISA kits (Dakewe Biotech, Shenzhen, China) following the manufacturer’s instructions. Briefly, standards and samples were added to pre-coated 96-well plates, incubated with detection antibodies and HRP-conjugates, and developed using TMB substrate. The absorbance at 450 nm was recorded using a microplate reader (Thermo Scientific, USA). All samples were measured in triplicate and expressed as mean ± SD.

**Transcriptomic Analysis.**

To investigate the transcriptional mechanisms underlying dendritic cell activation, RNA sequencing was conducted on DC2.4 cells exposed to biofilm-derived conditioned media from either PBS- or MnO₂@PMS-treated groups. Mature *Staphylococcus aureus* biofilms were first treated with PBS or MnO₂@PMS under 808 nm NIR irradiation (1 W/cm², 5 min), followed by incubation for 12 h. The resulting supernatants were collected, filtered through 0.22 μm membranes, and diluted 1:4 in complete RPMI-1640 medium supplemented with 10% FBS.

DC2.4 cells were seeded at 1 × 10⁶ cells/well in 6-well plates and allowed to adhere overnight. The next day, the culture medium was carefully removed, and cells were gently washed twice with sterile PBS to eliminate residual serum and unattached cells. Then, 2 mL of the conditioned medium was added to each well, and cells were incubated for 24 h under standard culture conditions (37 °C, 5% CO₂).

After treatment, cells were washed with PBS, and total RNA was extracted using TRIzol reagent (Invitrogen, USA) according to the manufacturer’s protocol. RNA concentration and integrity were assessed using a NanoDrop spectrophotometer and Agilent 2100 Bioanalyzer (Agilent Technologies, USA).

Qualified RNA samples were submitted to Novogene (Beijing, China) for sequencing on the Illumina NovaSeq 6000 platform. Clean reads were mapped to the mouse reference genome (GRCm39), and differentially expressed genes were identified using DESeq2. Genes with |log₂FoldChange| ≥ 1 and adjusted p-value < 0.05 were considered significant. Gene Ontology (GO) and KEGG pathway enrichment analyses were performed to explore biological functions and pathways affected by the treatment.

**Western Blot Analysis.**

To assess protein-level changes in immune signaling pathways, Western blotting was performed using total protein lysates from DC2.4 cells cultured in conditioned media derived from biofilm supernatants. Specifically, mature *Staphylococcus aureus* biofilms were treated with PBS, PMS, MnO₂, or MnO₂@PMS under 808 nm NIR irradiation (1.0 W/cm², 5 min), followed by overnight incubation at 37 °C. The resulting supernatants were collected, filtered through 0.22 μm membranes, and diluted 1:4 in complete culture medium. DC2.4 cells were seeded in 6-well plates and incubated with the conditioned media for 24 h.

Following incubation, cells were gently washed with PBS and lysed using RIPA buffer (Beyotime, China) supplemented with protease and phosphatase inhibitors. Protein concentrations were determined using a BCA Protein Assay Kit (Beyotime, China).

Equal amounts of protein (20–30 μg) were subjected to SDS–PAGE and transferred onto PVDF membranes (Millipore, USA). Membranes were blocked with 5% non-fat milk for 1 h at room temperature and incubated overnight at 4 °C with primary antibodies against STING, TBK1, p-TBK1, IRF3, p-IRF3, and GAPDH (Cell Signaling Technology, CST, USA). HRP-conjugated secondary antibodies (CST, USA) were then applied for 1 h at room temperature. Immunoreactive bands were detected using ECL reagents (Bio-Rad, USA) and imaged with a ChemiDoc MP imaging system. Antibody information including clone numbers and dilutions is summarized in **Table S2**.

**Quantitative Real-time PCR.**

To validate the expression of immune-related genes, qRT-PCR was performed using RNA extracted from DC2.4 cells cultured in biofilm-conditioned media. Mature *Staphylococcus aureus* biofilms were first treated with PBS, MnO₂, PMS, or MnO₂@PMS under 808 nm NIR irradiation (1 W/cm², 5 min), followed by incubation at 37 °C overnight. Supernatants were collected, filtered through 0.22 μm membranes, and diluted 1:4 in complete culture medium. DC2.4 cells were seeded in 6-well plates and cultured in the conditioned media for 24 h. After incubation, cells were washed with PBS, and total RNA was extracted using TRIzol reagent (Invitrogen, USA) according to the manufacturer’s protocol.

First-strand cDNA synthesis was performed using HiScript III RT SuperMix for qPCR (Vazyme, China). Quantitative PCR was conducted using ChamQ SYBR qPCR Master Mix (Vazyme, China) on a CFX96 Real-Time PCR Detection System (Bio-Rad, USA). Gene-specific primers were synthesized by Biosune Biotechnology Co., Ltd. (Shanghai, China). β-actin was used as the internal control, and relative gene expression levels were calculated using the 2⁻ΔΔCt method. For cGAS–STING activation assays under exogenous dsDNA stimulation (Figure 5I), DC2.4 cells were seeded in 6-well plates and, after overnight adherence, were treated with MnO₂@PMS alone, dsDNA alone, or the combination of MnO₂@PMS and dsDNA for a defined stimulation period before RNA extraction. Total RNA isolation, cDNA synthesis, and RT–qPCR analysis of Ifnb1 and Cxcl10 were performed as described above, and relative expression levels were calculated using the 2⁻ΔΔCt method with β-actin as the internal control. Primer sequences are provided in **Table S3**.

**Histological Evaluation of Major Organs.**

These assays were designed to evaluate short-term systemic toxicity under the present dosing regimen; longer-term biodistribution and chronic exposure safety were not addressed in this study. To assess potential systemic toxicity associated with nanomaterial treatment, major organs including heart, liver, spleen, lung, and kidney were harvested from treated mice on day 14 post-intervention. Organs were fixed in 4% paraformaldehyde, embedded in paraffin, sectioned at 5 μm thickness, and subjected to hematoxylin and eosin (H&E) staining following standard protocols. Histopathological features were examined under a light microscope (Leica DM3000, Germany) to identify signs of inflammatory infiltration, tissue degeneration, or architectural disruption.

***In vivo* Antibacterial and Antibiofilm Evaluation.**

To assess the *in vivo* therapeutic efficacy of MnO₂@PMS nanocomposites, a subcutaneous implant-associated infection model was established using male BALB/c mice (6–8 weeks old, SPF grade). Sterile round titanium discs (diameter: 1 cm) were surgically implanted into the dorsal subcutaneous space under isoflurane anesthesia. Immediately after implantation, methicillin-resistant *Staphylococcus aureus* (MRSA; 1 × 10⁷ CFU/mL in 100 μL PBS) was injected locally at the implant site to induce infection.

Mice were randomly assigned to four groups (n = 5 per group) using a simple random-number table: PBS, MnO₂, PMS, and MnO₂@PMS. The sample size was chosen based on previously published subcutaneous biofilm infection studies and feasibility considerations for this exploratory preclinical evaluation, and no formal a priori power calculation was performed. On Day 0 (surgery day), a single-dose local injection of the respective treatment material (200 μL, 50 μg/mL in PBS) was administered per group. For the MnO₂@PMS group, near-infrared (NIR) laser irradiation (808 nm, 1 W/cm², 5 min) was applied immediately post-injection on Day 0 and repeated once on Day 1 to facilitate photothermal and photosensitizer activation.

Disease progression was monitored by capturing standardized images of the erythematous and swollen region surrounding the implant on Days 0, 2, 4, 6, 8, 10, 12, and 14. Lesion area was quantified using ImageJ software based on surface area measurements. Only mice that underwent successful implantation and infection induction were included. Animals that died prematurely, exhibited unrelated health issues, or failed to establish infection were excluded from analysis.

On Day 14, mice were euthanized, and titanium discs along with surrounding subcutaneous tissues were collected for comprehensive assessment. Peri-implant tissues were homogenized in sterile PBS, serially diluted, and plated on LB agar to determine bacterial burden via colony-forming unit (CFU) counting. For biofilm visualization, the explanted titanium discs were stained using the LIVE/DEAD™ BacLight Bacterial Viability Kit (SYTO9/PI, ThermoFisher Scientific) and imaged via confocal laser scanning microscopy (Leica TCS SP8, Germany). Biofilm morphology and structural integrity were further examined by scanning electron microscopy (SEM) following fixation in 2.5% glutaraldehyde, graded ethanol dehydration, and critical point drying.

All animal experiments were conducted in accordance with institutional and national ethical guidelines, and were approved by the Animal Ethics Committee of Shanghai Chengxi Biotechnology Co., Ltd. (approval number: CX052503073). Quantitative analyses of CFU counts, biofilm thickness and histological or immunofluorescence staining were performed by investigators who were blinded to group allocation, whereas treatment administration was not blinded due to the obvious differences in injection composition and laser irradiation.

**Histological and Immunofluorescence Analysis.**

On Day 14 post-treatment, peri-implant subcutaneous tissues were harvested and subjected to histological and immunofluorescence analysis. All procedures, including fixation, embedding, sectioning, and staining, were outsourced to Shanghai Voden Biotechnology Co., Ltd., following the company's standardized protocols and prior sample collection guidelines.

For histological evaluation, tissues were fixed in 4% paraformaldehyde, embedded in paraffin, and sectioned at 5 μm thickness. Hematoxylin and eosin (H&E) staining was performed to assess tissue architecture, inflammatory cell infiltration, and granulation tissue formation.

Adjacent samples were embedded in optimal cutting temperature (OCT) compound and cryosectioned at 6–8 μm for immunofluorescence staining. Antibodies targeting CD31 and α-SMA were used to evaluate neovascularization and perivascular structures, while IL-6 and MPO staining assessed inflammatory cytokine expression and neutrophil infiltration. HIF-1α staining was conducted to examine tissue hypoxia. DAPI was used for nuclear counterstaining. Images were acquired using a confocal laser scanning microscope (Leica TCS SP8, Germany).

Quantitative analysis of vessel density, inflammatory marker expression, and biofilm thickness was conducted using ImageJ software, analyzing multiple high-power fields per sample to ensure representative evaluation. All staining experiments included appropriate negative and isotype controls to validate specificity.

***In vivo* Flow Cytometry Analysis.**

To assess local immune reprogramming following treatment, flow cytometric analysis was performed on T cells and dendritic cells (DCs) isolated from inguinal lymph nodes. At Day 14 post-treatment, lymph nodes were aseptically harvested and gently dissociated through a 70 μm cell strainer using RPMI-1640 medium supplemented with 2% fetal bovine serum (FBS). Single-cell suspensions were centrifuged and resuspended in PBS containing 2% FBS.

To exclude dead cells, a LIVE/DEAD™ Fixable Dead Cell Stain Kit (Thermo Fisher Scientific, USA) was used according to the manufacturer’s instructions. After viability staining (15 min, room temperature, dark), cells were washed and incubated with anti-mouse CD16/32 antibody (clone 93, BioLegend, USA) for 10 min on ice to block Fc receptors and minimize nonspecific binding.

Subsequently, cells were stained with fluorophore-conjugated antibodies for surface markers. T cell subsets were identified using anti-CD3, anti-CD4, and anti-CD8 antibodies. For DC analysis, CD11c⁺ cells were gated and further profiled for maturation (CD80, CD86), migration (CCR7), and polarization (CD206) markers.

Samples were acquired using a BD LSRFortessa™ flow cytometer (BD Biosciences, USA), and data were analyzed using FlowJo v10.8 software.

As the primary site of antigen presentation and T cell–DC interaction, lymph nodes provide a representative tissue source for evaluating treatment-induced immune modulation. A complete list of fluorophore-conjugated antibodies and reagents is provided in Supplementary **Table S1.**

**Statistical Analysis**

All experiments were performed with at least three independent biological replicates, and technical replicates were included where appropriate. Data are presented as mean ± standard deviation (SD) unless otherwise indicated. Statistical comparisons were carried out using one-way analysis of variance (ANOVA) followed by Tukey’s post hoc test or Student’s t-test, as appropriate for the dataset. Analyses and figure generation were performed using GraphPad Prism 9.0 (GraphPad Software, USA) and R software (version 4.3.0; R Foundation for Statistical Computing, Vienna, Austria). A p-value < 0.05 was considered statistically significant.

**Supporting Figures**

**
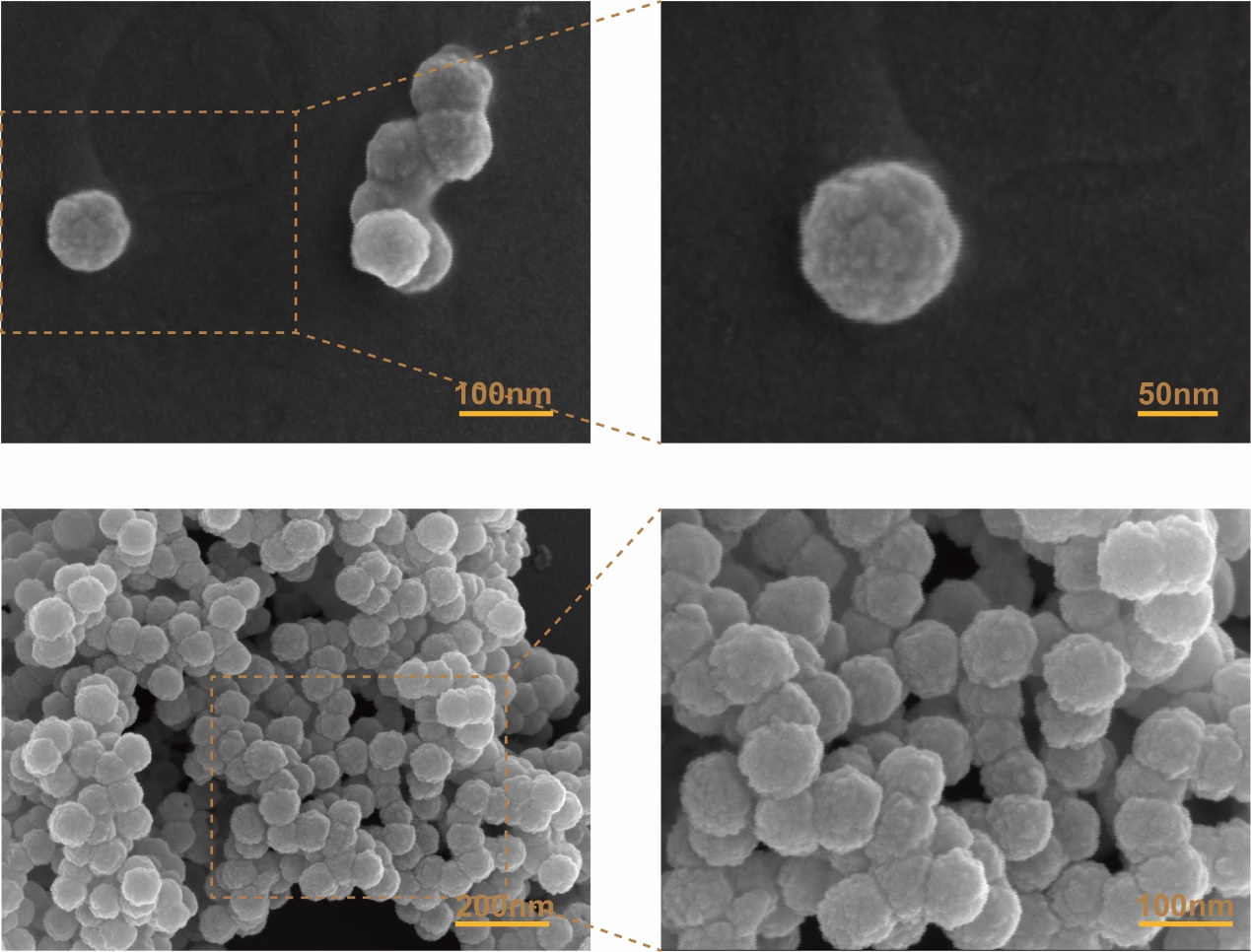
**

**Figure S1.** Scanning electron microscopy (SEM) images of hollow MnO₂ nanoparticles. The particles exhibit a uniform spherical morphology with a rough surface texture and hollow interior. High-magnification images (top right and bottom right) highlight the detailed surface architecture and hollow structure of individual nanoparticles. Scale bars: 200 nm (bottom left), 100 nm (top left and bottom right), 50 nm (top right).

**
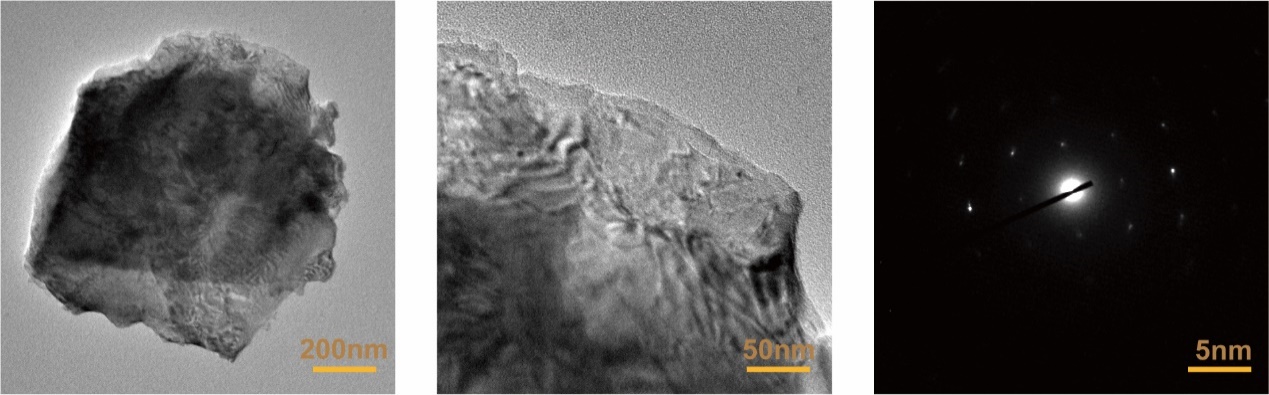
**

**Figure S2.** TEM characterization of the synthesized material. (Left) Low-magnification TEM image reveals the overall morphology of the nanosheet-like structure. (Middle) High-resolution TEM (HRTEM) image highlights the layered texture and edge details, indicating partial lattice distortion. (Right) Selected area electron diffraction (SAED) pattern demonstrates distinct diffraction spots arranged in concentric rings. Scale bars: 200 nm (left), 50 nm (middle), 5 nm⁻¹ (right).

**
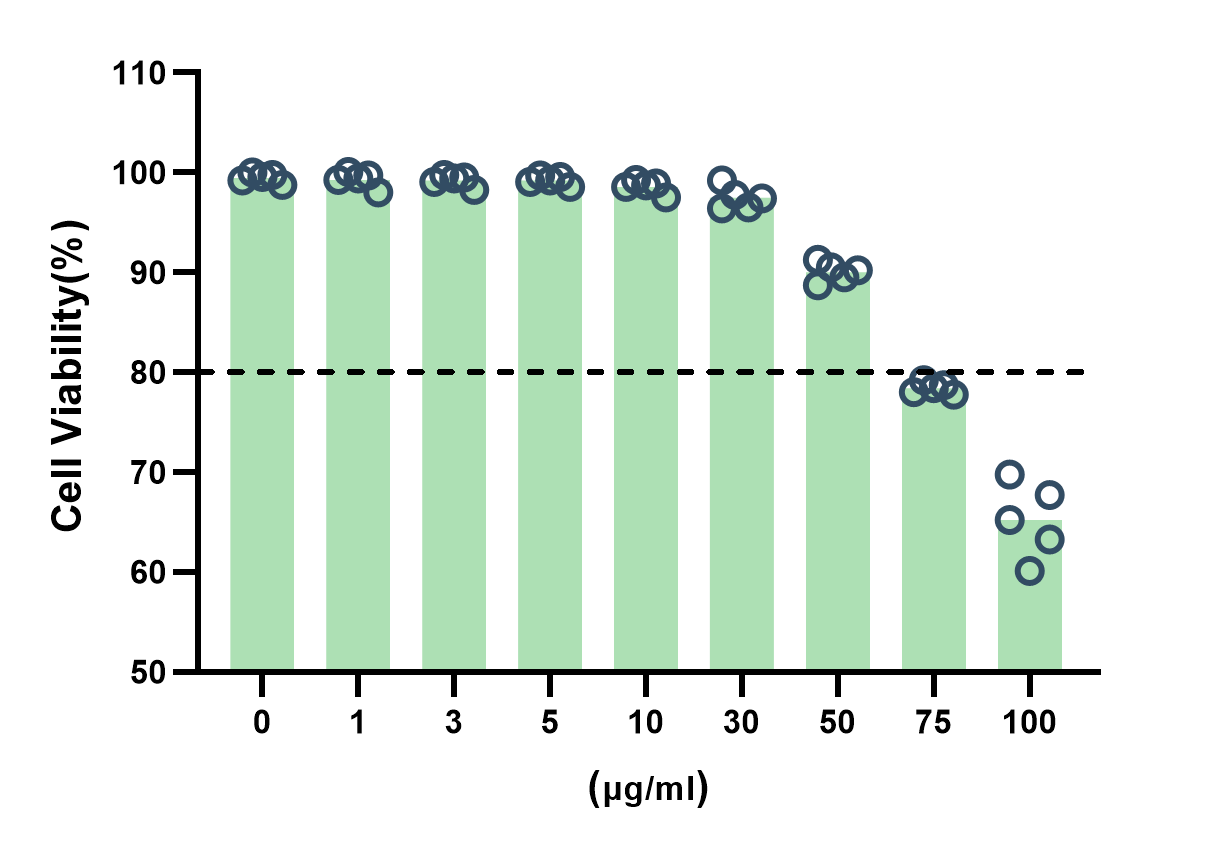
**

**Figure S3.** The cellular viability of DC2.4 cells treated with different concentrations of MnO2@PMS NPs (0, 1, 3, 5, 10, 30, 50, 75, 100 μg/ml) (n=5).

| **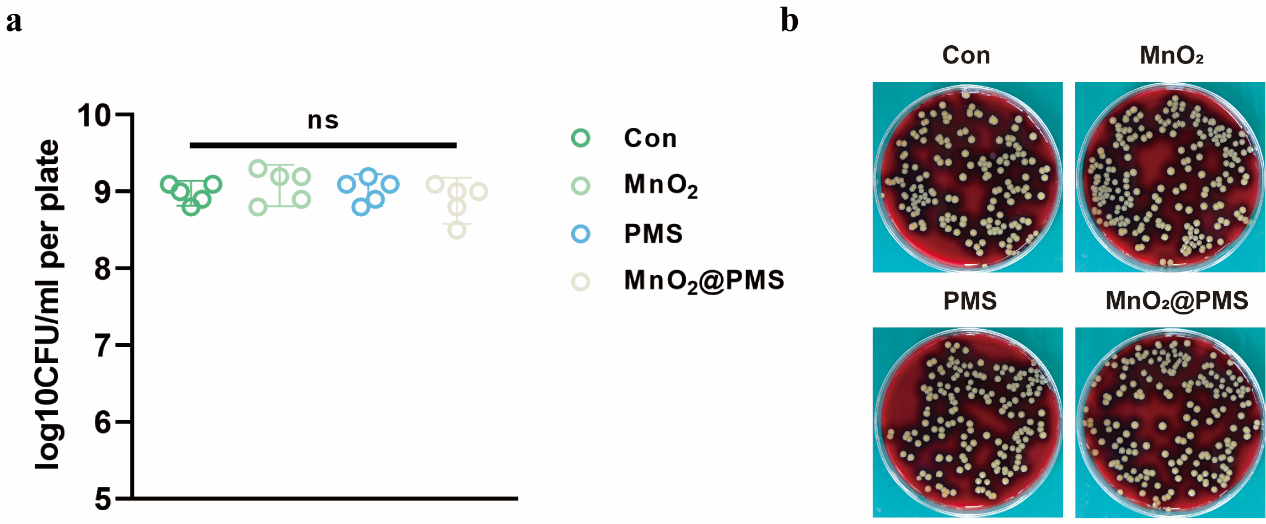** |
| --- |
| **Figure S4.** (a, b) S. aureus (1 x 10^6^ CFU/ml) was co-cultured with different NPs (MnO2, PMS, MnO2@PMS NPs, final concentration 50μg/ml) for 24h without 808nm laser irradiation, and the CFU counting results (a) and corresponding representative plate images (b) of each group were obtained after 24h. Each column shows the mean ± SD of five independent experiments. |
| 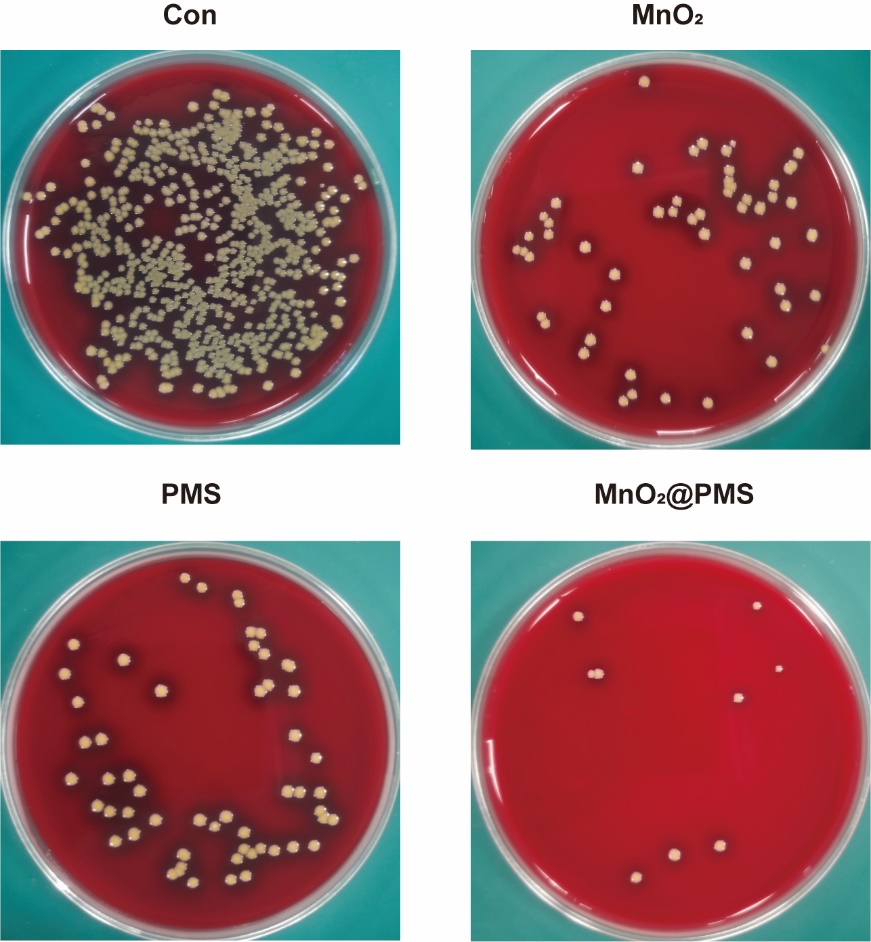 |
| **Figure S5.** S. aureus (1 x 10^6^ CFU/ml) was co-cultured with different NPs (MnO2, PMS, MnO2@PMS NPs, final concentration 50μg/ml) for 24h with 808nm laser irradiation, and the corresponding representative plate images of each group were obtained after 24h. |


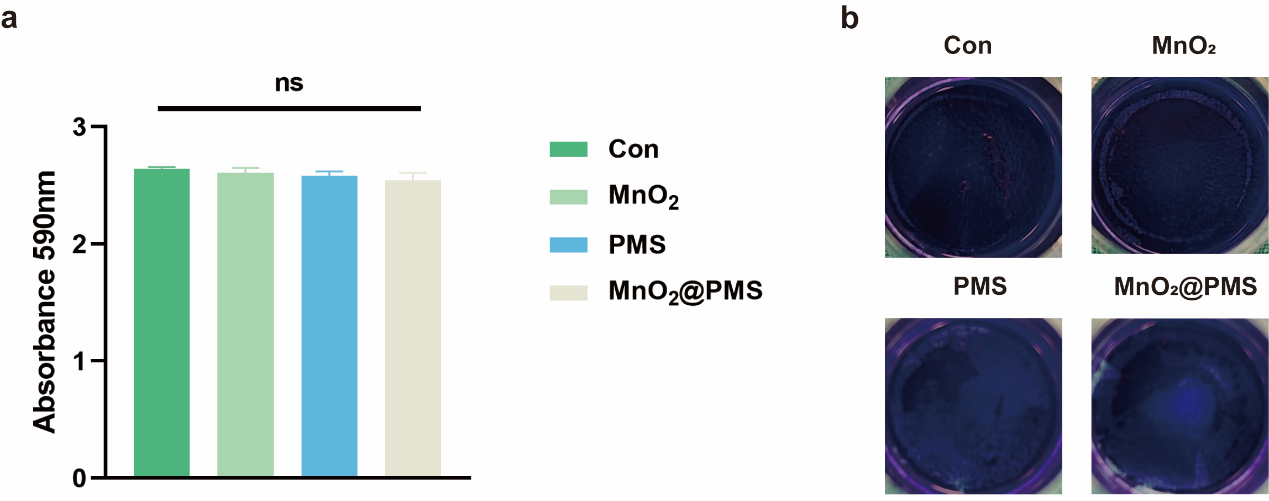


**Figure S6.** (a, b) *S. aureus* (1 × 10⁶ CFU/ml) was co-cultured with different NPs (MnO₂, PMS, MnO₂@PMS NPs; final concentration: 50 μg/ml) for 24 h without 808 nm laser irradiation to allow biofilm formation. The absorbance-based quantification of crystal violet staining (a) and the corresponding representative images of stained biofilms (b) are shown. No significant differences in biofilm biomass were observed among groups. Each column represents the mean ± SD of five independent experiments.


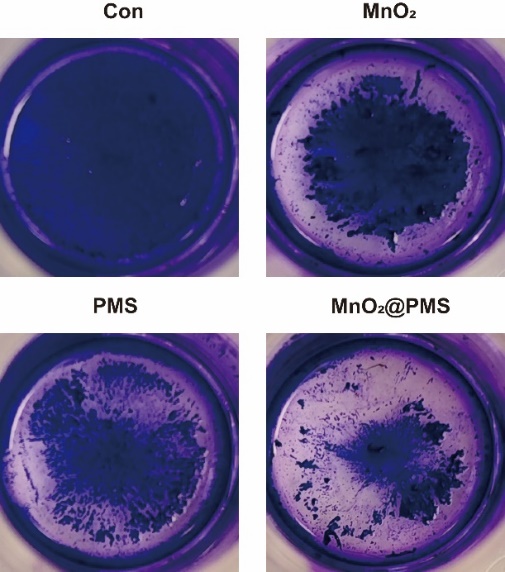


**Figure S7.** Representative crystal violet-stained images of *S. aureus* biofilms after overnight incubation. *S. aureus* (1 × 10⁶ CFU/ml) was cultured in the presence of different formulations (MnO₂, PMS, or MnO₂@PMS NPs; final concentration: 50 μg/ml) for 24 hours to allow biofilm formation, followed by 808 nm laser irradiation. After incubation, biofilms were stained with crystal violet to visualize biomass.


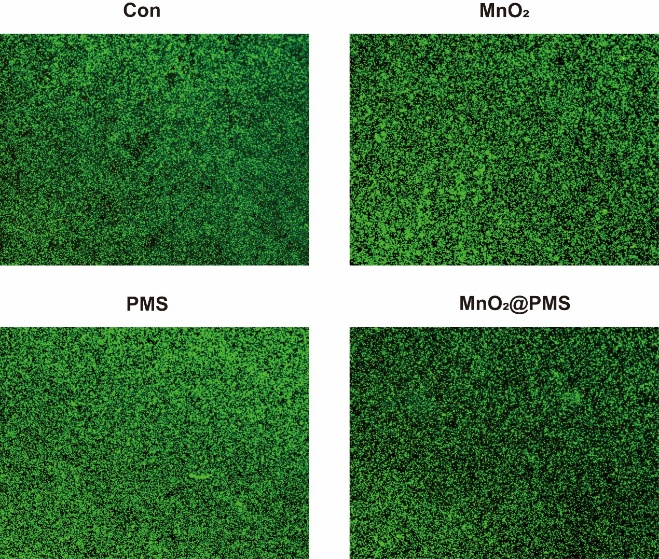


**Figure S8.** Live/dead fluorescence staining of *S. aureus* biofilms after 24 h co-culture with different NPs (MnO₂, PMS, MnO₂@PMS NPs; final concentration: 50 μg/ml) without 808 nm laser irradiation. Biofilms were stained with SYTO 9 (green, live bacteria) and propidium iodide (PI, red, dead bacteria), and imaged using a Leica DM8 fluorescence microscope.


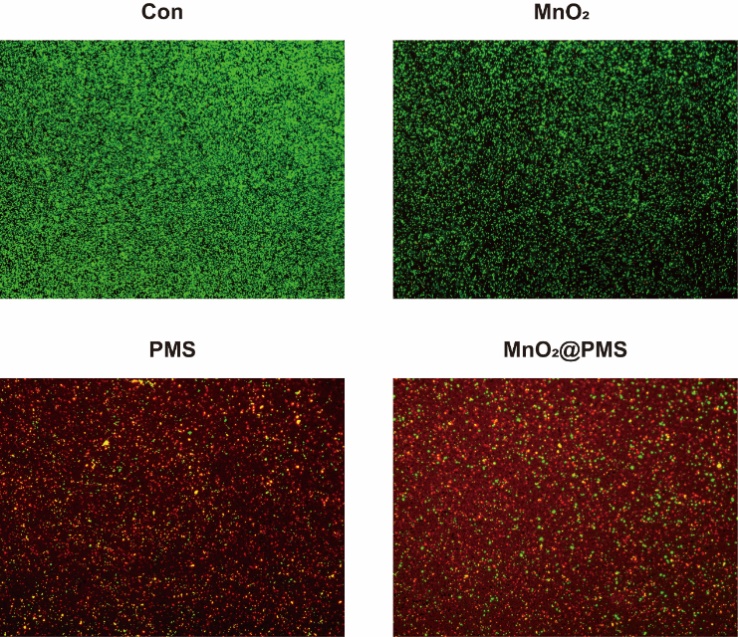


**Figure S9.** Live/dead fluorescence staining of *S. aureus* biofilms after 24 h co-culture with different NPs (MnO₂, PMS, MnO₂@PMS NPs; final concentration: 50 μg/ml) under 808 nm laser irradiation. Bacteria were stained with SYTO 9 (green, live) and propidium iodide (PI, red, dead), and imaged using a Leica DM8 fluorescence microscope.


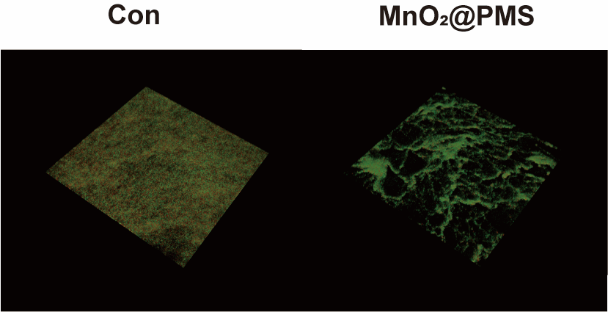


**Figure S10.** Representative 3D CLSM images of *S. aureus* biofilms stained with SYTO9 (green, total biomass) and TOTO-3 (red, extracellular DNA) after 808 nm laser irradiation.


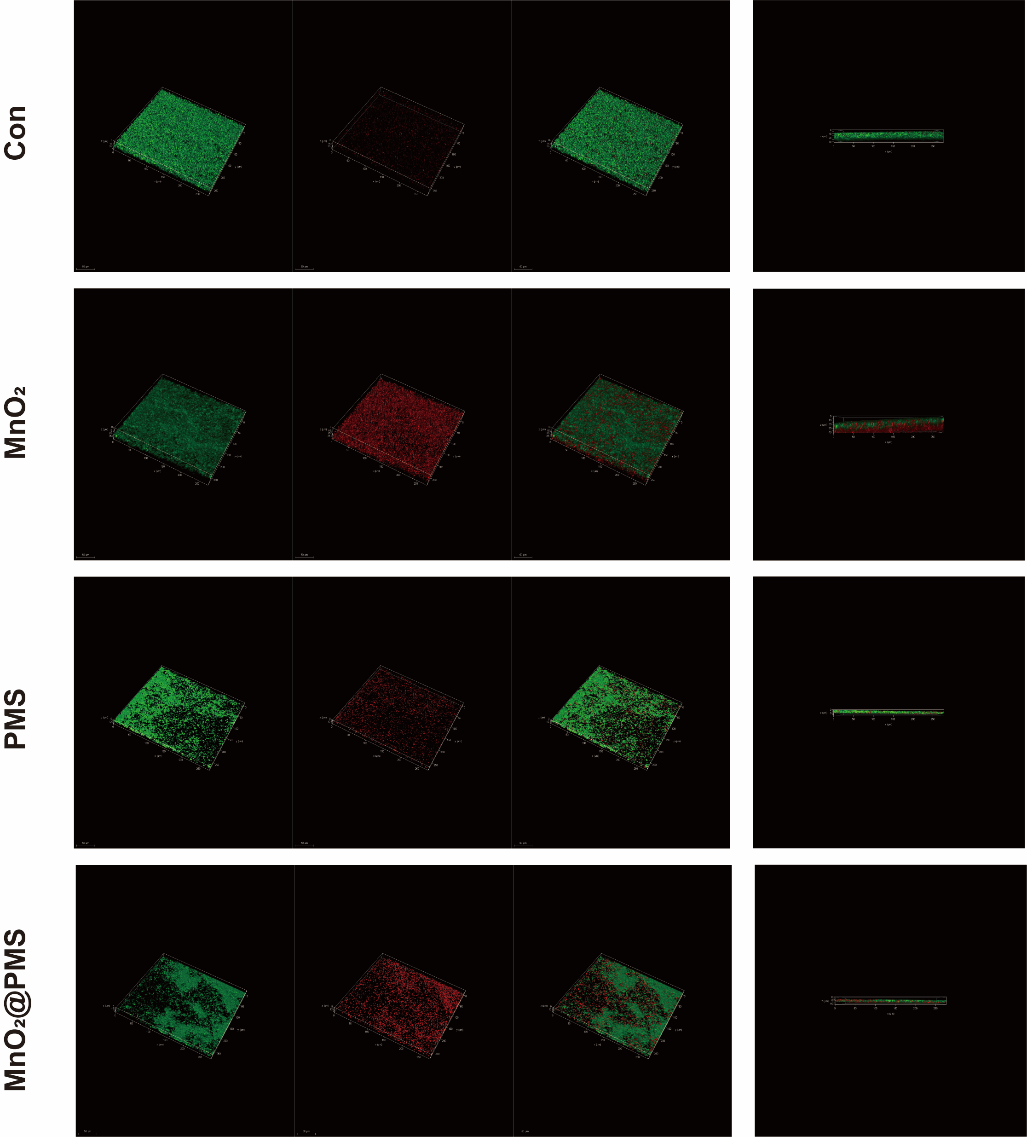


**Figure S11.** 3D CLSM images of *S. aureus* biofilms after 24 h treatment with MnO₂, PMS, or MnO₂@PMS NPs (50 μg/mL) under 808 nm laser irradiation. Biofilms were stained with SYTO9 (green, live) and PI (red, dead).


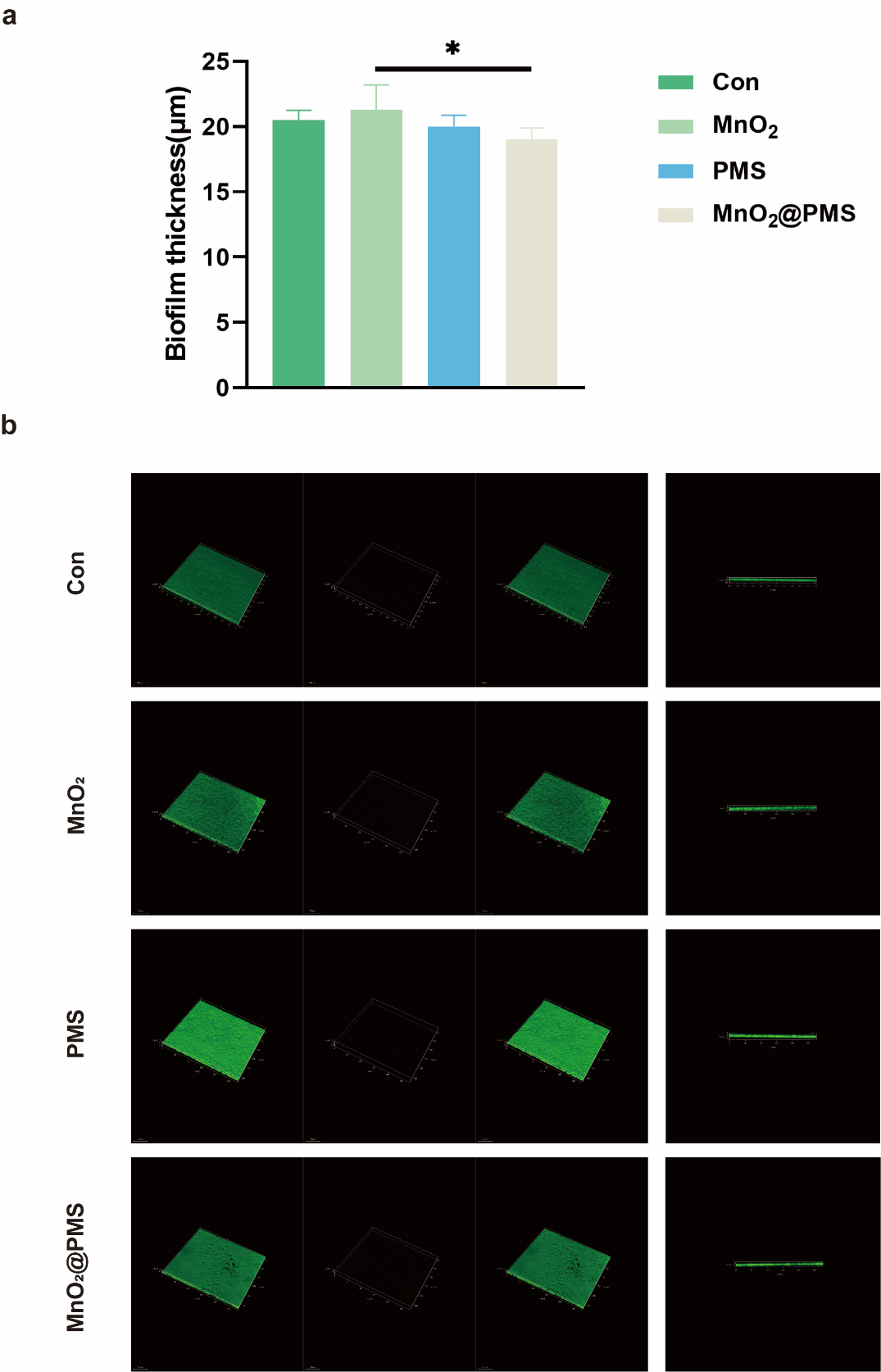


**Figure S12. (a, b)** CLSM analysis of *S. aureus* biofilms after 24 h co-incubation with MnO₂, PMS, or MnO₂@PMS NPs (50 μg/mL) under dark conditions (no 808 nm irradiation).
**(a)** Biofilm thickness quantification (Z-stack). Data represent mean ± SD (n = 3).
**(b)** Representative 3D CLSM reconstructions (top and side views) showing comparable biofilm structure across groups.


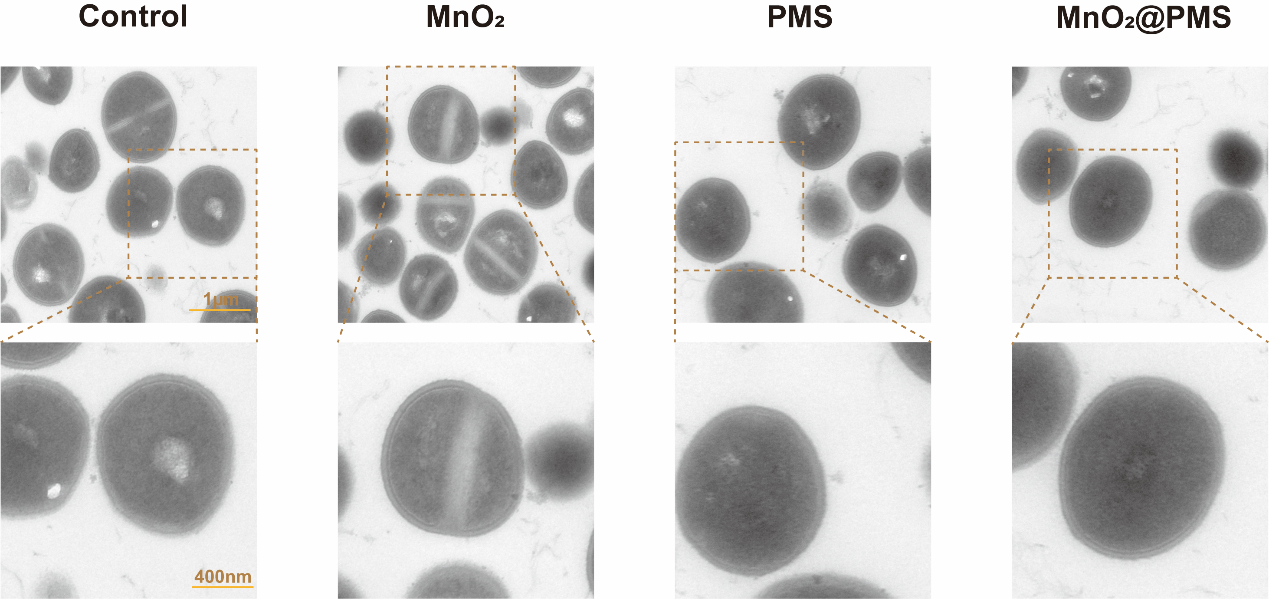


**Figure S13.** TEM images of *S. aureus* after 24 h incubation with MnO₂, PMS, or MnO₂@PMS NPs (50 μg/mL) under dark conditions. Insets: magnified views. Scale bars: 1 μm (main), 400 nm (insets).


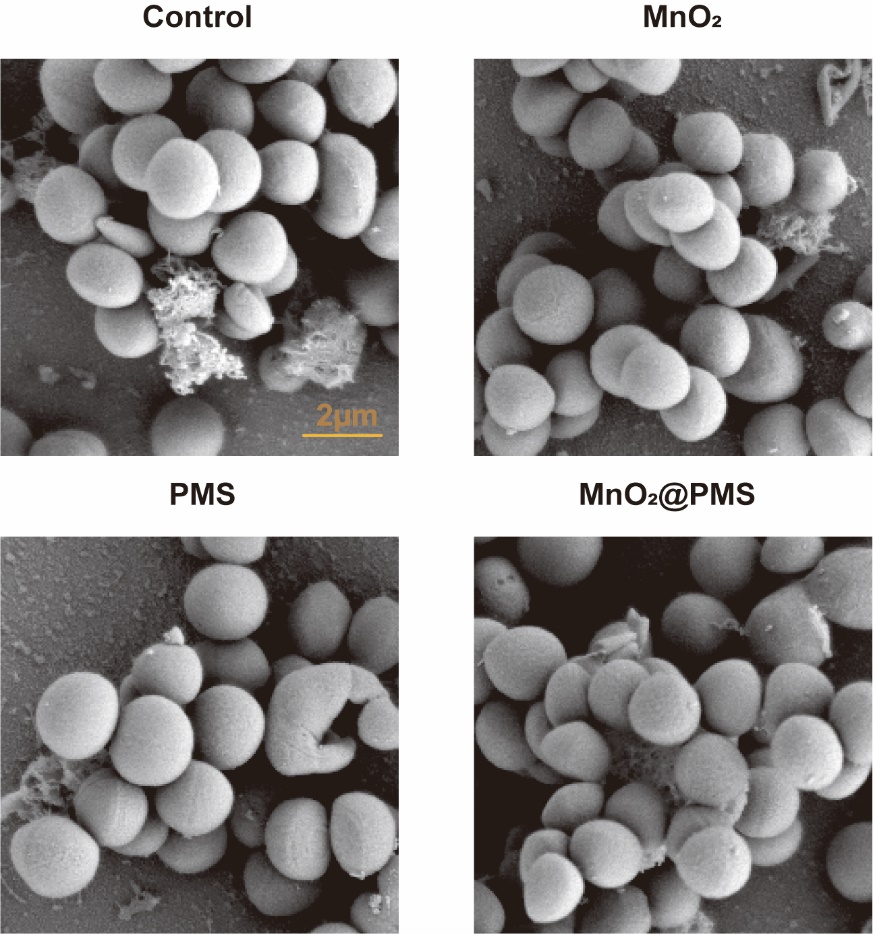


**Figure S14.** SEM images of *S. aureus* after 24 h co-culture with MnO₂, PMS, or MnO₂@PMS NPs (50 μg/mL) under dark conditions. Scale bar: 2 μm.


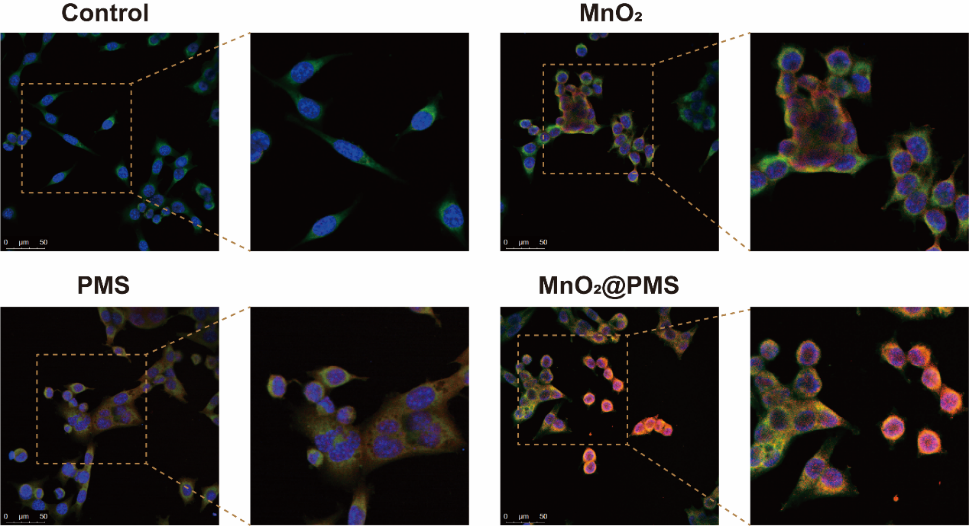


**Figure S15.** Representative immunofluorescence images of DC2.4 cells treated with MnO₂, PMS, or MnO₂@PMS NPs (50 μg/mL) in the presence of exogenous dsDNA, without biofilm supernatant or laser irradiation. Cells were stained with DAPI (blue), CD206 (green), and CD40 (red) to assess dendritic cell activation.


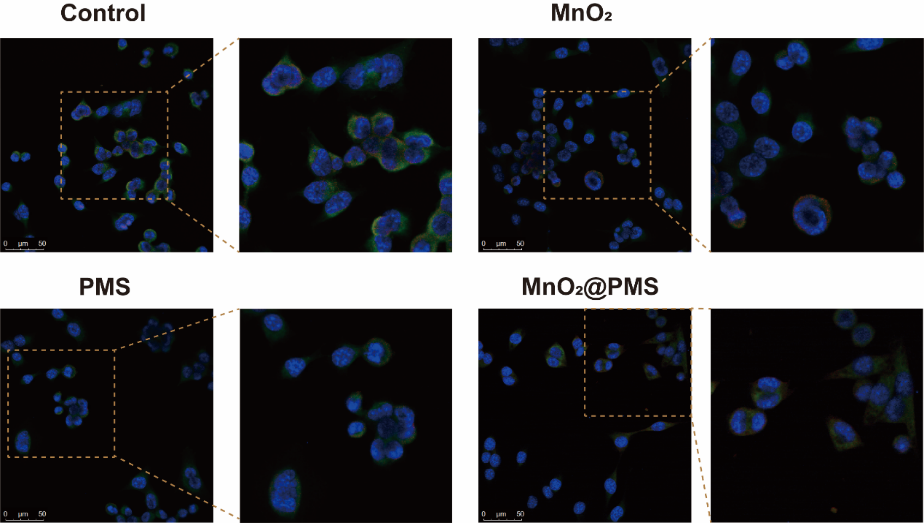


**Figure S16.** Immunofluorescence images of DC2.4 cells after 24 h treatment with MnO₂, PMS, or MnO₂@PMS NPs (50 μg/mL) in the presence of bacterial biofilm supernatant, without 808 nm irradiation. Cells were stained with DAPI (blue), CD206 (green), and CD40 (red).

**
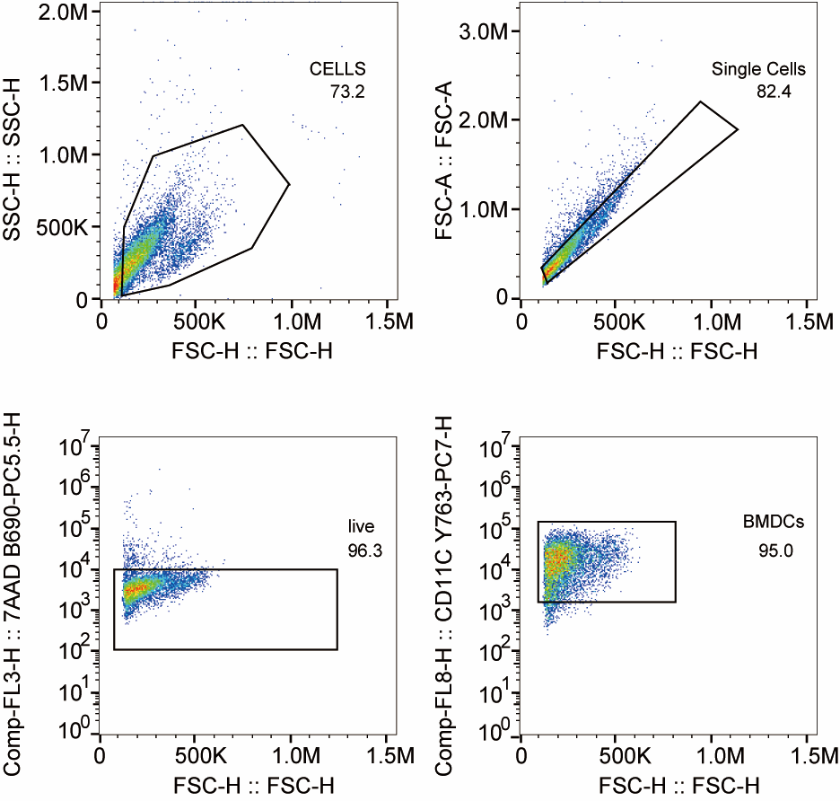
**

**Figure S17.** Flow cytometry gating strategy for bone marrow-derived dendritic cells (BMDCs). Cells were gated by FSC/SSC, followed by doublet exclusion (FSC-A vs. FSC-H), viability selection (7-AAD⁻), and identification of CD11c⁺ MHC II⁺ populations. Final purity reached 95.0%.

**
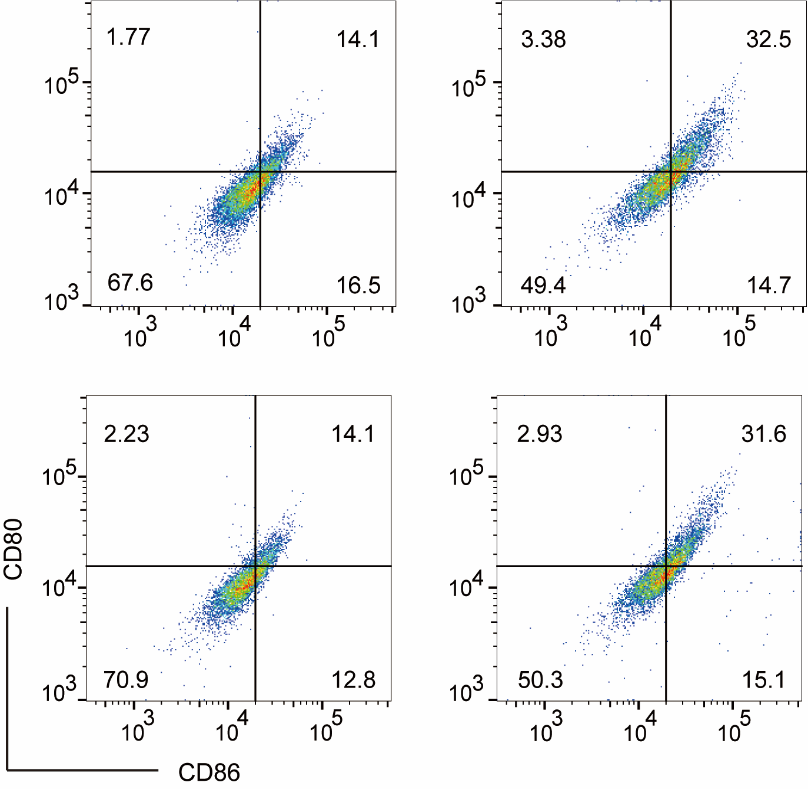
**

**Figure S18.** Flow cytometry analysis of CD80/CD86 expression in dsDNA-stimulated DC2.4 cells. Proportions of CD80⁺CD86⁺ (activated) and CD80⁻CD86⁺ (immature) subsets are shown to establish baseline immune responsiveness.

**
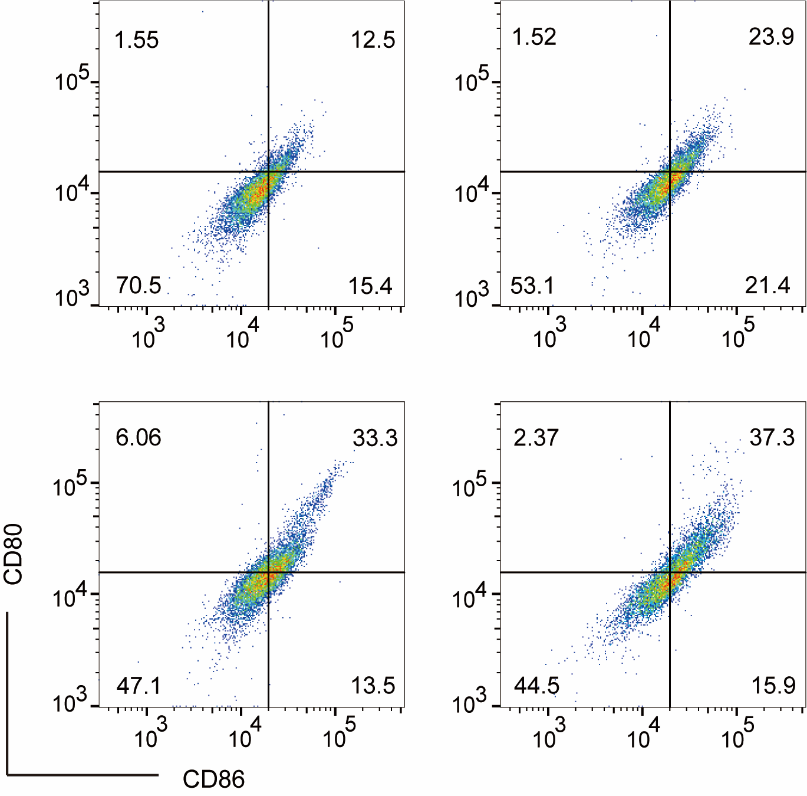
**

**Figure S19.** MnO₂@PMS rescues DC activation under biofilm suppression. Biofilm supernatant inhibited DC2.4 maturation, but MnO₂@PMS treatment restored CD80⁺CD86⁺ populations, confirming its immune-activating effect.

**
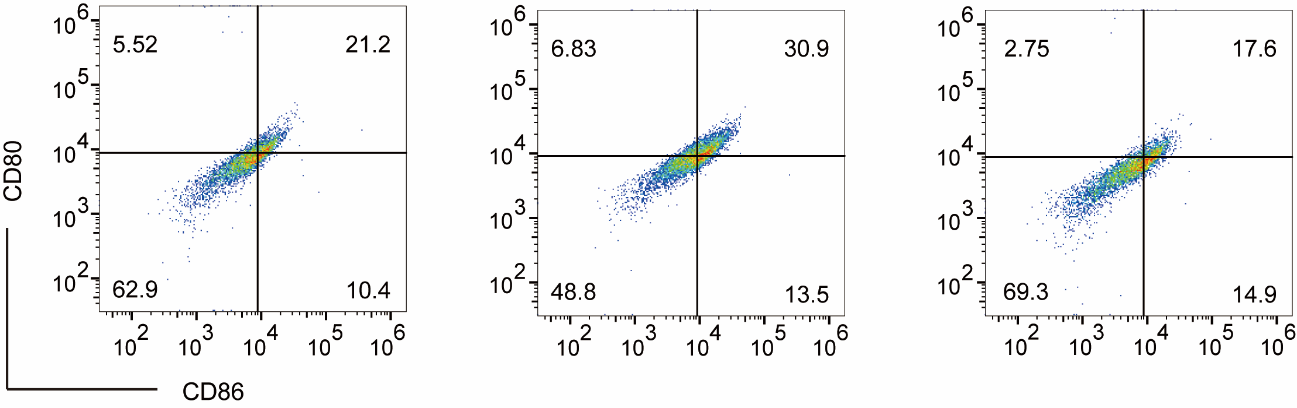
**

**Figure S20.** MnO₂@PMS enhances DC2.4 maturation, attenuated by glutamate. Flow cytometry shows MnO₂@PMS (50 μg/ml) increased CD80⁺CD86⁺ populations in dsDNA-stimulated DC2.4 cells, while glutamate co-treatment reversed this activation.


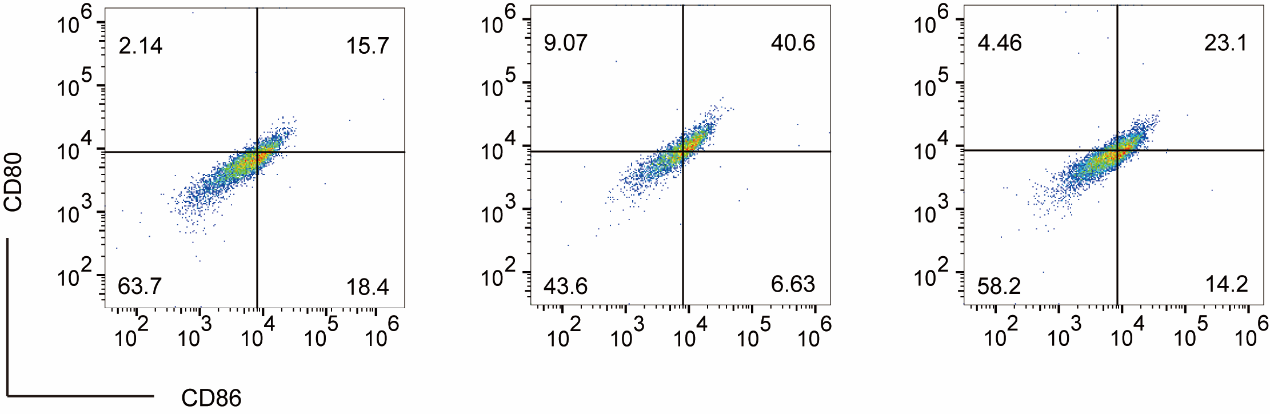


**Figure S21.** MnO₂@PMS overcomes biofilm suppression, blocked by glutamate. Biofilm supernatant inhibited DC2.4 maturation, but MnO₂@PMS restored CD80⁺CD86⁺ expression. Glutamate again abolished this rescue effect.

**
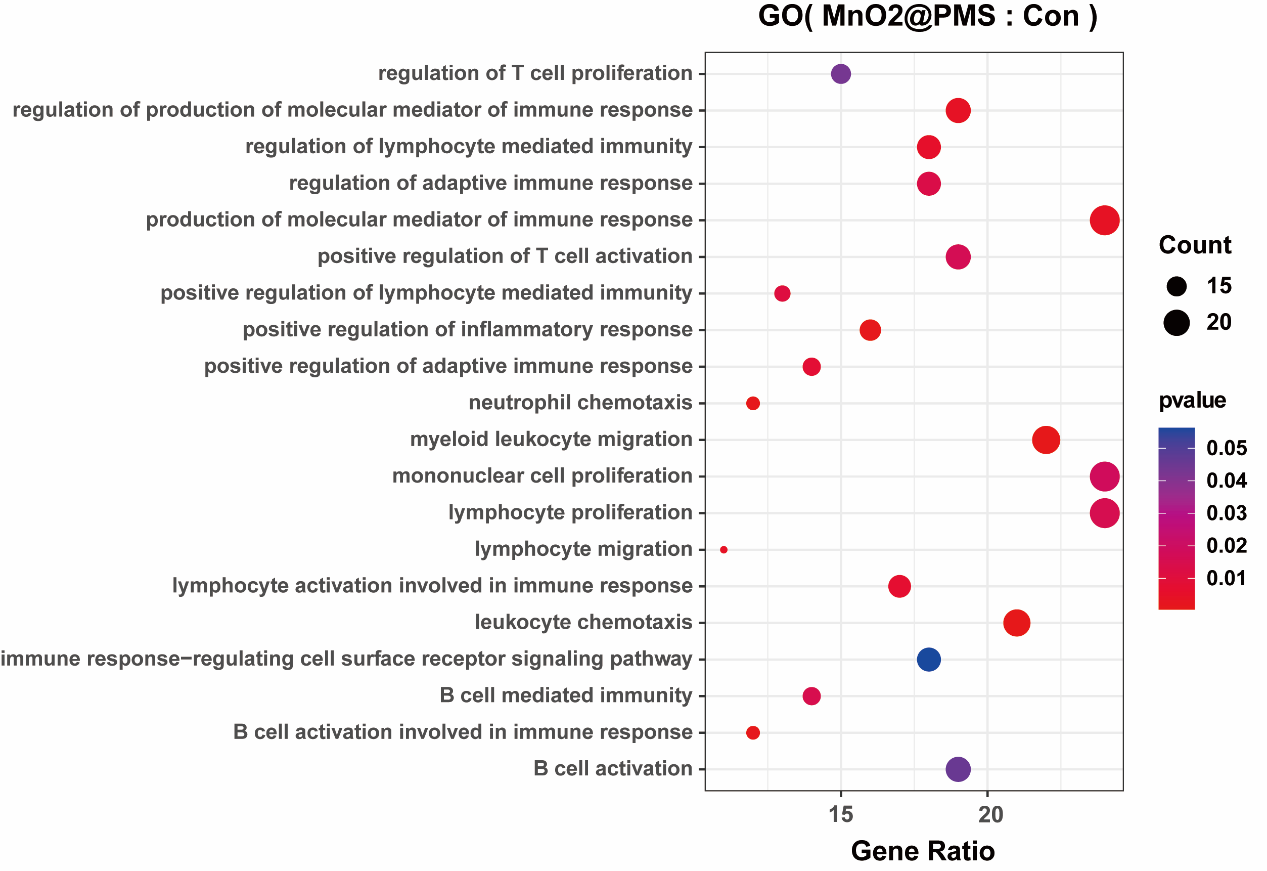
**

**Figure S22.** GO enrichment analysis of differentially expressed genes in the MnO₂@PMS group vs. control. Enriched biological processes include immune activation, lymphocyte proliferation, and leukocyte migration. Dot size represents gene count; color indicates adjusted *p*-value.

**
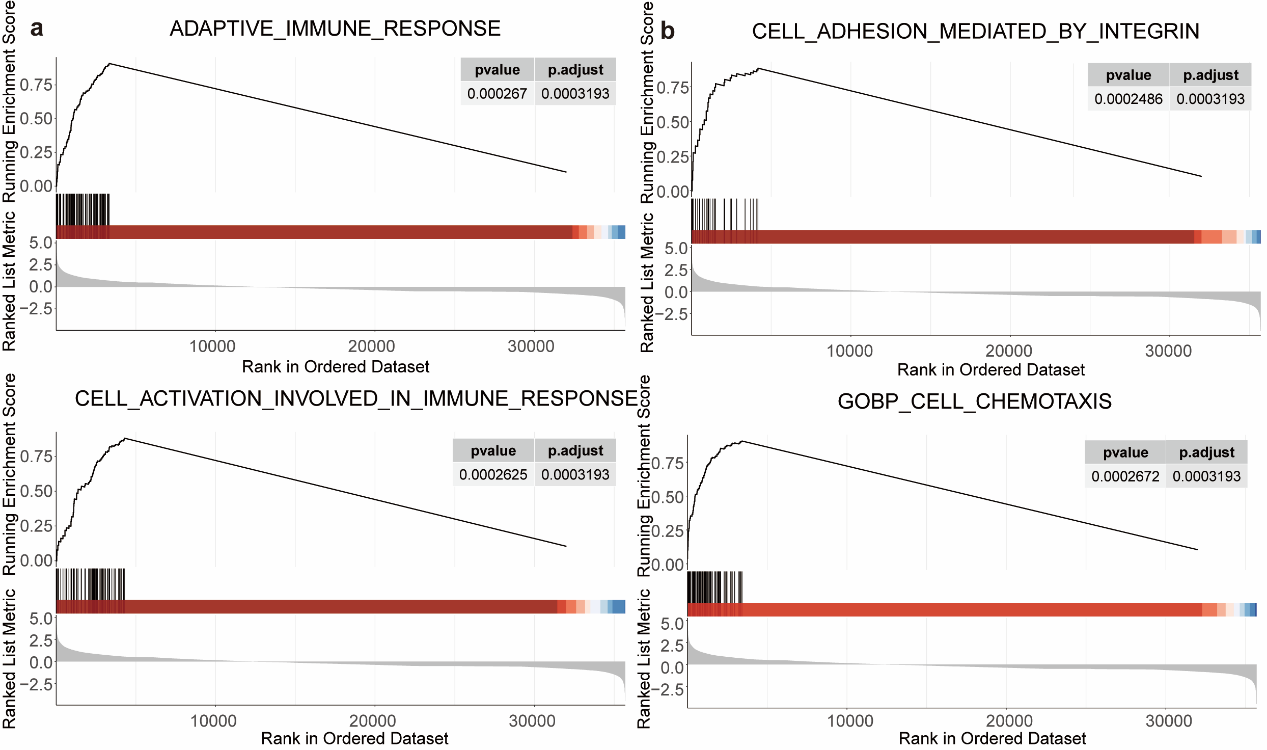
**

**Figure S23.** GSEA of immune-related pathways in the MnO₂@PMS+NIR group. **(a)** Positive enrichment of adaptive immune response and immune cell activation pathways.
**(b)** Upregulation of integrin-mediated adhesion and chemotaxis pathways. All pathways showed significant enrichment (FDR < 0.001).


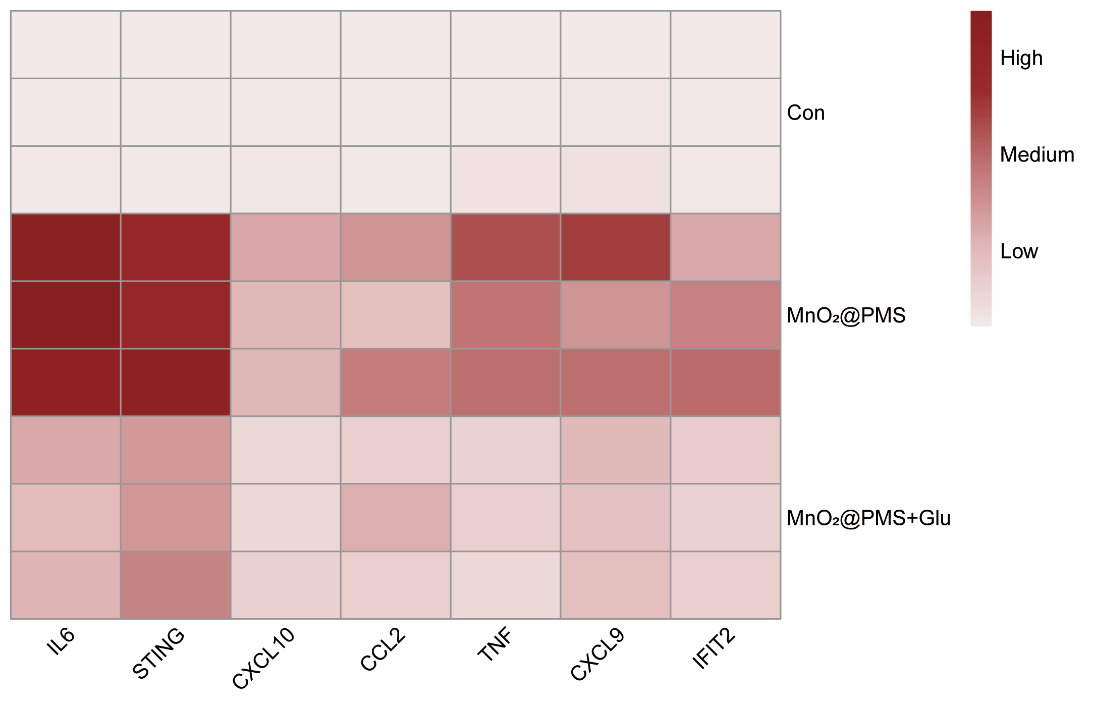


**Figure S24.** Heatmap of mRNA expression of inflammatory and immune-related genes in DCs after treatment with MnO₂@PMS (50 μg/mL) or MnO₂@PMS plus glutamate. qPCR analysis shows differential expression of IL6, STING, CXCL10, CCL2, TNF, CXCL9, and IFIT2 across groups. Control (Con) included for comparison.


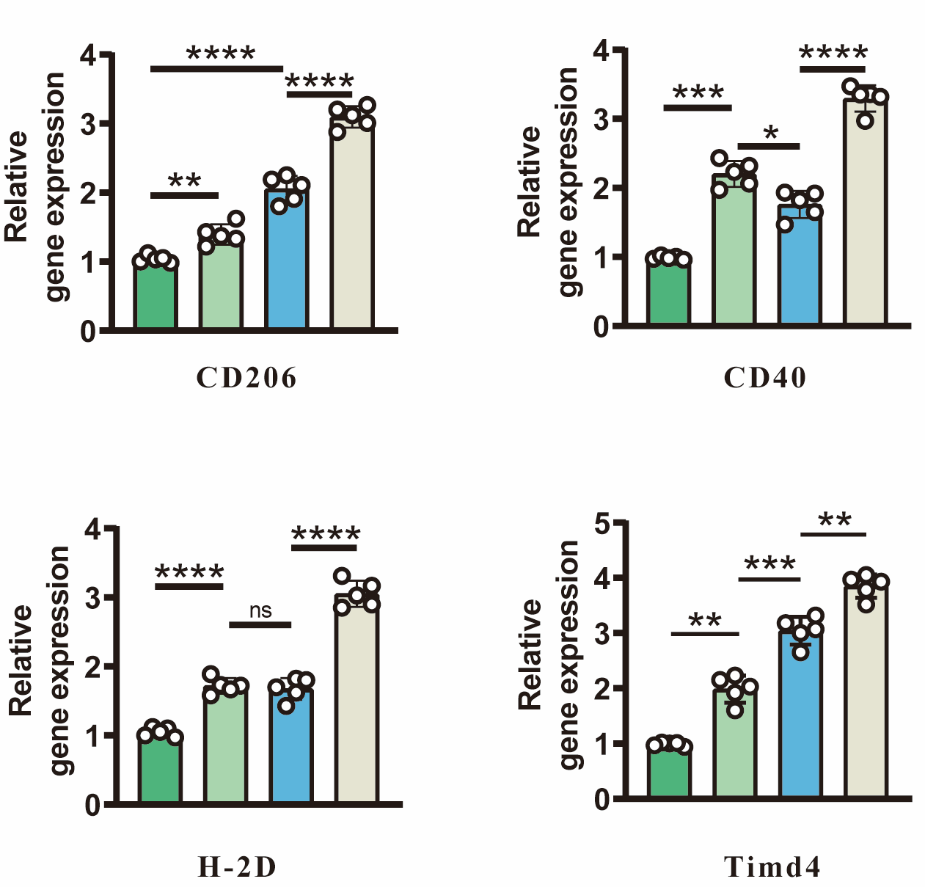


**Figure S25.** RT–qPCR analysis of dendritic-cell phenotype–associated genes in DC2.4 cells. Relative mRNA expression of CD206, CD40, H-2D and Timd4 in DC2.4 cells cultured with biofilm supernatants from the PBS, MnO₂, PMS or MnO₂@PMS treatment groups. Data are shown as mean ± SD (n = X). *P < 0.05, **P < 0.01, ***P < 0.001, ****P < 0.0001; ns, not significant.

**
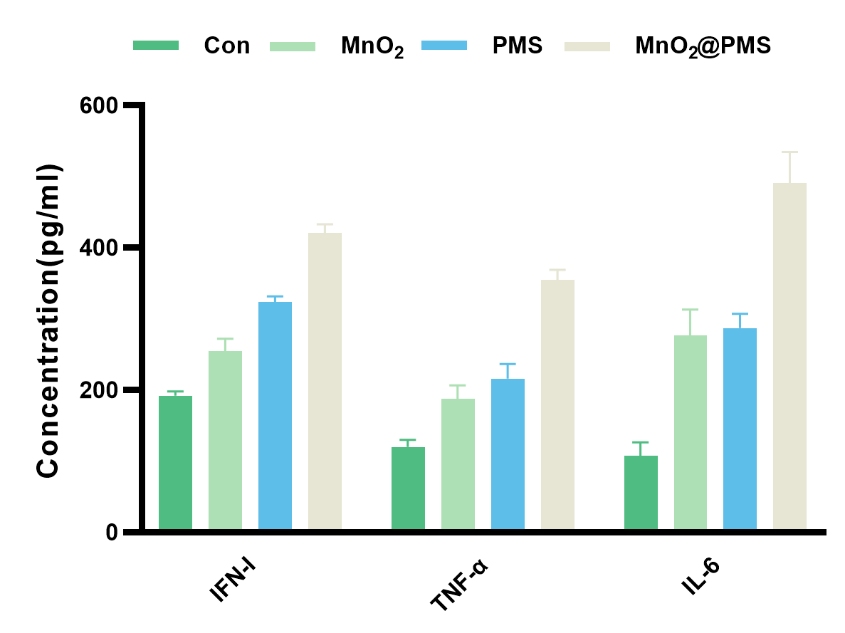
**

**Figure S26.** ELISA quantification of IL-6, TNF-α, and IFN-I secretion by DCs pre-exposed to *S. aureus* biofilm supernatant and treated with PBS, MnO₂, PMS, or MnO₂@PMS (50 μg/mL) under 808 nm irradiation.

**
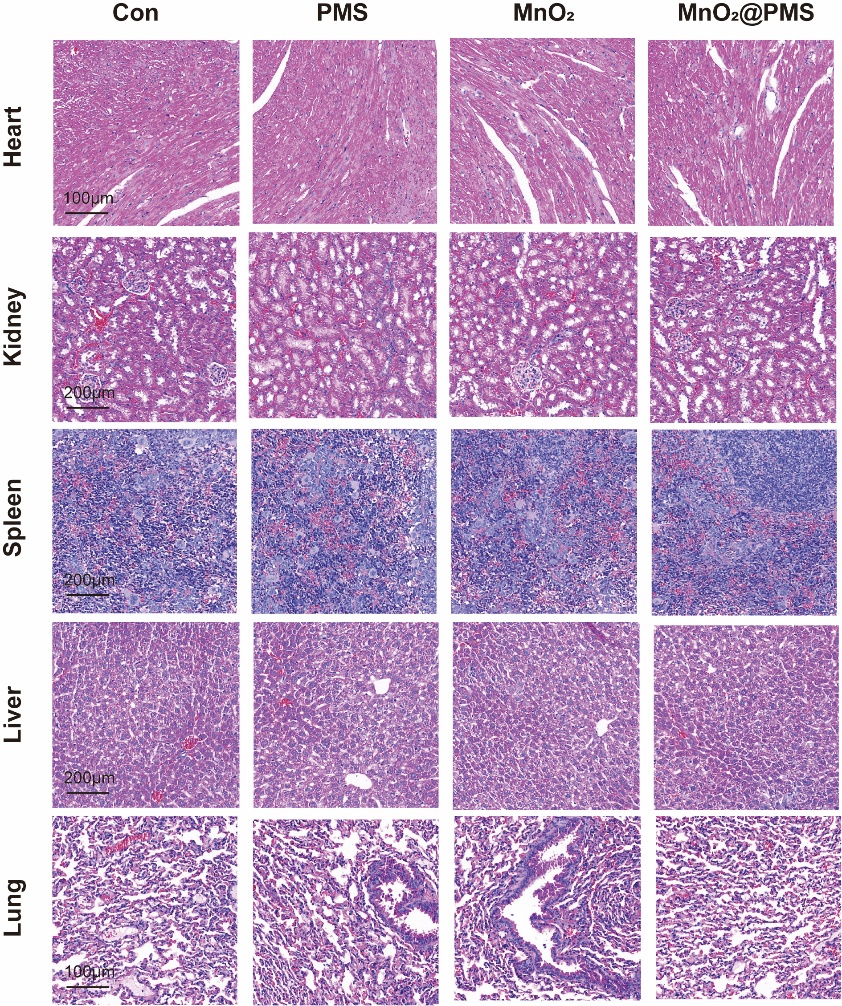
**

**Figure S27.** Hematoxylin and eosin (HE) staining of the major organs in the primary implant-associated infection mice model on day 14 after NPs and laser treatment. Scale bar: 100μm , 200 μm.

**
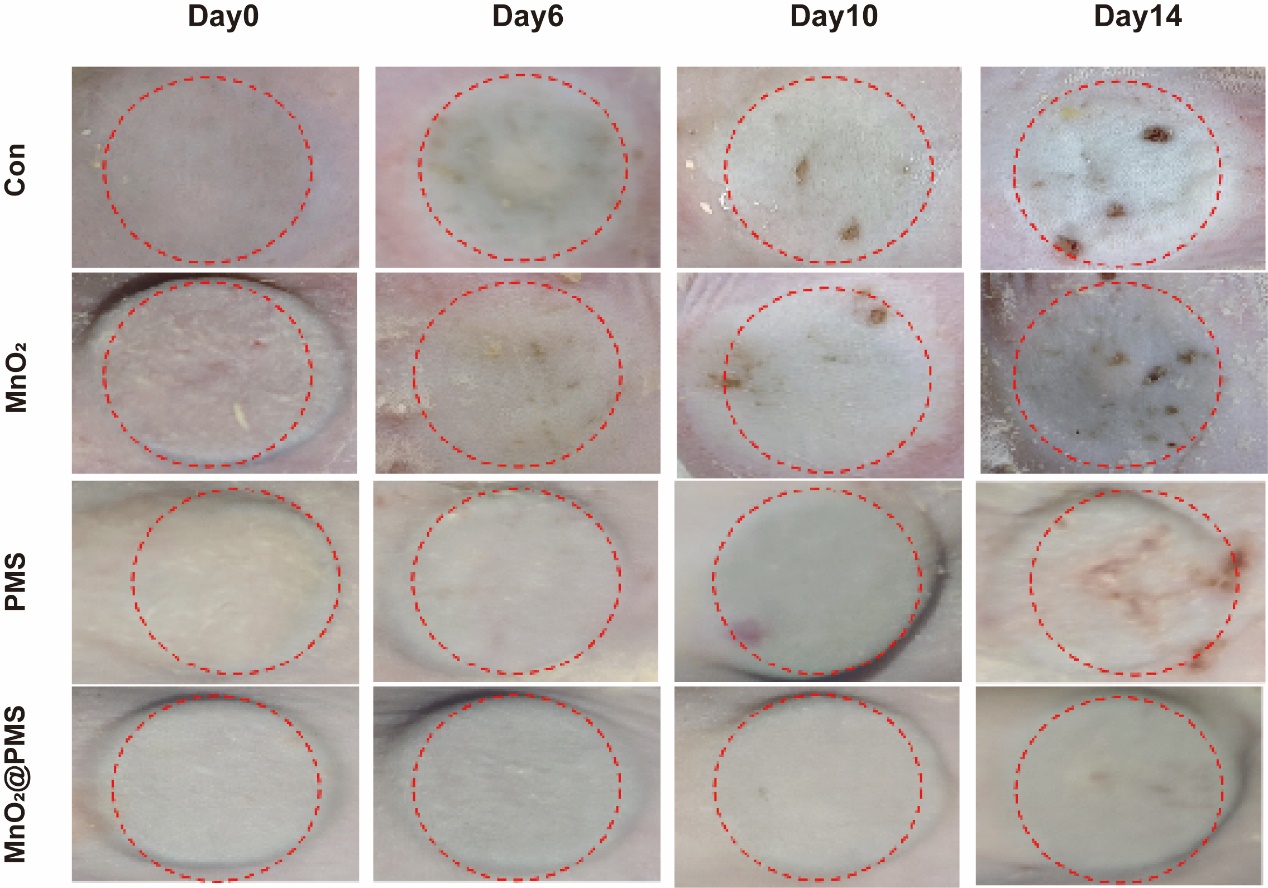
**

**Figure S28.** Representative macroscopic images of subcutaneous implants in infected mice at Days 0, 6, 10, and 14 after treatment with PBS, MnO₂, PMS, or MnO₂@PMS. Red dashed circles mark implant sites.

**
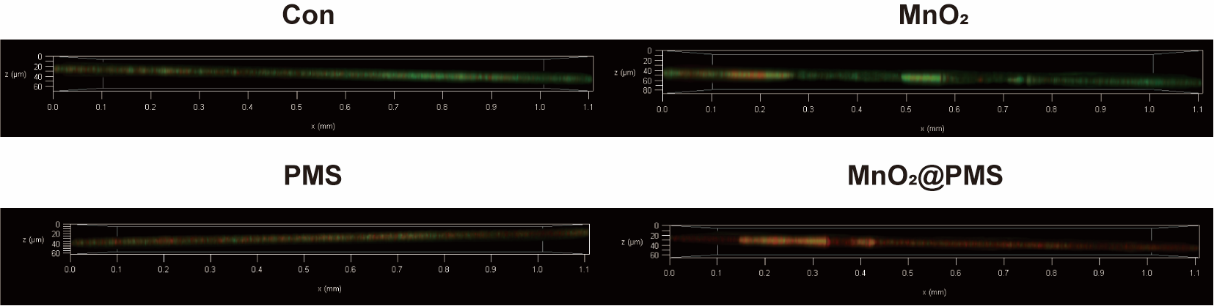
**

**Figure S29.** Z-stack confocal images of peri-implant biofilms in vivo stained with live/dead dyes after treatment with PBS, MnO₂, PMS, or MnO₂@PMS. Green: live bacteria; red: dead bacteria. Orthogonal views show biofilm thickness and viability distribution.

**
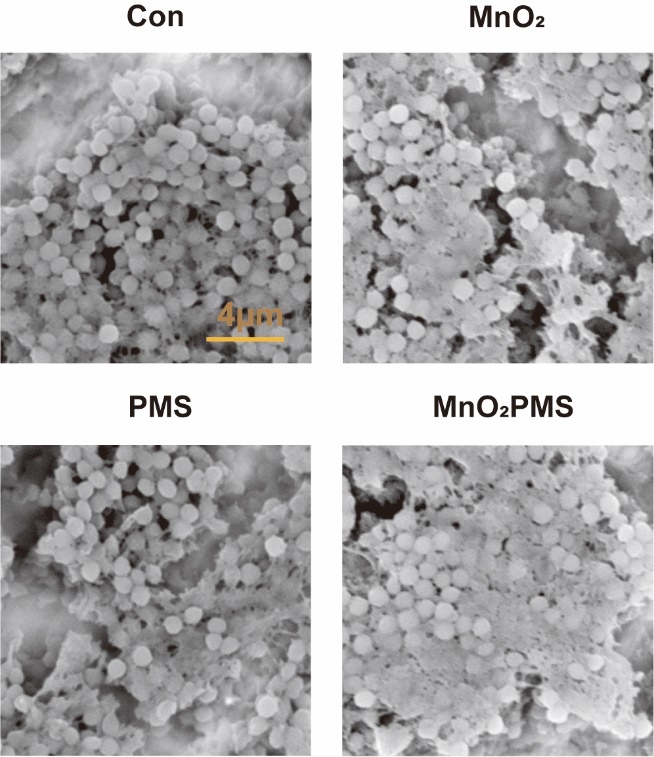
**

**Figure S30.** SEM images of *S. aureus* biofilms on murine implants after treatment under dark conditions. All groups showed intact biofilm morphology. Scale bar: 4 μm.

**
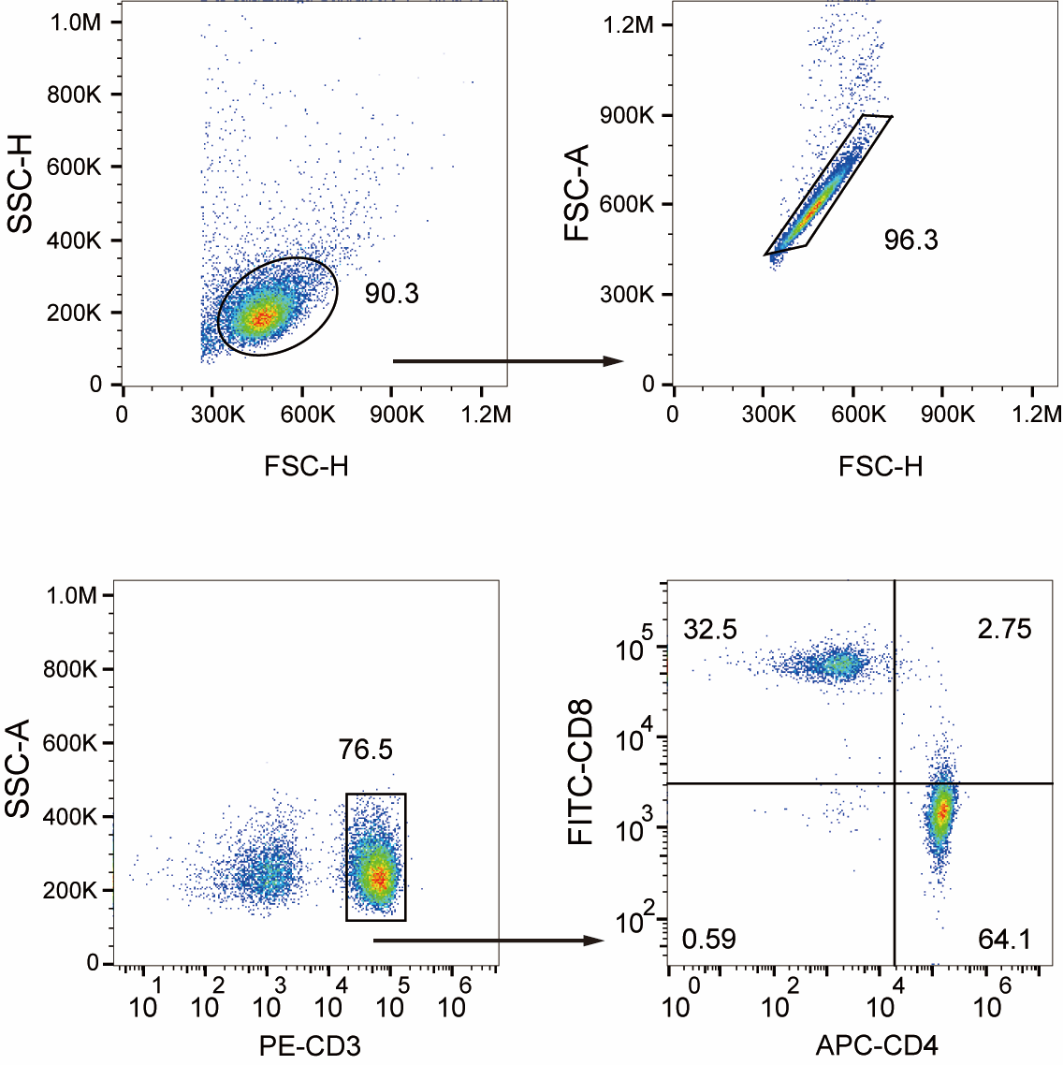
**

**Figure S31.** Representative gating strategy of CD4+ and CD8+ T cells in lymph nodes of the implant-associated infection mice on day 14 after NPs and laser treatment.

**
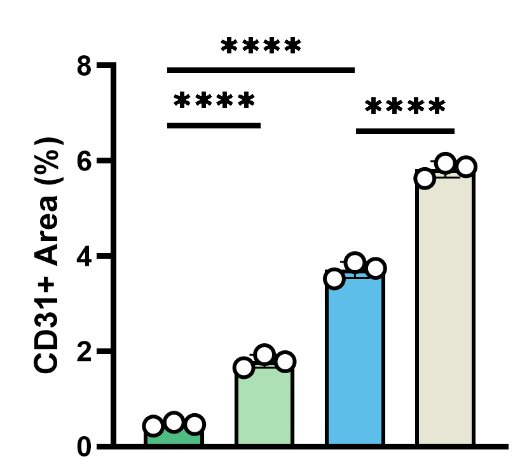
**

**Figure S32.** CD31⁺ IHC analysis of peri-implant tissues on Day 14 after treatment. MnO₂@PMS group showed the highest CD31⁺ area among groups. Quantification performed via ImageJ.


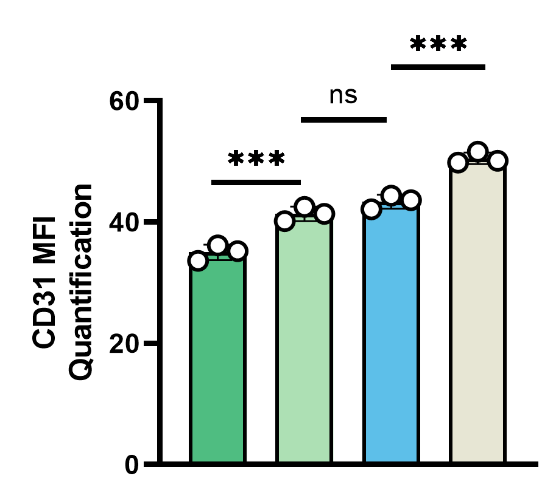


**Figure S33.** MFI quantification of CD31⁺ immunofluorescence in peri-implant tissues on Day 14 post-treatment with PBS, MnO₂, PMS, or MnO₂@PMS. Signal intensity quantified via ImageJ.

**
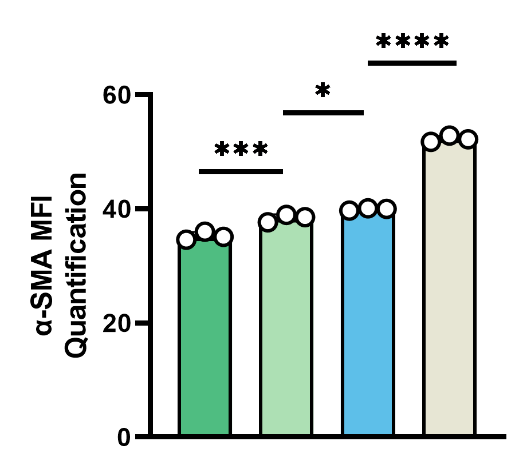
**

**Figure S34.** MFI quantification of α-SMA⁺ immunofluorescence in peri-implant tissues on Day 14 post-treatment with PBS, MnO₂, PMS, or MnO₂@PMS. Signal intensity quantified via ImageJ.


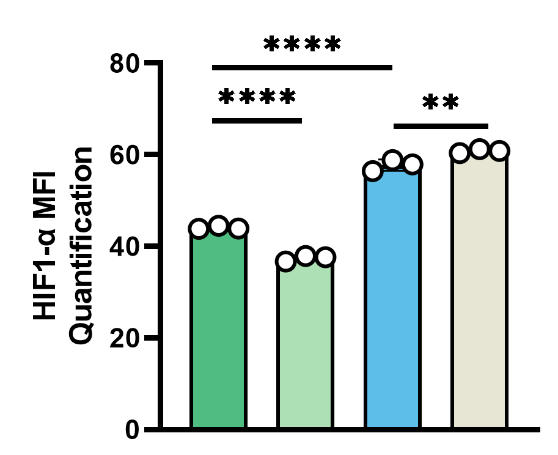


**Figure S35.** MFI quantification of HIF-1α immunofluorescence in peri-implant tissues on Day 14 post-treatment with PBS, MnO₂, PMS, or MnO₂@PMS. Signal intensity quantified via ImageJ.


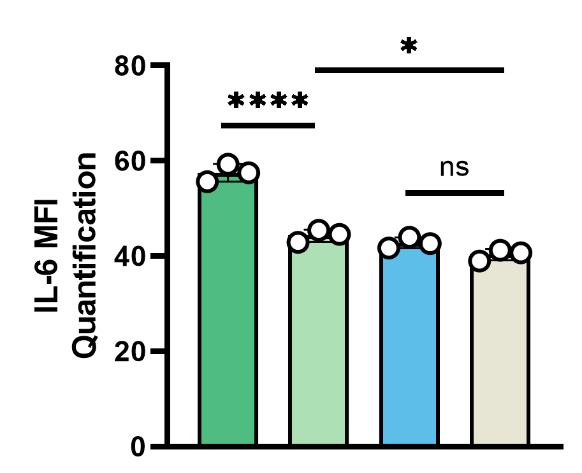


**Figure S36.** MFI quantification of IL-6 immunofluorescence in peri-implant tissues on Day 14 post-treatment with PBS, MnO₂, PMS, or MnO₂@PMS. Signal intensity quantified via ImageJ.


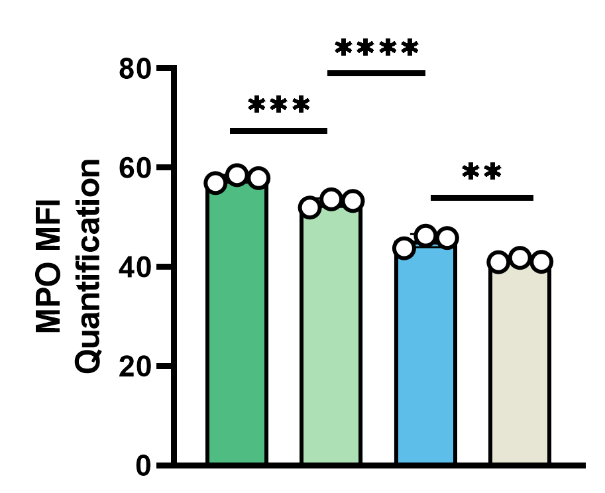


**Figure S37.** MFI quantification of MPO immunofluorescence in peri-implant tissues on Day 14 post-treatment with PBS, MnO₂, PMS, or MnO₂@PMS. Signal intensity quantified via ImageJ.

**Table S1. Flow cytometry antibodies used in this study.**

| **Fluorescence** | **Antibody** | **Clone** | **Company (Cat.#)** | **Dilution** |
| --- | --- | --- | --- | --- |
| / | CD16/32 | 93 | Biolegend (101320) | 1:100 |
| APC | CCR7 | 4B12 | Biolegend (120107) | 1:100 |
| PE | CD206 | C068C2 | Biolegend (141706) | 1:100 |
| FITC | CD11c | N418 | Biolegend (117305) | 1:100 |
| PE/cy7 | CD80 | 16-10A1 | Biolegend (104734) | 1:100 |
| APC | CD86 | GL-1 | Biolegend (105012) | 1:100 |
| APC | CD3 | 17A2 | Biolegend (100236) | 1:100 |
| FITC | CD4 | GK1.5 | Biolegend (100405) | 1:100 |
| PE/cy7 | CD8 | 53-6.7 | Biolegend (100721) | 1:100 |

**Table S2. Western blot antibodies used in this study.**

| **Target** | **Host** | **Clone** | **Company (Cat.#)** | **Dilution** |
| --- | --- | --- | --- | --- |
| STING | Rabbit | D2P2F | CST (13647S) | 1:1000 |
| TBK1 | Rabbit | D1B4 | CST (3504S) | 1:1000 |
| p-TBK1 | Rabbit | D52C2 | CST (5483S) | 1:1000 |
| IRF3 | Rabbit | D83B9 | CST (4302S) | 1:1000 |
| p-IRF3 | Rabbit | 4D4G | CST (4947S) | 1:1000 |
| p65 | Rabbit | D14E12 | CST (8242S) | 1:1000 |
| p-p65 | Rabbit | 93H1 | CST (3033S) | 1:1000 |
| GADPH | Rabbit | D16H11 | CST (5174S) | 1:1000 |

Note: All primary antibodies listed above were detected using HRP-conjugated goat anti-rabbit IgG secondary antibody (CST, Cat. No. 7074S, 1:3000 dilution).

**Table S3. Primers used in this study.**

| **Primer** | **Sequence (5′−3′)** |
| --- | --- |
| Timd4-F | GGCACCACCTCTGACACTTCTTATG |
| Timd4-R | CCAGACCAAGCCTTCCAAGACATC |
| CD206-F | CCTATGAAAATTGGGCTTACGG |
| CD206-R | CTGACAAATCCAGTTGTTGAGG |
| H2-D-F | GGCTGTCCTGGAACTCACTTTGTAG |
| H2-D-R | AGCAGTCTCCTCTGGCACCTATG |
| CD40-F | GCCCTGTTTCTGTCTGTCTGTCTG |
| CD40-R | ACTGCCTCTTGGTCTCACTCCTATC |
| Il6-F | CTGCAAGAGACTTCCATCCAG |
| Il6-R | AGTGGTATAGACAGGTCTGTTGG |
| Cxcl10-F | CCAAGTGCTGCCGTCATTTTC |
| Cxcl10-R | GGCTCGCAGGGATGATTTCAA |
| TNF-F | CAGGCGGTGCCTATGTCTC |
| TNF-R | CGATCACCCCGAAGTTCAGTAG |
| Ifit2-F | GGAGAGCAATCTGCGACAG |
| Ifit2-R | GCTGCCTCATTTAGACCTCTG |
| Cxcl9-F | GGAGTTCGAGGAACCCTAGTG |
| Cxcl9-R | GGGATTTGTAGTGGATCGTGC |
| Ccl2-F | TAAAAACCTGGATCGGAACCAAA |
| Ccl2-R | GCATTAGCTTCAGATTTACGGGT |
| Sting-F | TCGCACGAACTTGGACTACTG |
| Sting-R | CCAACTGAGGTATATGTCAGCAG |
| Ifnb1-F | CAGCTCCAAGAAAGGACGAAC |
| Ifnb1-R | GGCAGTGTAACTCTTCTGCAT |
| GADPH-F | AGGTCGGTGTGAACGGATTTG |
| GADPH-R | TGTAGACCATGTAGTTGAGGTCA |
